# Oxindole synthesis *via* polar–radical crossover of ketene-derived amide enolates in a formal [3 + 2] cycloaddition†

**DOI:** 10.1039/d1sc07134c

**Published:** 2022-03-09

**Authors:** Niklas Radhoff, Armido Studer

**Affiliations:** Organisch-Chemisches Institut, Westfälische Wilhelms-Universität Corrensstraße 40 48149 Münster Germany studer@uni-muenster.de

## Abstract

Herein we introduce a simple, efficient and transition-metal free method for the preparation of valuable and sterically hindered 3,3-disubstituted oxindoles *via* polar–radical crossover of ketene derived amide enolates. Various easily accessible *N*-alkyl and *N*-arylanilines are added to disubstituted ketenes and the resulting amide enolates undergo upon single electron transfer oxidation a homolytic aromatic substitution (HAS) to provide 3,3-disubstituted oxindoles in good to excellent yields. A variety of substituted anilines and a 3-amino pyridine engage in this oxidative formal [3 + 2] cycloaddition and cyclic ketenes provide spirooxindoles. Both substrates and reagents are readily available and tolerance to functional groups is broad.

## Abstract 2

Herein we introduce a simple, efficient and transition-metal free method for the preparation of valuable and sterically hindered 3,3-disubstituted oxindoles *via* polar–radical crossover of ketene derived amide enolates.

## Graphical abstract

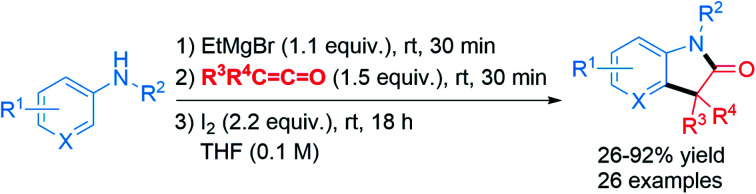

Oxindoles, in particular the 3,3-disubstituted congeners, are highly valuable substructures in medicinal chemistry. The oxindole core can be found in various biologically active compounds, that are for example used in the treatment of cancer or as antibacterial agents.^1^ In addition, the oxindole moiety also occurs in several complex natural products.^2^ The first oxindole synthesis was reported by Baeyer and Knop in 1866.^3^ That time, isatin was converted by sodium amalgam reduction to the corresponding oxindole. Since then, many methods for the preparation of 3,3-disubstituted oxindoles have been developed that proceed *via* functionalization of a pre-existing oxindole core.^4^ In addition, methods for the construction of 3,3-disubstituted oxindoles starting from acyclic precursors have also been introduced.^5,6^ Along these lines, transition metal-mediated reactions^5,7^ or homolytic aromatic substitutions (HAS)^8–14^ have found to be highly efficient for the construction of the oxindole core. Focusing on the latter approach, the intramolecular HAS proceeds *via* α-carbonyl radicals derived from radical addition to *N*-arylacrylamides,^8^ reduction of α-haloarylamides^9^ or oxidation of the corresponding enolates^10–14^ (Scheme 1a).

**Scheme 1:**
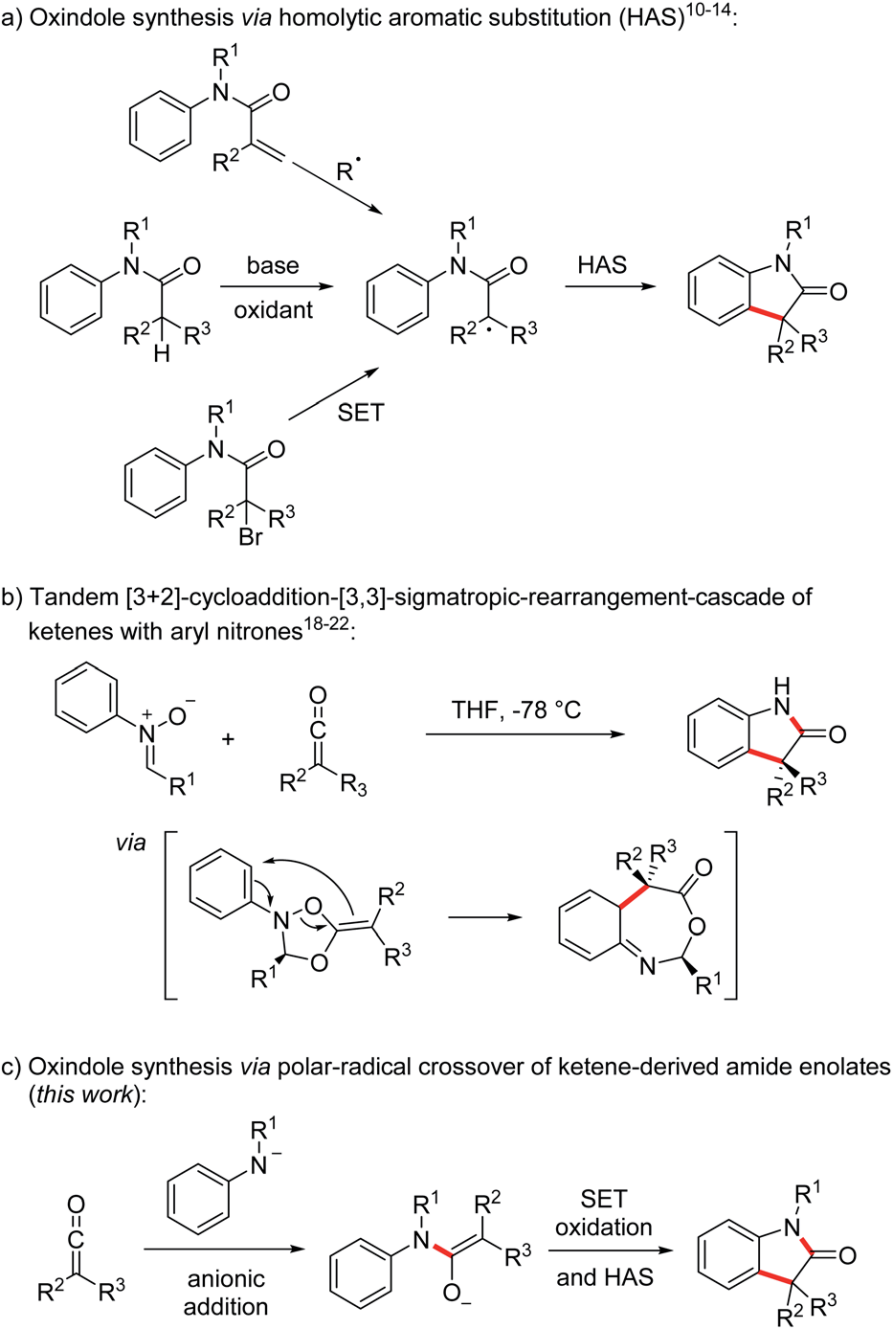
Selected strategies for the synthesis of oxindoles.

In 2017, the group of Taylor developed a transition metal-free enolate oxidation-HAS-approach towards oxindoles at low temperature using elemental iodine as the oxidant and malonic acid derived *N*-aryl amides as substrates which are readily deprotonated.^14^

The unique reactivity of ketenes^15^ has been explored extensively,^16^ especially in [2 + 2]-cycloadditions.^17^ Moreover, Staudinger,^18^ Lippman^19^ and Taylor^20^ showed that ketenes react with aryl nitrones in a tandem [3 + 2]-cycloaddition-[3,3]-sigmatropic-rearrangement cascade^21^ followed by hydrolysis to provide oxindoles (Scheme 1b). The use of chiral nitrones leads to chirality transfer and enantiomerically enriched oxindoles can be obtained *via* this approach.^21,22^ In contrast to the examples discussed in Scheme 1a, two σ-bonds are formed and the overall sequence can be regarded as a formal [3 + 2] cycloaddition. Despite good yields and high enantiomeric excess, nitrones have to be used as precursors and an aldehyde is formed as the byproduct diminishing reaction economy of these elegant cascades.

To address these drawbacks, we decided to use the nucleophilic addition^23–26^ of deprotonated anilines to ketenes for the generation of the corresponding amide enolates that should then be oxidized in a single electron transfer process to α-amide radicals which can undergo a homolytic aromatic substitution providing direct access to sterically challenging 3,3-disubstituted oxindoles in a straightforward one-pot sequence (Scheme 1c). This polar–radical crossover reaction shows high atom economy and as the reaction with the nitrones can also be regarded as a formal [3 + 2] cycloaddition.

We initiated the optimization study with *N*-methylaniline 1a and ethyl phenyl ketene 2a, which was prepared in an easy and scalable one-pot protocol starting from the corresponding carboxylic acid, as model substrates. Deprotonation of 1a with *n-*BuLi in THF and subsequent addition to the ketene 2a led to desired Li-enolate which was confirmed by protonation with water and isolation of the amide 4aa (56%). Pleasingly, addition of ferrocenium hexafluorophosphate (FcPF_6_, 2.2 equiv.) at room temperature to the Li-enolate afforded the desired oxindole 3aa in 29% yield (Table 1, entry 1). Switching to CuCl_2_ (2.2 equiv.) as the oxidant increased the yield to 34% (Table 1, entry 2) and the use of iodine (2.2 equiv.) improved reaction efficiency (41%, Table 1, entry 3). A further increase in yield (44%) was achieved upon I_2_-oxidation of the corresponding Mg-enolate (Table 1, entry 4). Protonated enolate 4aa (23% yield) and the α,β-unsaturated amide 5aa (27%) were observed as the major side products in this transformation. In contrast to the Li-enolate discussed earlier, the intermediate Mg-enolate is formed almost quantitatively, which was confirmed by protonation with water and isolation of compound 4aa (91%). Lowering the reaction concentration to 0.02 M and 0.01 M increased the yield significantly to 78% and 90%, respectively (Table 1, entries 5 and 6). Decreasing the amount of oxidant to 1.2 equivalents led to a worse result (Table 1, entry 7). The use of a more electrophilic iodine source such as *N*-iodosuccinimide (NIS, 2.2 equiv.) also resulted in a lower yield of 39% (Table 1, entry 8). Notably, in this case, the α,β-unsaturated amide 5aa was formed as the major product in 60% yield. When the reaction temperature was lowered to −78 °C prior to the addition of iodine (1.2 equiv.), the desired oxindole 3aa was formed in 80% yield (Table 1, entry 9).^14^ Light does not appear to play a crucial role in this transformation, as performing the reaction in the dark does not have a significant effect on the reaction outcome (Table 1, entry 10). Irradiation with a blue LED (467 nm) actually decreased the yield of targeted 3aa to 74% (Table 1, entry 11). The reaction time of step 3 could be significantly reduced to two hours when the reaction was carried out in THF under reflux conditions, and the desired oxindole 3aa was formed in 93% yield (Table 1, entry 12).

**Table 1:** Optimization studies^a^

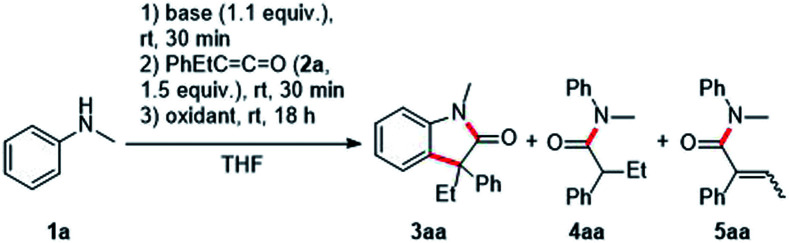				
Entry	Base	Conc. (M)	Oxidant (equiv.)	Yield 3aa (%)^b^
1	*n*-BuLi	0.1	FcPF_6_ (2.2)	29 (18)^c^
2^d^	*n*-BuLi	0.1	CuCl_2_ (2.2)	34^c^
3	*n*-BuLi	0.1	I_2_ (2.2)	41^c^
4	EtMgBr	0.1	I_2_ (2.2)	44^c^
5	EtMgBr	0.02	I_2_ (2.2)	78
**6**	**EtMgBr**	**0.01**	**I**_**2**_ **(2.2)**	**90 (82)** c
7	EtMgBr	0.01	I_2_ (1.2)	25
8	EtMgBr	0.01	NIS^e^ (2.2)	39
9	EtMgBr	0.01	I_2_ (1.2)^f^	80
10	EtMgBr	0.01	I_2_ (2.2)^g^	82
11	EtMgBr	0.01	I_2_ (2.2)^h^	74
12	EtMgBr	0.01	I_2_ (2.2)^i^	93

a Reactions (0.20 mmol) were conducted under argon atmosphere.

b ^1^H NMR yield using 1,3,5-trimethoxybenzene as internal standard.

c Isolated yield.

d Step 1 and 2 were conducted at 0 °C.

e *N*-Iodosuccinimide.

f Iodine addition at −78 °C, then slowly allowed to warm to room temperature.^14^

g In the dark.

h Irradiation with blue LED (40 W, 467 nm, rt, 8 h).

i Refluxing THF for step 3, reaction completed within 2 h.

With the optimized reaction conditions in hand, we investigated the scope by first varying the R^1^-substituent at the N-atom using the ketene 2a as the reaction partner (Scheme 2). In general, increasing the steric bulk at the nitrogen leads to diminished yields of the targeted oxindoles. The lower yields go along with the formation of a larger amount of the corresponding α,β-unsaturated amide side product 5. Thus, as compared to the parent *N*-methyl derivative, all other *N*-alkyl derivatives were formed in lower yields (49%, 3ab; 35%, 3ac; 49%, 3ad). The *N*-benzyl protected oxindole 3af and the *N*-phenyl oxindole 3ae were isolated in 54% and 56% yield, respectively. Next, a diastereoselective oxindole synthesis was attempted using chiral anilines 1g and 1h. Surprisingly, despite the bulkiness of these nucleophiles containing styryl-type *N*-substituents, good yields were obtained for the oxindoles 3ag and 3ah (73–79%). Unfortunately, diastereocontrol was low in both cases (1.9 : 1 d.r. and 1.5 : 1 d.r.). Of note, addition of Mg-1g and Mg-1h to ketene 2a was rather slow under the standard reaction condition and a significant amount of unreacted aniline was recovered. That problem could be solved by prolonging the reaction time of both step 1 (deprotonation) and also step 2 (Mg-enolate formation).

**Scheme 2:**
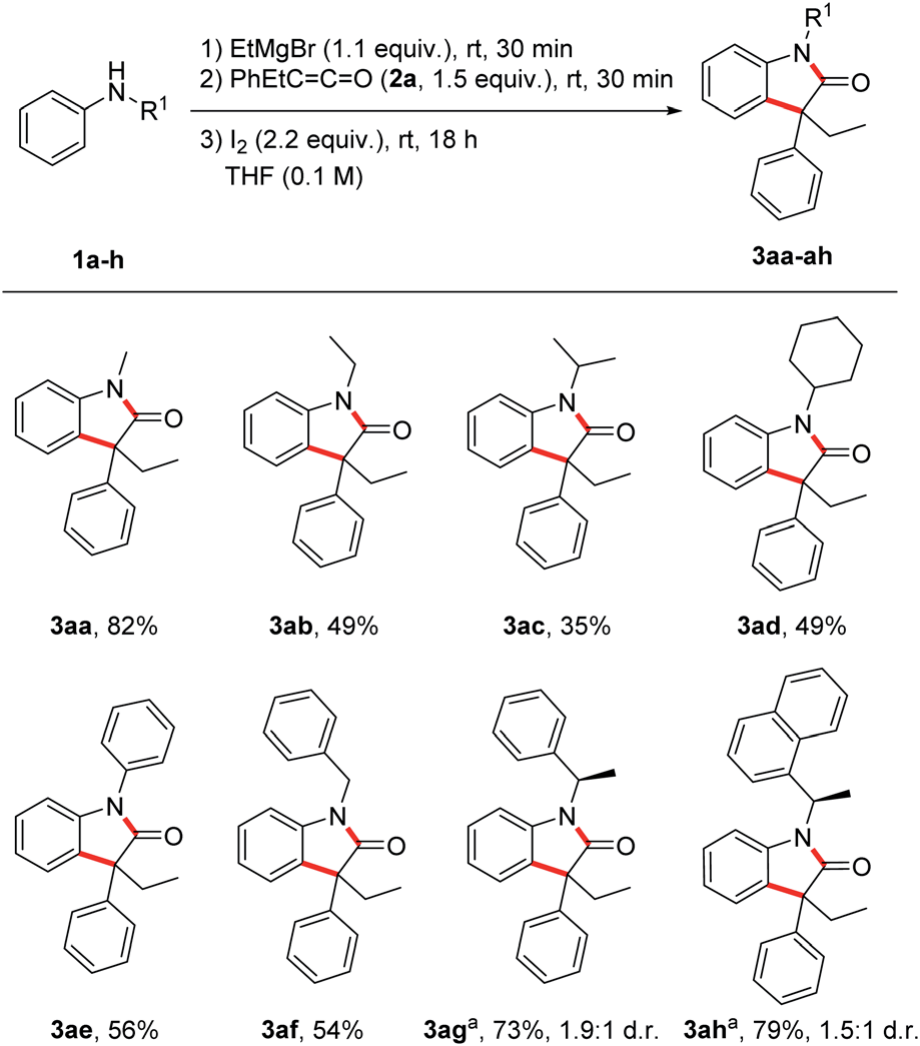
Substrate scope – variation of substituents at the nitrogen. Reactions (0.20 mmol) were conducted under argon atmosphere. ^*a*^ For step 1 and 2 reaction time was 1 h.

Next, the substrate scope was investigated by using different anilines in combination with the ketene 2a (Scheme 3). *N*-Methyl-*p*-toluidine 1i and *N*-methyl-*p*-haloanilines 1j–m could be successfully transformed to the corresponding oxindoles 3al–am in moderate to good yields (53–87%). Electron-withdrawing and also electron-donating substituents are tolerated and oxindoles derived from *p*-cyano- (3an, 92%), *p*-acetyl- (3ao, 30%), *p*-methoxycarbonyl- (3ap, 82%) and *p*-methoxy- (3aq, 71%) anilines were isolated in moderate to excellent yields documenting a high functional group tolerance of this reaction. The meta-methyl aniline afforded oxindole 3ar in 76% yield as a 1.8 : 1 mixture of the two regioisomers (only the major isomer drawn). For the pyridyl derivative 3as, a lower yield was obtained (39%), but reaction occurred with complete regiocontrol. Of note, *ortho*-methyl *N*-methylaniline provided the corresponding oxindole only in trace amounts (not shown).

**Scheme 3:**
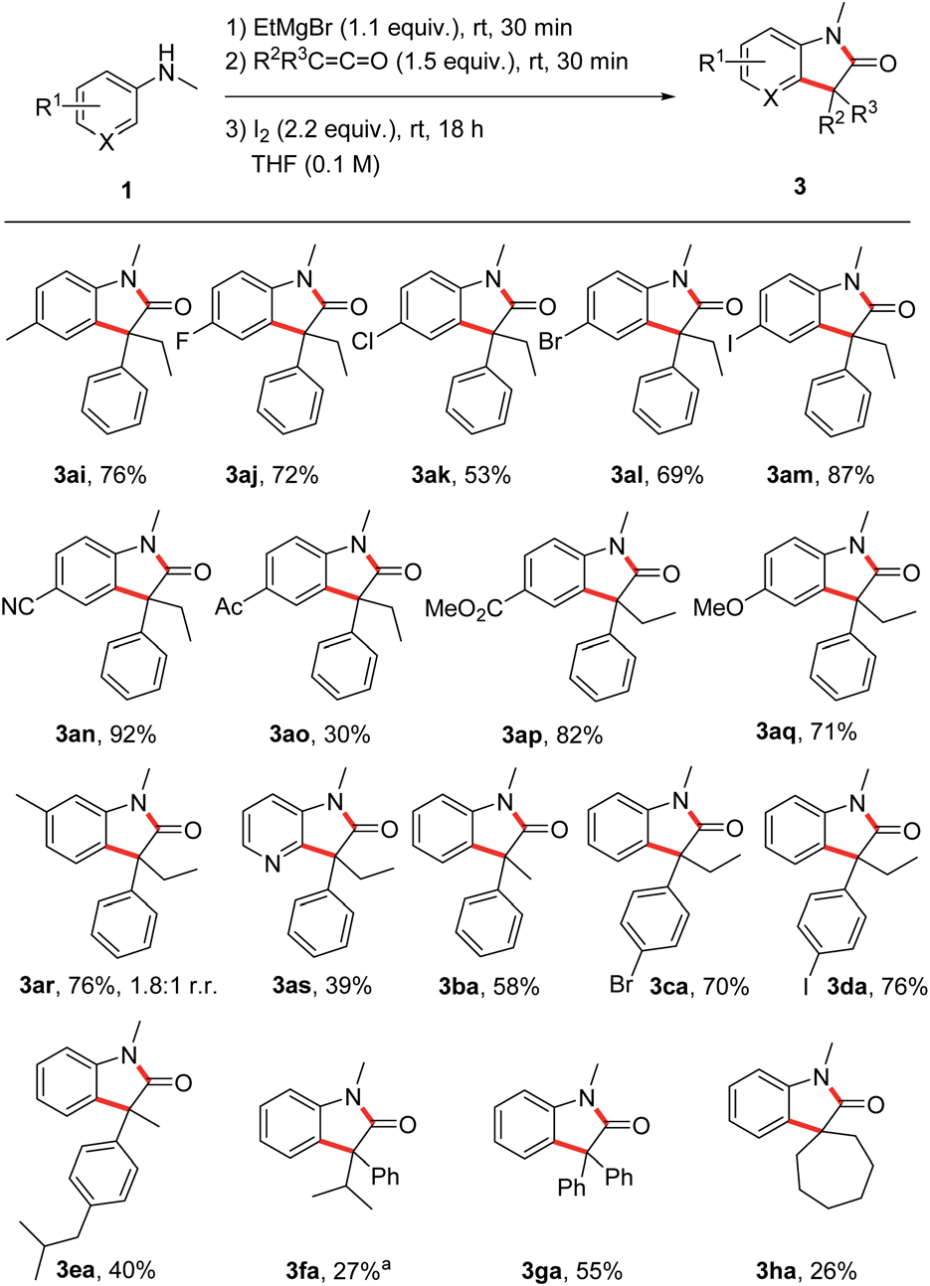
Substrate Scope – variation of anilines and ketenes. Reactions (0.20 mmol) were conducted under argon atmosphere. ^*a*^ Isolated as an inseparable mixture (1 : 1.4) with the protonated enolate 4fa (56% combined yield).

The ketene component was also varied using *N*-methylaniline 1a as the reaction partner. The transformation of methyl phenyl ketene 2b provided the oxindole 3ba in 58% yield. *p*-Bromophenyl ethyl ketene 2c and *p*-iodophenyl ethyl ketene 2d afforded the oxindoles 3ca and 3da in good yields (70% and 76%). For the ibuprofene-derived ketene 2e a lower yield was obtained (3ea, 40%) and the bulkier phenyl isopropyl congener 3fa was isolated in 27% yield as an inseparable mixture with the protonated enolate 4fa (56% combined yield). In the latter case, increasing the reaction time did neither lead to a higher yield of 3fa nor to a suppression of the formation of 4fa. The lower yield is likely caused by steric effects. Surprisingly, diphenyl ketene 2g delivered the targeted oxindole 3ga in acceptable 55% yield despite the steric demand of the two phenyl groups and the high stability of the corresponding α-amide radical. Spirocyclic oxindoles are of great interest due to their high pharmaceutical potential.^27^ We were pleased to find that our method also works for the preparation of such spiro compounds as documented by the successful synthesis of 3ha (26%).

Mechanistically, we propose initial formation of the enolate A by nucleophilic attack of the deprotonated aniline to the ketene 2, which is then oxidized by elemental iodine to the α-amide radical B (pathway b). The radical nature of the transformation is supported by the fact that electronic effects on the arene show no influence on the efficiency of the cyclization, as would be shown by a conceivable polar aromatic substitution. Radical B readily cyclizes onto the aniline ring to generate the cyclohexadienyl radical D which is oxidatively rearomatized *via* cationic intermediate E to finally give the oxindole 3 (Scheme 4).^10–14^ Alternatively, enolate A can be iodinated with I_2_ to give the unstable iodide C which then undergoes C–I bond homolysis to generate the radical B (pathway a). Indeed, Taylor and coworkers^14^ observed under similar reaction conditions the decay of α-iodinated compounds of type C*via* C–I homolysis^14,28^ to give radicals of type B. Usually, we observed α,β-unsaturated amides analogous to 5aa as by-products. However, the corresponding protonated enolates were detected only in tiny amounts in most of these cases. This strongly suggests that those amides are not formed *via* disproportionation of radical B. HI-elimination seems more likely, pointing towards the presence of the iodinated species C and thus the contribution of pathway b to product formation. In addition, dimerization of radical B was also not observed.

**Scheme 4:**
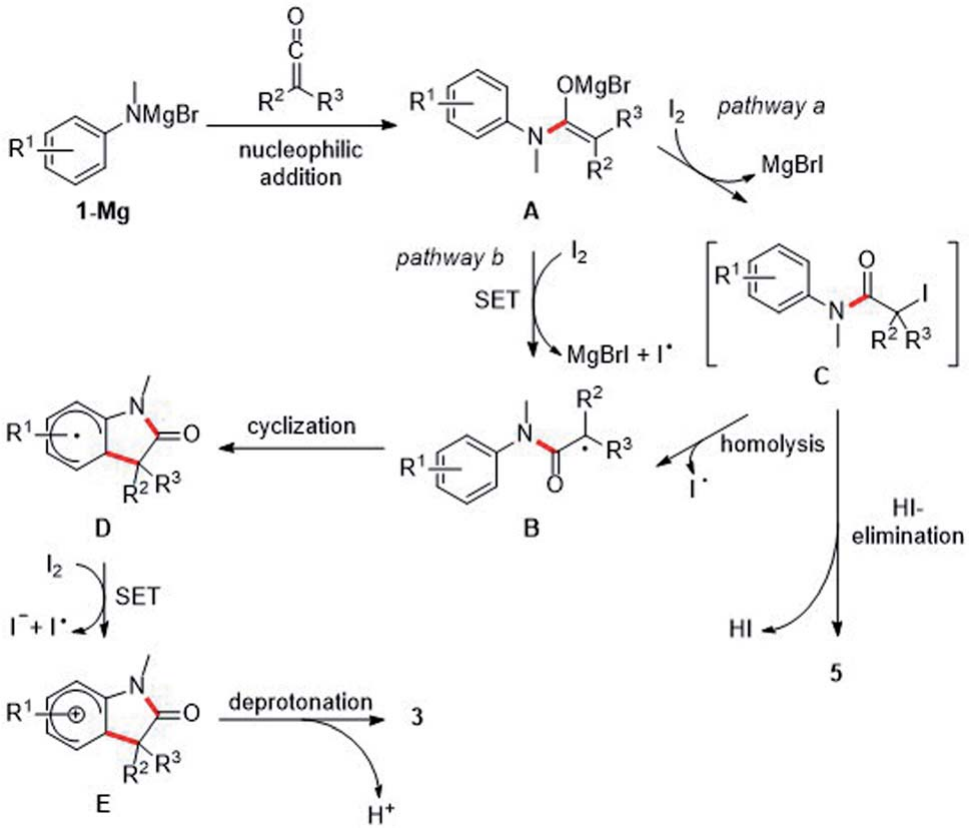
Suggested mechanism.

To further support pathway b, isolation of the iodinated intermediate C was attempted at low temperature. Upon addition of iodine (1.2 equiv.) to the preformed Mg-enolate A derived from aniline 1a and ketene 2a at −78 °C,^14^ TLC analysis showed a clean conversion to a single new compound, which was analyzed by rapid ESI-MS analysis and provided evidence for the formation of the iodinated intermediate C (Scheme 5). However, isolation of this highly unstable compound was not possible due to rapid HI-elimination to the amide 5aa. Note that oxindole formation worked well upon I_2_-addition at −78 °C and subsequent warming to room temperature (see Table 1, entry 9). In contrast to the unstable tertiary iodide C, the secondary iodide 7 proved to be stable at room temperature as well as in refluxing THF in the absence of light. Due to the stronger C–I bond of 7 as compared to its tertiary congeners, thermal activation of this iodide is not possible. Upon irradiation of 7 with a blue LED (467 nm), the compound decomposed to release iodine without producing the expected oxindole product (Scheme 5). This is consistent with the observation from our optimization studies that irradiation with blue light does not contribute to the yield of oxindole 3aa (Table 1, entry 11). Furthermore, the use of more electrophilic NIS instead of iodine should favor the formation of intermediates of type C over direct SET. In this case, the elimination product 5aa was obtained as the main product (60% yield) and the oxindole 3aa was formed in only 39% yield (Table 1, entry 8). In light of these results, we suggest that both the direct SET of Mg-enolate A to iodine (pathway a) and the C–I bond homolysis of intermediate C (pathway b) might operate in these transformations.

**Scheme 5:**
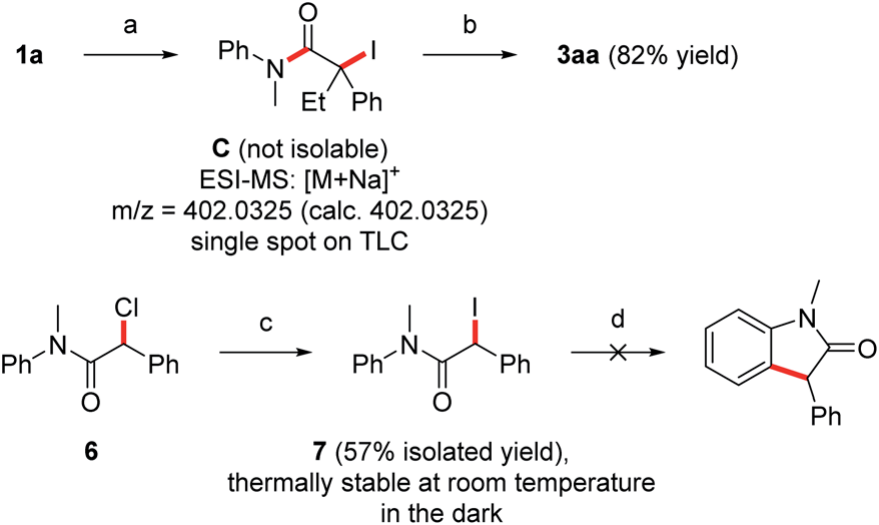
Mechanistic experiments. (a) (1) EtMgBr (1.1 equiv.), rt, 30 min, (2) 2a (1.5 equiv.), −78 °C, 30 min, (3) I_2_ (1.2 equiv.), −78 °C, 15 min in THF (0.01 M). (b) Warm to room temperature in THF (0.01 M), 18 h. (c) NaI (1.2 equiv.) in acetone (0.77 M), rt, 18 h. (d) Irradiation with blue LED (40 W, 467 nm) in THF (0.01 M), rt, 8 h.

## Conclusions

In conclusion, we demonstrated a simple, efficient and transition metal-free procedure for the preparation of sterically challenging valuable 3,3-disubstituted oxindoles *via* a polar–radical crossover of ketene-derived aniline enolates followed by homolytic aromatic substitution at room temperature starting from mostly commercially available anilines and ketenes, which are easily prepared in one-pot reactions from a plethora of commercially available compounds. 26 different (hetero)aromatic 3,3-oxindoles as well as spirooxindoles could be prepared following this method. Functional group tolerance is broad and the herein reported cascades nicely document the potential of polar–radical cross-over chemistry by benefiting from both ionic as well as from radical bond forming reactions.

## Data availability

The data that support the findings of this study are available in the ESI† or on request from the corresponding author.

## Author contributions

N. R. conducted all experiments and characterized the novel compounds. N. R. and A. S. designed the experiments and wrote the manuscript.

## Conflicts of interest

There are no conflicts to declare.

## Supplementary Material

SC-013-D1SC07134C-s001
