# Supplementary material for: Oxindole synthesis via polar–radical crossover of ketene-derived amide enolates in a formal [3 + 2] cycloaddition
Source: Chem Sci. 2022 Mar 9;13(13):3875–9. doi: 10.1039/d1sc07134c (PMC8966637; doi:10.1039/d1sc07134c)
Supplement: SC-013-D1SC07134C-s001 [file SC-013-D1SC07134C-s001.pdf]

Electronic Supplementary Material (ESI) for Chemical Science.
This journal is © The Royal Society of Chemistry 2022

Supporting Information

Oxindole Synthesis *via* Polar-Radical-Crossover of Ketene-derived Amide Enolates in a Formal [3+2] Cycloaddition

Niklas Radhoff and Armido Studer*

Organisch-Chemisches Institut, Westfälische Wilhelms-Universität, Corrensstrasse 40, 48149 Münster (Germany)

Email: studer@uni-muenster.de

Table of Contents

1. General information 1

2. General procedures 2

3. Analytical Data of compounds 4

3.1 Synthesis of *N*-monosubstituted anilines 4

3.2 Synthesis of Ketenes 8

3.3 Synthesis of Oxindoles 12

3.4 Synthesis of Amides 28

4. NMR Data of compounds 31

5. ESI(+)-MS spectra of intermediate **C** 79

6. References 80

1. General information

Reactions with compounds sensitive to air or moisture were conducted in oven-dried glassware under an atmosphere of argon using standard Schlenk-technique. Anhydrous tetrahydrofuran (THF) was refluxed over sodium and freshly distilled from potassium prior to use. Anhydrous diethyl ether (Et_2O) was freshly distilled from Na/K-alloy. Anhydrous dichloromethane (CH_2Cl_2) was dried over P_4O_{10} and also freshly distilled prior to use. Anhydrous methanol (MeOH) and dimethyl sulfoxide (DMSO) were purchased in extra-dry grade from *Acros Organics* and stored over molecular sieves. Unless stated otherwise, all reagents were purchased at *Sigma Aldrich*, *Acros Organics*, *Alfa Aesar, ABCR, TCI, Fluorochem* and *BLDPharm* and were used without further purification. Flash column chromatography (FC) was carried out on *Merck* silica gel (40-63 µm) with an excess compressed air pressure up to 0.5 bar. Solvents for chromatography and liquid-liquid extractions were purchased in technical grade and purified by distillation. For analytical thin layer chromatography (TLC) *Merck silica gel 60 F254*-plates were used and detection was carried out using UV-light (254 nm) or $KMNO_4$-stain (1.5 g in 250 mL water, 5 g $NaHCO_3$). Automatic flash-systems by *Reveleris IES* and *Büchi C-850 Flashprep* were used for medium pressure liquid chromatography (MPLC) using commercially available 4 g, 12 g or 40 g *Reveleris*-C_{18}-flash cartridges as the stationary phase. Preparative HPLC was conducted on a *Büchi C-850 Flashprep* device using an *Agilent Zorbax XDB-C18* column (21.2 x 150 mm, 5 µm particle size) as the stationary phase. As the mobile phase, water (*Milli-Q* grade*)*, acetonitrile (HPLC grade) and methanol (technical grade, distilled prior to use) were used. Detection was performed by UV-absorption (λ = 210 nm, 230, 254, 320 nm) and electronic light scattering detection (ELSD) with isopropanol as carrier. Melting points were measured using a *Büchi Melting Point M-560* device and are uncorrected. Infrared spectra were recorded on a *Digilab 3100 FT-IR Excalibur Series* and a *Jasco FT/IR-4600* spectrometer. Absorption bands are given in wave numbers ν (cm^{-1}). HRMS spectra (ESI-MS) were recored on a *Thermo Fisher Scientific Orbitrap Velos Pro* and *Thermo Fisher Scientific LTQ Orbitrap XL* spectrometer. ^{1}H NMR (300 MHz, 400 MHz, 500 MHz and 600 MHz), ^{13}C NMR (75 MHz, 101 MHz, 126 MHz and 151 MHz) and ^{19}F NMR (282 MHz) spectra were measured on a *Bruker Avance II 300, Bruker NEO 400, Agilent DD2 500* and *Agilent DD2 600* spectrometer. Chemical shifts (δ in ppm) were referenced to the solvent residual peak ($CDCl_3$: δ_H = 7.26 ppm and δ_c = 77.0 ppm; DMSO-d_6: δ_H = 2.50 ppm and δ_c = 39.5 ppm). The multiplets of the observed signals were given as s (singlet), bs (broad signal), d (doublet), t (triplet), q (quartet), hept (heptet), m (multiplet) and combination of the above.

2. General procedures

General Procedure (GP1) for the preparation of *N*-monosubstituted anilines (1g-ah):

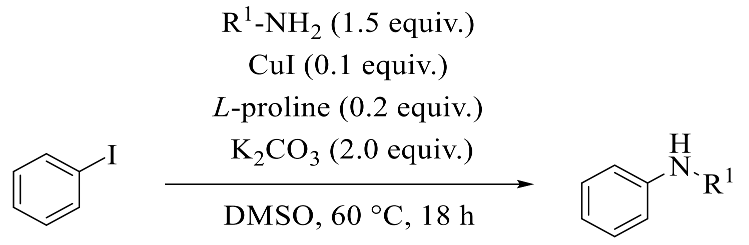

According to a procedure by *Ma et al.*[1], iodobenzene (1.0 equiv., 10.0 mmol), a primary amine (1.5 equiv.), copper iodide (0.1 equiv.), *L*-proline (0.2 equiv.) and potassium bicarbonate (2.0 equiv.) were dissolved in anhydrous DMSO (0.5 M) and heated to 60 °C for 18 hours. After cooling to room temperature, water and EtOAc were added and the phases were separated. The aqueous layer was extracted with EtOAc (3 x), the combined organic phases were dried over $MgSO_4$ and concentrated *in vacuo*. Purification by flash column chromatography (EtOAc/pentane) led to isolation of the desired *N*-monosubstituted anilines.

General Procedure (GP2) for the preparation of *N*-Methylanilines (1l-n and 1p-q):

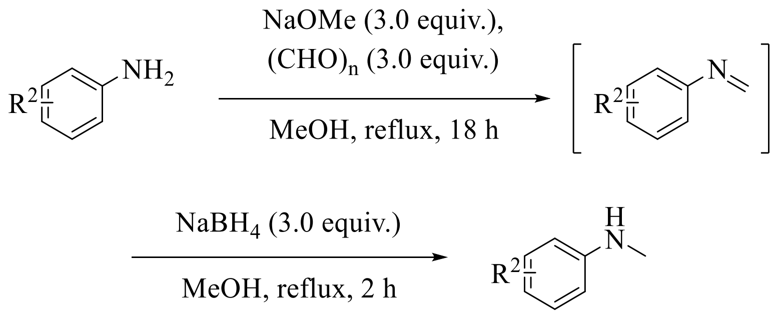

According to a procedure by *Barluenga et al.*[2], an aniline (1.0 equiv., 5.00 mmol), NaOMe (3.0 equiv.) and paraformaldehyde (3.0 equiv.) were dissolved in anhydrous MeOH (0.4 M) and refluxed overnight. After cooling to room temperature, sodium borohydride (3.0 equiv.) was added in three portions over 15 minutes. The reaction mixture was refluxed for two hours, cooled to room temperature and quenched with NH_4Cl-solution (sat. aq.) and EtOAc. The phases were separated and the aqueous phase was extracted with EtOAc (3 x). The combined organic phases were dried over $MgSO_4$ and concentrated *in vacuo*. Purification by flash column chromatography (EtOAc/pentane) led to isolation of the desired *N*-methylanilines.

General Procedure (GP3) for the preparation of Ketenes (2a-h):

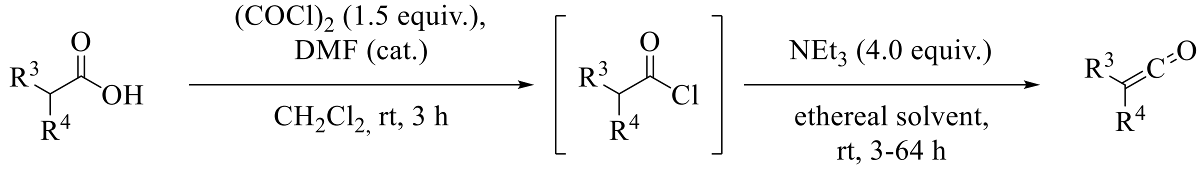

Following a representative procedure from the literature[3], a carboxylic acid (1.0 equiv., between 10.0 mmol and 20.0 mmol) was dissolved in anhydrous CH_2Cl_2 (approx. 0.1 M). Oxalyl chloride (1.5 equiv.) and catalytic amounts of anhydrous DMF were added at room temperature. When gas formation ceased (approx. 3 h), the volatiles were removed *in vacuo* and the residue was dissolved in anhydrous ethereal solvent (THF or Et_2O, depending on the boiling point of the ketene). Triethylamine (4.0 equiv.) was added slowly at room temperature and the reaction mixture was stirred for 3 – 64 hours. Filtration under Argon atmosphere, concentration *in vacuo* and short-path distillation gave the desired ketenes as yellow oils.

General Procedure (GP4) for the preparation of Oxindoles (3aa-ha):

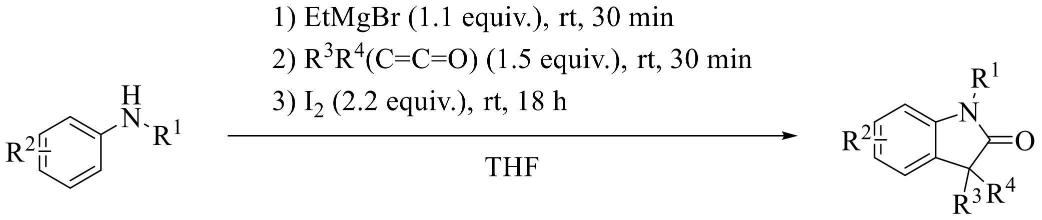

To a solution of a *N*-monosubstituted aniline (1.0 equiv., 0.20 mmol) in anhydrous THF (0.01 M) ethyl magnesium bromide (3.0 M solution in Et_2O, 1.1 equiv.) was added at room temperature and the reaction mixture was stirred for 30 minutes. Subsequently, the ketene (1.5 equiv.) was added dropwise and stirring was continued. After further 30 minutes, iodine (2.2 equiv.) was added and the reaction was stirred overnight at room temperature. Sodium sulfite (sat. aq. solution) was added until the brown colour disappeared and the phases were separated. The aqueous phase was extracted with EtOAc (3 x), the combined organic phases dried over $MgSO_4$ and concentrated *in vacuo*. Purification by *reversed-phase* MPLC ($MeOH/H_2O$ or $MeCN/H_2O$) led to isolation of the desired oxindoles.

3. Analytical Data of compounds

3.1 Synthesis of *N*-monosubstituted anilines

(*R*)-*N*-(1-Phenylethyl)aniline (1g):

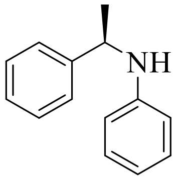

The title compound was prepared following the general procedure **GP1** using iodobenzene (1.12 mL, 10.0 mmol, 1.0 equiv.), (*R*)-1-phenylethan-1-amine (1.91 mL, 15.0 mmol, 1.5 equiv.), CuI (191 mg, 1.00 mmol, 0.1 equiv.), *L*-proline (230 mg, 2.00 mmol, 0.2 equiv.) and K_2CO_3 (2.76 g, 20.0 mmol, 2.0 equiv.) in DMSO (20 mL). Purification by flash column chromatography (EtOAc/pentane, *v/v* = 1:19) led to isolation of the title compound as a colourless solid (1.67 g, 8.48 mmol, 85%).

^{1}H NMR (300 MHz, $CDCl_3$, 300 K): δ_H (ppm) = 7.47 – 7.27 (m, 5H), 7.21 – 7.14 (m, 2H), 6.76 – 6.70 (m, 1H), 6.62 – 6.57 (m, 2H), 4.56 (q, *J* = 6.7 Hz, 1H), 4.23 (bs, 1H), 1.59 (d, *J* = 6.7 Hz, 3H).

^{13}C NMR (75 MHz, $CDCl_3$, 300 K): δ_C (ppm) = 147.2 (C_q), 145.2 (C_q), 129.2 (*C*H), 128.7 (*C*H), 127.0 (*C*H), 126.0 (*C*H), 117.4 (*C*H), 113.5 (*C*H), 53.6 (*C*H), 25.1 (CH_3).

HRMS (ESI) *m/z* = 198.1277 calcd. for $[C_{14}H_{15}NH]^+$ $[M+H]^+$, found: 198.1277.

The analytical data are consistent with those reported in literature.[4]

(*R*)-*N*-(1-(Naphthalen-1-yl)ethyl)aniline (1h):

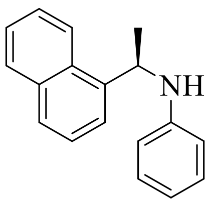

The title compound was prepared following the general procedure **GP1** using iodobenzene (1.12 mL, 10.0 mmol, 1.0 equiv.), (*R*)-1-(naphthalen-1-yl)ethan-1-amine (2.41 mL, 15.0 mmol, 1.5 equiv.), CuI (191 mg, 1.00 mmol, 0.1 equiv.), *L*-proline (230 mg, 2.00 mmol, 0.2 equiv.) and K_2CO_3 (2.76 g, 20.0 mmol, 2.0 equiv.) in DMSO (20 mL). Purification by flash column chromatography (EtOAc/pentane, *v/v* = 1:19) led to isolation of the title compound as a colourless solid (1.67 g, 8.48 mmol, 85%).

^{1}H NMR (300 MHz, $CDCl_3$, 300 K): δ_H (ppm) = 8.19 (d, *J* = 8.2 Hz, 1H), 7.95 – 7.92 (m, 1H), 7.77 (d, *J* = 8.2 Hz, 1H), 7.68 (d, *J* = 7.2 Hz, 1H), 7.61 – 7.51 (m, 2H), 7.45 – 7.40 (m, 1H), 7.11 – 7.06 (m, 2H), 6.68 – 6.63 (m, 1H), 6.52 – 6.49 (m, 2H), 5.31 (q, *J* = 6.7 Hz, 1H), 4.19 (s, 1H), 1.69 (d, *J* = 6.7 Hz, 3H).

^{13}C NMR (75 MHz, $CDCl_3$, 300 K): δ_C (ppm) = 147.1 (C_q), 139.9 (C_q), 134.1 (C_q), 130.7 (C_q), 129.2 (*C*H), 127.5 (*C*H), 126.1 (*C*H), 125.9 (*C*H), 125.5 (*C*H), 122.6 (*C*H), 122.3 (*C*H), 117.2 (*C*H), 113.2 (*C*H), 49.5 (*C*H), 23.7 (CH_3).

HRMS (ESI) *m/z* = 248.1434 calcd. for $[C_{18}H_{17}NH]^+$ $[M+H]^+$, found: 248.1436.

The analytical data are consistent with those reported in literature.[5]

4-Bromo-*N*-methylaniline (1l):

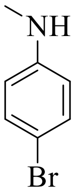

The title compound was prepared following the general procedure **GP2** using 4-bromoaniline (860 mg, 5.00 mmol, 1.0 equiv.), NaOMe (810 mg, 15.0 mmol, 3.0 equiv.), paraformaldehyde (451 mg, 15.0 mmol, 3.0 equiv.) and $NaBH_4$ (568 mg, 15.0 mmol, 3.0 equiv.) in MeOH (12.5 mL). Purification by flash column chromatography (EtOAc/pentane, *v/v* = 1:9) led to isolation of the title compound as a brown oil (818 mg, 4.40 mmol, 88%).

^{1}H NMR (300 MHz, $CDCl_3$, 300 K): δ_H (ppm) = 7.31 – 7.27 (m, 2H), 6.52 – 6.48 (m, 2H), 3.74 (s, 1H), 2.82 (s, 3H).

^{13}C NMR (75 MHz, $CDCl_3$, 300 K): δ_C (ppm) = 148.3 (C_q), 131.9 (*C*H), 114.0 (*C*H), 108.7 (C_q), 30.7 (CH_3).

HRMS (ESI) *m/z* = 185.9913 calcd. for $[C_7H_8NBrH]^+$ $[M+H]^+$, found: 185.9912.

The analytical data are consistent with those reported in literature.[6]

4-Iodo-*N*-methylaniline (1m):

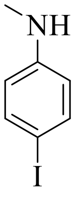

The title compound was prepared following the general procedure **GP2** using 4-iodoaniline (1.10 g, 5.00 mmol, 1.0 equiv.), NaOMe (810 mg, 15.0 mmol, 3.0 equiv.), paraformaldehyde (451 mg, 15.0 mmol, 3.0 equiv.) and $NaBH_4$ (568 mg, 15.0 mmol, 3.0 equiv.) in MeOH (12.5 mL). Purification by flash column chromatography (EtOAc/pentane, *v/v* = 1:9) led to isolation of the title compound as a brown oil (815 mg, 3.50 mmol, 70%).

^{1}H NMR (300 MHz, $CDCl_3$, 300 K): δ_H (ppm) = 7.46 – 7.42 (m, 2H), 6.43 – 6.38 (m, 2H), 3.97 (s, 1H), 2.81 (s, 3H).

^{13}C NMR (75 MHz, $CDCl_3$, 300 K): δ_C (ppm) = 148.8 (C_q), 137.7 (*C*H), 114.6 (*C*H), 77.8 (C_q), 30.6 (CH_3).

HRMS (ESI) *m/z* = 233.9774 calcd. for $[C_7H_8NIH]^+$ $[M+H]^+$, found: 233.9773.

The analytical data are consistent with those reported in literature.[7]

4-(Methylamino)benzonitrile (1n):

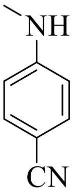

The title compound was prepared following the general procedure **GP2** using 4-aminobenzonitrile (591 mg, 5.00 mmol, 1.0 equiv.), NaOMe (810 mg, 15.0 mmol, 3.0 equiv.), paraformaldehyde (451 mg, 15.0 mmol, 3.0 equiv.) and $NaBH_4$ (568 mg, 15.0 mmol, 3.0 equiv.) in MeOH (12.5 mL). Purification by flash column chromatography (EtOAc/pentane, *v/v* = 3:7) led to isolation of the title compound as a colourless solid (555 mg, 4.20 mmol, 84%).

^{1}H NMR (300 MHz, $CDCl_3$, 300 K): δ_H (ppm) = 7.45 – 7.41 (m, 2H), 6.58 – 6.54 (m, 2H), 4.33 (s, 1H), 2.88 (d, *J* = 5.0 Hz, 3H).

^{13}C NMR (75 MHz, $CDCl_3$, 300 K): δ_C (ppm) = 152.2 (C_q), 133.7 (*C*H), 120.6 (C_q), 111.8 (*C*H), 98.4 (C_q), 30.0 (CH_3).

HRMS (ESI) *m/z* = 133.0760 calcd. for $[C_8H_8N_2H]^+$ $[M+H]^+$, found: 133.0760.

The analytical data are consistent with those reported in literature.[8]

Methyl 4-(methylamino)benzoate (1p):

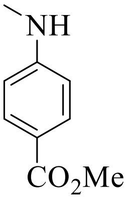

The title compound was prepared following the general procedure **GP2** using methyl 4-aminobenzoate (756 mg, 5.00 mmol, 1.0 equiv.), NaOMe (810 mg, 15.0 mmol, 3.0 equiv.), paraformaldehyde (451 mg, 15.0 mmol, 3.0 equiv.) and $NaBH_4$ (568 mg, 15.0 mmol, 3.0 equiv.) in MeOH (12.5 mL). Purification by flash column chromatography (EtOAc/pentane, *v/v* = 1:4) led to isolation of the title compound as a colourless solid (653 mg, 3.95 mmol, 79%).

^{1}H NMR (300 MHz, $CDCl_3$, 300 K): δ_H (ppm) = 7.90 – 7.85 (m, 2H), 6.57 – 6.52 (m, 2H), 4.31 (s, 1H), 3.87 (s, 3H), 2.87 (s, 3H).

^{13}C NMR (75 MHz, $CDCl_3$, 300 K): δ_C (ppm) = 167.5 (C_q), 152.9 (C_q), 131.5 (*C*H), 118.1 (C_q), 111.1 (*C*H), 51.5 (CH_3), 30.1(CH_3).

HRMS (ESI) *m/z* = 188.0682 calcd. for $[C_9H_{11}NO_2Na]^+$ $[M+Na]^+$, found: 188.0679.

The analytical data are consistent with those reported in literature.[8]

4-Methoxy-*N*-methylaniline (1q):

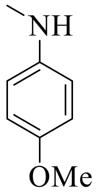

The title compound was prepared following the general procedure **GP2** using 4-methoxyaniline (616 mg, 5.00 mmol, 1.0 equiv.), NaOMe (810 mg, 15.0 mmol, 3.0 equiv.), paraformaldehyde (451 mg, 15.0 mmol, 3.0 equiv.) and $NaBH_4$ (568 mg, 15.0 mmol, 3.0 equiv.) in MeOH (12.5 mL). Purification by flash column chromatography (EtOAc/pentane, *v/v* = 1:4) led to isolation of the title compound as a colourless solid (594 mg, 4.33 mmol, 87%).

^{1}H NMR (300 MHz, $CDCl_3$, 300 K): δ_H (ppm) = 6.88 – 6.82 (m, 2H), 6.65 – 6.60 (m, 2H), 3.79 (s, 3H), 3.47 (s, 1H), 2.83 (s, 3H).

^{13}C NMR (75 MHz, $CDCl_3$, 300 K): δ_C (ppm) = 152.1 (C_q), 143.8 (C_q), 114.9 (*C*H), 113.7 (*C*H), 55.9 (CH_3), 31.6 (CH_3).

HRMS (ESI) *m/z* = 138.0913 calcd. for $[C_8H_{11}NOH]^+$ $[M+H]^+$, found: 138.0910.

The analytical data are consistent with those reported in literature.[9]

3.2 Synthesis of Ketenes

Ethylphenylketene (2a):

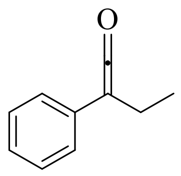

Ethylphenylketene **2a** was prepared following general procedure **GP3** using 2-phenylbutanoic acid (3.28 g, 20.0 mmol, 1.0 equiv.), oxalyl chloride (3.43 mL, 40.0 mmol, 2.0 equiv.) and NEt_3 (11.1 mL, 80.0 mmol, 4.0 equiv.) in CH_2Cl_2 (35 mL) and THF (30 mL). Purification by short-path vacuum distillation (high-vacuum, 70 °C) gave the title compound as a deep yellow oil (2.45 g, 16.7 mmol, 84%) which can be stored in the fridge for months.

^{1}H NMR (300 MHz, $CDCl_3$, 300 K): δ_H (ppm) = 7.55 – 7.50 (m, 2H), 7.31 – 7.24 (m, 3H), 2.65 (q, J = 7.4 Hz, 2H), 1.45 (t, J = 7.4 Hz, 3H).

^{13}C NMR (75 MHz, $CDCl_3$, 300 K): δ_C (ppm) = 205.4 (C_q), 132.9 (C_q), 129.0 (*C*H), 124.2 (*C*H), 124.0 (*C*H), 41.9 (C_q), 17.0 (CH_2), 12.9 (CH_3).

The analytical data are consistent with those reported in the literature.[10]

Methylphenylketene (2b):

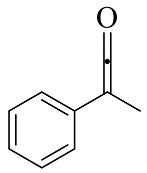

Methylphenylketene **2b** was prepared following general procedure **GP3** using 2-phenylpropanoic acid (1.37 mL, 10.0 mmol, 1.0 equiv.), oxalyl chloride (1.29 mL, 15.0 mmol, 1.5 equiv.) and NEt_3 (5.55 mL, 40.0 mmol, 4.0 equiv.) in CH_2Cl_2 (10 mL) and Et_2O (15 mL). Purification by short-path vacuum distillation (70 °C, 0.47 mbar) gave the title compound as a deep yellow oil (768 mg, 5.81 mmol, 58%).

^{1}H NMR (300 MHz, $CDCl_3$, 300 K): δ_H (ppm) = 7.23 – 7.17 (m, 2H), 6.99 – 6.88 (m, 3H), 1.87 (s, 3H).

^{13}C NMR (75 MHz, $CDCl_3$, 300 K): δ_C (ppm) = 205.6 (C_q), 133.4 (C_q), 129.0 (*C*H), 124.2 (*C*H), 123.7 (*C*H), 123.6 (*C*H), 33.8 (C_q), 8.6 (CH_3).

The analytical data are consistent with those reported in the literature.[11]

2-(4-Bromophenyl)but-1-en-1-one (2c):

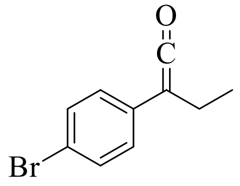

Following a modified procedure from the literature[12], diisopropylamine (8.43 mL, 60.0 mmol, 3.0 equiv.) was dissolved in anhydrous THF (25 mL). *n*-BuLi (1.6 M in hexanes, 31.3 mL, 50.0 mmol, 2.5 equiv.) was added slowly at 0 °C and the mixture was stirred at room temperature for 30 minutes. After cooling to 0 °C, a solution of 4-bromophenylacetic acid (4.30 g, 20.0 mmol,

1.0 equiv.) in THF (20) was added slowly. The suspension was stirred for one hour at 0 °C, then ethyliodide (2.41 mL, 30.0 mmol, 1.5 equiv.) was added slowly. The reaction mixture was allowed to reach room temperature overnight and then adjusted to pH 1 by addition of hydrochloric acid (2 N aqueous solution, 7 mL). EtOAc (50 mL) was added and the phases were separated. The aqueous phase was extracted with EtOAc (3 x 50 mL), the combined organic phases were washed with brine (100 mL), dried over $MgSO_4$ and concentrated *in vacuo*. Purification by RP-MPLC (MeOH/water, gradient from 5% to 90%) gave 2-(4-bromophenyl)butanoic acid as a colourless solid (3.09 g, 12.7 mmol, 64%). The acid (3.10 g, 12.7 mmol, 1.0 equiv.) was then transformed to the desired ketene following general procedure **GP2** using oxalyl chloride (1.64 mL, 19.1 mmol, 1.5 equiv.) and NEt_3 (7.06 mL, 50.9 mmol, 4.0 equiv.) in CH_2Cl_2 (20 mL) and Et_2O (20 mL). Purification by Kugelrohr distillation (100 °C, 0.28 mbar) gave the title compound as a slightly yellow oil (982 mg, 4.36 mmol, 34%) which has been used immediately after preparation.

^{1}H NMR (300 MHz, $CDCl_3$, 300 K): δ_H (ppm) = 7.30 – 7.25 (m, 2H), 6.78 – 6.73 (m, 2H), 2.27 (q, J = 7.4 Hz, 2H), 1.09 (t, J = 7.4 Hz, 3H).

^{13}C NMR (75 MHz, $CDCl_3$, 300 K): δ_C (ppm) = 204.3 (C_q), 132.0 (C_q and CH)), 125.5 (CH), 117.2 (C_q), 41.8 (C_q), 17.0 (CH$_2$), 12.8 (CH$_3$).

The analytical data are consistent with those reported in the literature.[11]

2-(4-Iodophenyl)but-1-en-1-one (2d):

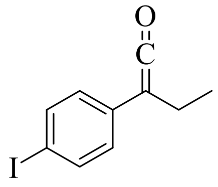

Following a modified procedure from the literature[12], diisopropylamine (6.32 mL, 45.0 mmol, 3.0 equiv.) was dissolved in anhydrous THF (20 mL). *n*-BuLi (1.6 M in hexanes, 23.4 mL, 37.5 mmol, 2.5 equiv.) was added slowly at 0 °C and the mixture was stirred at room temperature for 30 minutes. After cooling to 0 °C, a solution of 4-iodophenylacetic acid (3.93 g, 15.0 mmol, 1.0 equiv.) in THF (15) was added slowly. The suspension was stirred for one hour at 0 °C, then ethyliodide (1.81 mL, 22.5 mmol, 1.5 equiv.) was added slowly. The reaction mixture was allowed to reach room temperature overnight and then adjusted to pH 1 by addition of hydrochloric acid (2 N aqueous solution, 5 mL). EtOAc (50 mL) was added and the phases were separated. The aqueous phase was extracted with EtOAc (3 x 50 mL), the combined organic phases were washed with brine (100 mL), dried over $MgSO_4$ and concentrated *in vacuo*. Purification by RP-MPLC (MeOH/water, gradient from 5% to 90%) gave 2-(4-iodophenyl)butanoic acid as a colourless solid (2.07 g, 7.03 mmol, 47%). The acid (2.03 g,

7.00 mmol, 1.0 equiv.) was then transformed to the desired ketene following general procedure **GP2** using oxalyl chloride (952 µL, 10.5 mmol, 1.5 equiv.) and NEt_3 (3.88 mL, 28.0 mmol, 4.0 equiv.) in CH_2Cl_2 (20 mL) and Et_2O (20 mL). Purification by Kugelrohr distillation (125 °C, 0.40 mbar) gave the title compound as a slightly yellow oil (331 mg, 1.22 mmol, 17%) which has been used immediately after preparation.

^{1}H NMR (300 MHz, $CDCl_3$, 300 K): δ_H (ppm) = 7.28 (d, J = 8.2 Hz, 2H), 6.46 (d, J = 8.2 Hz, 2H), 2.08 (q, J = 7.4 Hz, 2H), 0.91 (t, J = 7.4 Hz, 3H).

^{13}C NMR (75 MHz, $CDCl_3$, 300 K): δ_C (ppm) = 204.1 (C_q), 137.8 (CH), 132.7 (C_q), 125.8 (CH), 87.8 (C_q), 41.9 (C_q), 16.9 (CH_2), 12.8 (CH_3).

2-(4-Isobutylphenyl)prop-1-en-1-one (2e):

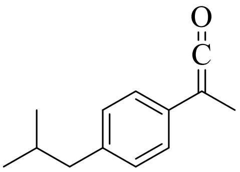

Ibuprofeneketene **2e** was prepared following general procedure **GP3** using ibuprofene (2.06 g, 10.0 mmol, 1.0 equiv.), oxalyl chloride (1.72 mL, 20.0 mmol, 2.0 equiv.) and NEt_3 (5.54 mL, 40.0 mmol, 4.0 equiv.) in CH_2Cl_2 (15 mL) and Et_2O (15 mL). Purification by short-path vacuum distillation (150 °C, 0.28 mbar) gave the title compound as a deep yellow oil (862 mg, 4.58 mmol, 46%).

^{1}H NMR (300 MHz, $CDCl_3$, 300 K): δ_H (ppm) = 7.18 – 7.14 (m, 2H), 7.01 – 6.97 (m, 2H), 2.50 (d, J = 7.2 Hz, 2H), 2.04 (s, 3H), 1.97 – 1.81 (m, 1H), 0.96 (d, J = 6.6 Hz, 6H).

^{13}C NMR (75 MHz, $CDCl_3$, 300 K): δ_C (ppm) = 206.8 (C_q), 137.7 (C_q), 130.3 (C_q), 129.8 (CH), 123.4 (CH), 45.0 (CH_2), 33.4 (C_q), 30.4 (CH), 22.4 (CH_3), 8.7 (CH_3).

The analytical data are consistent with those reported in the literature.[13]

Isopropylphenylketene (2f):

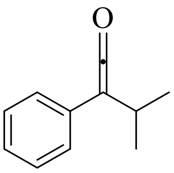

Isopropylphenylketene **2f** was prepared following general procedure **GP3**using 3-methyl-2-phenylbutanoic acid (1.78 g, 10.0 mmol, 1.0 equiv.), oxalyl chloride (1.29 mL, 15.0 mmol, 1.5 equiv.) and NEt_3 (5.55 mL, 40.0 mmol, 4.0 equiv.) in CH_2Cl_2 (10 mL) and Et_2O (15 mL). Purification by Kugelrohr distillation (100 °C, 0.30 mbar) gave the title compound as an inseperable 11.5:1-mixture with residual acid chloride (NMR yield **2f**: 861 mg, 5.38 mmol, 54%).[1] This mixture was used for the oxindole synthesis without further purification and analysis.

[1] Determined by ^{1}H-NMR spectroscopy. Ketene **2f** was identified by comparison with spectra from the literature.[11].

Diphenylketene (2g):

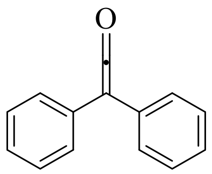

Diphenylketene **2ig** was prepared following general procedure **GP3** using 2,2-diphenylacetic acid (2.12 g, 10.0 mmol, 1.0 equiv.), oxalyl chloride (1.72 mL, 20.0 mmol, 2.0 equiv.) and NEt_3 (5.54 mL, 40.0 mmol, 4.0 equiv.) in CH_2Cl_2 (15 mL) and Et_2O (15 mL). Purification by short-path vacuum distillation (150 °C, 0.24 mbar) gave the title compound as a deep yellow oil (1.34 g, 6.88 mmmol, 69%).

^{1}H NMR (300 MHz, $CDCl_3$, 300 K): δ_H (ppm) = 7.34 – 7.28 (m, 4H), 7.19 – 7.13 (m, 6H).

^{13}C NMR (75 MHz, $CDCl_3$, 300 K): δ_C (ppm) = 200.0 (C_q), 129.7 (C_q), 128.2 (*C*H), 126.6 (*C*H), 125.1 (*C*H), 45.8 (C_q).

The analytical data are consistent with those reported in the literature.[14]

Cycloheptylidenemethanone (2h):

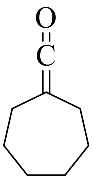

Ketene **2h** was prepared following general procedure **GP3** using Cycloheptanecarboxylic acid (2.40 mL, 20.0 mmol, 1.0 equiv.), Oxalyl chloride (3.41 mL, 40.0 mmol, 2.0 equiv.) and NEt_3 (11.1 mL, 80.0 mmol, 4.0 equiv.) in CH_2Cl_2 (20 mL) and Et_2O (25 mL). Purification by short-path vacuum distillation (70 °C, 0.8 mbar) gave the title compound as a yellow oil (412 mg, 3.32 mmol, 17%) which has been used immediately after distillation due to rapid decomposition.

^{1}H NMR (300 MHz, $CDCl_3$, 300 K): δ_H (ppm) = 2.25 – 2.21 (m, 4H), 1.70 – 1.61 (m, 4H), 1.59 – 1.51 (m, 4H).

The analytical data are consistent with those reported in the literature.[14]

3.3 Synthesis of Oxindoles

3-Ethyl-1-methyl-3-phenylindolin-2-one (3aa)

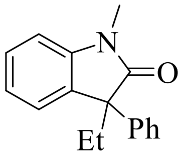

The title compound was prepared following the general procedure **GP4** using *N*-methylaniline **1a** (22 µL, 0.20 mmol, 1.0 equiv.), EtMgBr (3.0 M solution in Et_2O, 73 µL, 0.22 mmol, 1.1 equiv.), ketene **2a** (39 µL, 0.30 mmol, 1.5 equiv.) and iodine (112 mg, 0.440 mmol, 2.2 equiv.) in THF (20 mL). Purification by RP-MPLC (MeOH/H_2O, gradient from 5% to 90%) gave the title compound as a colourless solid (41.2 mg, 0.164 mmol, 82%).

^{1}H NMR (300 MHz, $CDCl_3$, 300 K): δ_H (ppm) = 7.30 – 7.11 (m, 7H), 7.06 – 7.01 (m, 1H), 6.84 – 6.81 (m, 1H), 3.14 (s, 3H), 2.41 – 2.29 (m, 1H), 2.21 – 2.09 (m, 1H), 0.60 (t, *J* = 7.4 Hz, 3H).

^{13}C NMR (75 MHz, $CDCl_3$, 300 K): δ_C (ppm) = 178.6 (C_q), 144.1 (C_q), 140.2 (C_q), 132.1 (C_q), 128.5 (*C*H), 128.1 (*C*H), 127.2 (*C*H), 127.0 (*C*H), 124.8 (*C*H), 122.6 (*C*H), 108.2 (*C*H), 57.3 (C_q), 30.9 (CH_2), 26.4 (CH_3), 9.1 (CH_3).

HRMS (ESI) *m/z* = 274.1202 calcd. for $[C_{17}H_{17}NONa]^+$ $[M+Na]^+$, found: 274.1203.

The analytical data are consistent with those reported in literature.[15]

1,3-Diethyl-3-phenylindolin-2-one (3ab)

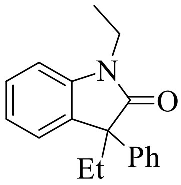

The title compound was prepared following the general procedure **GP4** using *N*-ethylaniline **1b** (25 µL, 0.20 mmol, 1.0 equiv.), EtMgBr (3.0 M solution in Et_2O, 73 µL, 0.22 mmol, 1.1 equiv.), ketene **2a** (39 µL, 0.30 mmol, 1.5 equiv.) and iodine (112 mg, 0.440 mmol, 2.2 equiv.) in THF (20 mL). Purification by RP-MPLC (MeOH/H_2O, gradient from 5% to 90%) gave the title compound as a colourless oil (26.0 mg, 98.0 µmol, 49%).

^{1}H NMR (300 MHz, $CDCl_3$, 300 K): δ_H (ppm) = 7.38 – 7.21 (m, 7H), 7.13 – 7.08 (m, 1H), 6.95 – 6.92 (m, 1H), 3.91 – 3.69 (m, 2H), 2.46 (dq, *J* = 13.3, 7.4 Hz, 1H), 2.24 (dq, *J* = 13.3, 7.4 Hz, 1H), 1.28 (t, *J* = 7.2 Hz, 3H), 0.69 (t, *J* = 7.3 Hz, 3H).

^{13}C NMR (75 MHz, $CDCl_3$, 300 K): δ_C (ppm) = 178.2 (C_q), 143.2 (C_q), 140.5 (C_q), 132.5 (C_q), 128.5 (*C*H), 128.0 (*C*H), 127.1 (*C*H), 126.9 (*C*H), 124.9 (*C*H), 122.4 (*C*H), 108.3 (*C*H), 57.1 (C_q), 34.8 (CH_2), 30.8 (CH_2), 12.7 (CH_3), 8.9 (CH_3).

HRMS (ESI) *m/z* = 288.1359 calcd. for $[C_{18}H_{19}NONa]^+$ $[M+Na]^+$, found: 288.1358.

The analytical data are consistent to those reported in the literature.[16]

3-Ethyl-1-isopropyl-3-phenylindolin-2-one (3ac)

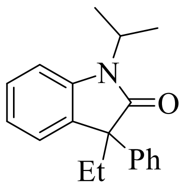

The title compound was prepared following the general procedure **GP4** using *N*-isopropylaniline **1c** (29 µL, 0.20 mmol, 1.0 equiv.), EtMgBr (3.0 M solution in Et_2O, 73 µL, 0.22 mmol, 1.1 equiv.), ketene **2a** (39 µL, 0.30 mmol, 1.5 equiv.) and iodine (112 mg, 0.440 mmol, 2.2 equiv.) in THF (20 mL). Purification by RP-MPLC (MeOH/H_2O, gradient from 5% to 90%) gave the title compound as a colourless oil (19.6 mg, 70.0 µmol, 35%).

FT-IR (neat): ν (cm^{-1}) = 3062, 2970, 2935, 2878, 1708, 1608, 1484, 1465, 1399, 1352, 1311, 1296, 1244, 1211, 1137, 1080, 1027, 918, 749, 726, 696, 625.

^{1}H NMR (300 MHz, $CDCl_3$, 300 K): δ_H (ppm) = 7.36 – 7.07 (m, 9H), 4.70 (hept, *J* = 7.1 Hz, 1H), 2.54 – 2.42 (m, 1H), 2.28 – 2.17 (m, 1H), 1.52 (d, *J* = 7.1 Hz, 6H), 0.68 (t, *J* = 7.3 Hz, 3H).

^{13}C NMR (75 MHz, $CDCl_3$, 300 K): δ_C (ppm) = 178.3 (C_q), 142.8 (C_q), 140.7 (C_q), 132.9 (C_q), 128.5 (*C*H), 127.7 (*C*H), 127.1 (*C*H), 126.8 (*C*H), 124.8 (*C*H), 122.1 (*C*H), 109.9 (*C*H), 56.9 (C_q), 43.9 (*C*H), 30.7 (CH_2), 19.5 (2 x CH_3), 8.8 (CH_3).

HRMS (ESI) *m/z* = 302.1521 calcd. for $[C_{19}H_{21}NONa]^+$ $[M+Na]^+$, found: 302.1514.

1-Cyclohexyl-3-ethyl-3-phenylindolin-2-one (3ad)

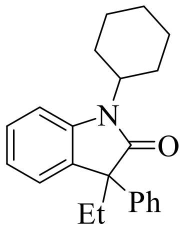

The title compound was prepared following the general procedure **GP4** using *N*-cyclohexylaniline **1d** (35 µL, 0.20 mmol, 1.0 equiv.), EtMgBr (3.0 M solution in Et_2O, 73 µL, 0.22 mmol, 1.1 equiv.), ketene **2a** (39 µL, 0.30 mmol, 1.5 equiv.) and iodine (112 mg, 0.440 mmol, 2.2 equiv.) in THF (20 mL). Purification by RP-MPLC (MeOH/H_2O, gradient from 5% to 90%) gave the title compound as a colourless oil (31.4 mg, 98.0 µmol, 49%).

FT-IR (neat): ν (cm^{-1}) = 3053, 2934, 2858, 2009, 1706, 1609, 1483, 1484, 1369, 1346, 1306, 1256, 1236, 1202, 1112, 1080, 1034, 907, 749, 728, 696, 672, 648, 611.

^{1}H NMR (600 MHz, $CDCl_3$, 300 K): δ_H (ppm) = 7.34 – 7.32 (m, 2H), 7.30 – 7.27 (m, 3H), 7.24 – 7.21 (m, 1H), 7.17 (dd, *J* = 7.4, 1.4 Hz, 1H), 7.13 (d, *J* = 7.9 Hz, 1H), 7.08 – 7.05 (m, 1H), 4.23 – 4.18 (m, 1H), 2.50 – 2.43 (m, 1H), 2.26 – 2.16 (m, 2H), 1.91 – 1.88 (m, 2H), 1.76 – 1.75 (m, 3H), 1.48 – 1.36 (m, 2H), 1.31 – 1.22 (m, 2H), 0.67 (t, *J* = 7.3 Hz, 3H).

^{13}C NMR (151 MHz, $CDCl_3$, 300 K): δ_C (ppm) = 178.4 (C_q), 143.2 (C_q), 140.9 (C_q), 132.9 (C_q), 128.5 (*C*H), 127.7 (*C*H), 127.1 (*C*H), 126.8 (*C*H), 124.8 (*C*H), 122.0 (*C*H), 110.0 (*C*H), 56.8 (C_q), 52.3 (*C*H), 30.7 (CH_2), 29.3 (CH_2), 29.2 (CH_2), 26.0 (2 x CH_2), 25.4 (CH_2), 8.8 (CH_3).

HRMS (ESI) *m/z* = 342.1834 calcd. for $[C_{22}H_{25}NONa]^+$ $[M+Na]^+$, found: 342.1827.

3-Ethyl-1,3-diphenylindolin-2-one (3ae)

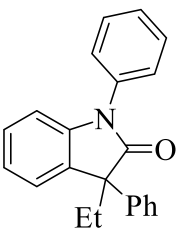

The title compound was prepared following the general procedure **GP4** using diphenylamin **1e** (33.8 mg, 0.200 mmol, 1.0 equiv.), EtMgBr (3.0 M-solution in Et_2O, 73 µL, 0.22 mmol, 1.1 equiv.), ketene **2a** (39 µL, 0.30 mmol, 1.5 equiv.) and iodine (112 mg, 0.440 mmol, 2.2 equiv.) in THF (20 mL). Purification by RP-MPLC ($MeOH/H_2O$, gradient from 5% to 90%) gave the title compound as a colourless solid (34.9 mg, 0.111 mmol, 56%).

MP: 117-119 °C.

FT-IR (neat): ν (cm^{-1}) = 3058, 2965, 2925, 2876, 1715, 1608, 1583, 1496, 1480, 1463, 1333, 1319, 1280, 1207, 1173, 1108, 913, 748, 723, 694, 640, 608.

^{1}H NMR (300 MHz, $CDCl_3$, 300 K): δ_H (ppm) = 7.53 – 7.11 (m, 13H), 6.90 – 6.87 (m, 1H), 2.62 – 2.50 (m, 1H), 2.37 – 2.25 (m, 1H), 0.81 (t, *J* = 7.3 Hz, 3H).

^{13}C NMR (75 MHz, $CDCl_3$, 300 K): δ_C (ppm) = 178.1 (C_q), 144.1 (C_q), 140.4 (C_q), 134.6 (C_q), 132.0 (C_q), 129.6 (*C*H), 128.6 (*C*H), 128.0 (2 x *C*H), 127.3 (*C*H), 127.0 (*C*H), 126.7 (*C*H), 125.0 (*C*H), 123.1 (*C*H), 109.5 (*C*H), 57.4 (C_q), 31.3 (CH_2), 9.1 (CH_3).

HRMS (ESI) *m/z* = 336.1359 calcd. for $[C_{22}H_{19}NONa]^+$ $[M+Na]^+$, found: 336.1359.

1-Benzyl-3-ethyl-3-phenylindolin-2-one (3af)

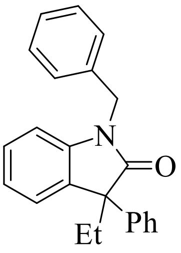

The title compound was prepared following the general procedure **GP4** using *N*-benzylaniline **1f** (36.7 mg, 0.200 mmol, 1.0 equiv.), EtMgBr (3.0 M solution in Et_2O, 73 µL, 0.22 mmol, 1.1 equiv.), ketene **2a** (39 µL, 0.30 mmol, 1.5 equiv.) and iodine (112 mg, 0.440 mmol, 2.2 equiv.) in THF (20 mL). Purification by RP-MPLC ($MeOH/H_2O$, gradient from 5% to 90%) gave the title compound as a colourless oil (35.1 mg, 0.107 mmol, 54%).

^{1}H NMR (300 MHz, $CDCl_3$, 300 K): δ_H (ppm) = 7.39 – 7.17 (m, 12H), 7.09 – 7.04 (m, 1H), 6.80 – 6.77 (m, 1H), 4.92 (dd, *J* = 15.7, 6.9 Hz, 2H), 2.51 (dq, *J* = 13.4, 7.3 Hz, 1H), 2.28 (dq, *J* = 13.4, 7.3 Hz, 1H), 0.73 (t, *J* = 7.3 Hz, 3H).

^{13}C NMR (75 MHz, $CDCl_3$, 300 K): δ_C (ppm) = 178.7 (C_q), 143.3 (C_q), 140.4 (C_q), 136.1 (C_q), 132.2 (C_q), 128.8 (*C*H), 128.6 (*C*H), 128.0 (*C*H), 127.6 (*C*H), 127.4 (*C*H), 127.3 (*C*H), 127.0 (*C*H), 124.8 (*C*H), 122.6 (*C*H), 109.3 (*C*H), 57.3 (C_q), 44.0 (CH_2), 30.8 (CH_2), 9.2 (CH_3).

HRMS (ESI) *m/z* = 350.1521 calcd. for $[C_{23}H_{21}NONa]^+$ $[M+Na]^+$, found: 350.1513.

The analytical data are consistent with those reported in literature.[17]

3-Ethyl-3-phenyl-1-((*R*)-1-phenylethyl)indolin-2-one (3ag)

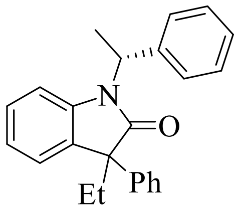

The title compound was prepared following the general procedure **GP4** using (*R*)-*N*-(1-phenylethyl)aniline **1g** (39.5 mg, 0.200 mmol, 1.0 equiv.), EtMgBr (3.0 M solution in Et_2O, 73 µL, 0.22 mmol, 1.1 equiv.), ketene **2a** (39 µL, 0.30 mmol, 1.5 equiv.) and iodine (112 mg, 0.440 mmol, 2.2 equiv.) in THF (20 mL). Deprotonation time and stirring time after ketene addition were extended to one hour each. Purification by RP-MPLC (MeOH/H_2O, gradient from 5% to 90%) and preparative HPLC (MeCN/H_2O, gradient from 5% to 70%) gave the title compound as a colourless oil (49.7 mg, 0.146 mmol, 73%, 1.9:1 d.r.[2]).

FT-IR (neat): ν (cm^{-1}) = 3059, 2967, 2923, 1704, 1607, 1483, 1465, 1347, 1197, 1077, 1026, 909, 748, 727, 695, 670, 633.

^{1}H NMR (600 MHz, $CDCl_3$, 300 K): δ_H (ppm) = 7.39 – 7.25 (m, 18H, both diastereoisomers), 7.19 – 7.17 (m, 2H, both diastereoisomers), 7.09 – 7.01 (m, 4H, both diastereoisomers), 6.58 – 6.54 (m, 2H, both diastereoisomers), 5.94 – 5.86 (m, 2H, both diastereoisomers), 2.58 – 2.52 (m, 2H, both diastereoisomers), 2.34 – 2.25 (m, 2H, both diastereoisomers), 1.84 (d, *J* = 7.2 Hz, 3H, minor diastereoisomer), 1.82 (d, *J* = 7.2 Hz, 3H, major diastereoisomer), 0.81 (t, *J* = 7.3 Hz, 3H, major diastereoisomer), 0.72 (t, *J* = 7.3 Hz, 3H, minor diastereoisomer).

^{13}C NMR (151 MHz, $CDCl_3$, 300 K): δ_C (ppm) = 178.6 (C_q), 142.0 (2 x C_q), 140.8 (C_q), 140.7 (C_q), 139.3 (C_q), 132.7 (2 x C_q), 128.6 (3 x *C*H), 127.5 (*C*H), 127.3 (2 x *C*H), 127.2 (2 x *C*H), 126.9 (*C*H), 126.7 (*C*H), 126.6 (*C*H), 124.7 (*C*H), 124.6 (*C*H), 122.3 (*C*H), 122.2 (*C*H), 111.0 (2 x *C*H), 57.0 (C_q), 49.0 (2 x *C*H), 30.7 (CH_2), 30.5 (CH_2), 16.3 (2 x CH_3), 9.3 (CH_3), 9.0 (CH_3).

HRMS (ESI) *m/z* = 364.1677 calcd. for $[C_{24}H_{23}NONa]^+$ $[M+Na]^+$, found: 364.1668.

[2] Determined by ^{1}H-NMR spectroscopy of the crude product prior to purification.

3-Ethyl-1-((*R*)-1-(naphthalen-2-yl)ethyl)-3-phenylindolin-2-one (3ah)

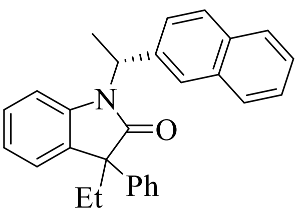

The title compound was prepared following the general procedure **GP4** using (*R*)-*N*-(1-(naphthalen-2-yl)ethyl)aniline **1h** (49.5 mg, 0.200 mmol, 1.0 equiv.), EtMgBr (3.0 M solution in Et_2O, 73 µL, 0.22 mmol, 1.1 equiv.), ketene **2a** (39 µL, 0.30 mmol, 1.5 equiv.) and iodine (112 mg, 0.440 mmol, 2.2 equiv.) in THF (20 mL). Deprotonation time and stirring time after ketene addition were extended to one hour each. Purification by RP-MPLC (MeOH/H_2O, gradient from 5% to 90%) and preparative HPLC (MeCN/H_2O, gradient from 5% to 70%) gave the title compound as a colourless oil (61.9 mg, 0.158 mmol, 79%, 1.5:1 d.r.[3]).

FT-IR (neat): ν (cm^{-1}) = 3059, 2968, 2924, 1708, 1607, 1483, 1465, 1197, 1077, 1027, 913, 749, 696, 634.

^{1}H NMR (600 MHz, $CDCl_3$, 300 K): δ_H (ppm) = 7.38 – 7.28 (m, 18H, both diastereoisomers), 7.25 – 7.24 (m, 2H, both diastereoisomers), 7.27 – 7.16 (m, 2H, both diastereoisomers), 7.08 – 7.00 (m, 4H, both diastereoisomers), 6.57 – 6.54 (m, 2H, both diastereoisomers), 5.92 – 5.86 (m, 2H, both diastereoisomers), 2.57 – 2.54 (m, 2H, both diastereoisomers), 2.33 – 2.25 (m, 2H, both diastereoisomers), 1.84 (d, *J* = 7.2 Hz, 3, minor diastereoisomer), 1.81 (d, *J* = 7.2 Hz, 3H, major diastereoisomer), 0.80 (t, *J* = 7.3 Hz, 3H, major diastereoisomer), 0.72 (t, *J* = 7.3 Hz, 3H, minor diastereoisomer).

^{13}C NMR (151 MHz, $CDCl_3$, 300 K): δ_C (ppm) = 178.6 (*C*q), 142.0 (2 x *C*q), 140.8 (*C*q), 140.7 (*C*q), 139.3 (*C*q), 132.7 (*C*q), 128.6 (2 x *C*H), 128.5 (*C*H), 127.5 (*C*H), 127.3 (2 x *C*H), 127.2 (2 x *C*H), 126.9 (*C*H), 126.7 (*C*H), 126.6 (*C*H), 124.7 (*C*H), 124.6 (*C*H) , 122.2 (2 x *C*H), 111.0 (*C*H), 110.9 (*C*H), 57.0 (*C*q), 56.9 (*C*q), 49.0 (2 x *C*H), 30.7 (CH_2), 30.5 (CH_2), 16.3 (2 x CH_3), 9.3 (CH_3), 9.0 (CH_3).[4]

HRMS (ESI) *m/z* = 414.1834 calcd. for $[C_{28}H_{25}NONa]^+$ $[M+Na]^+$, found: 414.1825.

[3] Determined by ^{1}H-NMR spectroscopy of the crude product prior to purification.
[4] Some aromatic signals of the diastereoisomers are superimposed and therefore not resolved.

3-Ethyl-1,5-dimethyl-3-phenylindolin-2-one (3ai)

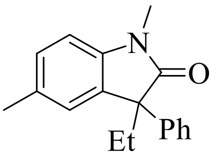

The title compound was prepared following the general procedure **GP4** using *N*,4-dimethylaniline **1i** (25 µL, 0.20 mmol, 1.0 equiv.), EtMgBr (3.0 M solution in Et_2O, 73 µL, 0.22 mmol, 1.1 equiv.), ketene **2a** (39 µL, 0.30 mmol, 1.5 equiv.) and iodine (112 mg, 0.440 mmol, 2.2 equiv.) in THF (20 mL). Purification by RP-MPLC (MeOH/H_2O, gradient from 20% to 90%) to give the title compound as a colourless oil (40.5 mg, 0.153 mmol, 76%).

FT-IR (neat): ν (cm^{-1}) = 3022, 2966, 2933, 2876, 2359, 1704, 1618, 1601, 1497, 1457, 1323, 1348, 1262, 1146, 1113, 1070, 806, 774, 695, 638.

^{1}H NMR (300 MHz, $CDCl_3$, 300 K): δ_H (ppm) = 7.40 – 7.22 (m, 5H), 7.16 – 7.13 (m, 1H), 7.06 – 7.05 (m, 1H), 6.82 (d, *J* = 7.9 Hz, 1H), 3.24 (s, 3H), 2.52 – 2.39 (m, 4H), 2.30 – 2.18 (m, 1H), 0.71 (t, *J* = 7.3 Hz, 3H).

^{13}C NMR (75 MHz, $CDCl_3$, 300 K): δ_C (ppm) = 178.6 (C_q), 141.8 (C_q), 140.5 (C_q), 132.2 (C_q), 132.1 (C_q), 128.5 (*C*H), 128.4 (*C*H), 127.1 (*C*H), 127.0 (*C*H), 125.5 (*C*H), 107.9 (*C*H), 57.4 (C_q), 30.7 (CH_2), 26.3 (CH_3), 21.3 (CH_3), 9.1 (CH_3).

HRMS (ESI) *m/z* = 288.1364 calcd. for $[C_{18}H_{19}NONa]^+$ $[M+Na]^+$, found: 288.1355.

3-Ethyl-5-fluoro-1-methyl-3-phenylindolin-2-one (3aj)

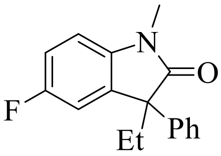

The title compound was prepared following the general procedure **GP4** using 4-fluoro-*N*-methylaniline **1j** (24 µL, 0.20 mmol, 1.0 equiv.), EtMgBr (3.0 M-solution in Et_2O, 73 µL, 0.22 mmol, 1.1 equiv.), ketene **2a** (39 µL, 0.30 mmol, 1.5 equiv.) and iodine (112 mg, 0.440 mmol, 2.2 equiv.) in THF (20 mL). Purification by RP-MPLC (MeOH/H_2O, gradient from 20% to 90%) to give the title compound as a colourless oil (38.7 mg, 0.144 mmol, 72%).

FT-IR (neat): ν (cm^{-1}) = 3060, 2968, 2934, 2878, 1710, 1618, 1493, 1452, 1350, 1262, 1112, 856, 811, 741, 723, 697, 561.

^{1}H NMR (600 MHz, $CDCl_3$, 300 K): δ_H (ppm) = 7.36 – 7.29 (m, 4H), 7.27 – 7.24 (m, 1H), 7.05 (dd, *J* = 8.5, 2.6 Hz, 1H), 6.98 (d, *J* = 2.6 Hz, 1H), 6.84 (d, *J* = 8.5 Hz, 1H), 3.23 (s, 3H), 2.45 (dq, *J* = 13.4, 7.3 Hz, 1H), 2.22 (dq, *J* = 13.4, 7.3 Hz, 1H), 0.70 (t, *J* = 7.3 Hz, 3H).

$^{13}C\{^1H,^{19}F\}$ NMR (151 MHz, $CDCl_3$, 300 K): δ_C (ppm) = 178.3 (C_q), 159.3 (C_q), 140.1 (C_q), 139.6 (C_q), 133.8 (C_q), 128.6 (*C*H), 127.4 (*C*H), 126.8 (*C*H), 114.4 (*C*H), 112.8 (*C*H), 108.5 (*C*H), 57.8 (C_q), 30.7 (CH_2), 26.4 (CH_3), 8.9 (CH_3).

$^{19}F\{^1H,^{13}C\}$ NMR (282 MHz, $CDCl_3$, 300 K): δ_F (ppm) = −120.4.

HRMS (ESI) *m/z* = 292.1114 calcd. for $[C_{17}H_{16}NOFNa]^+$ $[M+Na]^+$, found: 292.1106.

5-Chloro-3-ethyl-1-methyl-3-phenylindolin-2-one (3ak)

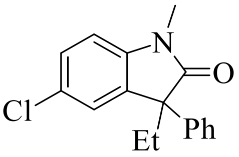

The title compound was prepared following the general procedure **GP4** using 4-chloro-*N*-methylaniline **1k** (24 µL, 0.20 mmol, 1.0 equiv.), EtMgBr (3.0 M-solution in Et_2O, 73 µL, 0.22 mmol, 1.1 equiv.), ketene **2a** (39 µL, 0.30 mmol, 1.5 equiv.) and iodine (112 mg, 0.440 mmol, 2.2 equiv.) in THF (20 mL). Purification by RP-MPLC (MeOH/H_2O, gradient from 20% to 90%) to give the title compound as a colourless oil (30.3 mg, 0.106 mmol, 53%).

^1H NMR (300 MHz, $CDCl_3$, 300 K): δ_H (ppm) = 7.37 – 7.21 (m, 7H), 6.85 (d, *J* = 8.3 Hz, 1H), 3.24 (s, 3H), 2.45 (dq, *J* = 13.4, 7.3 Hz, 1H), 2.24 (dq, *J* = 13.4, 7.3 Hz, 1H), 0.71 (t, *J* = 7.3 Hz, 3H).

^{13}C NMR (75 MHz, $CDCl_3$, 300 K): δ_C (ppm) = 178.1 (C_q), 142.7 (C_q), 139.6 (C_q), 134.0 (C_q), 128.7 (*C*H), 128.1 (*C*H), 128.0 (C_q), 127.5 (*C*H), 126.8 (*C*H), 125.1 (*C*H), 109.1 (*C*H), 57.6 (C_q), 30.7 (CH_2), 26.4 (CH_3), 9.0 (CH_3).

HRMS (ESI) *m/z* = 308.0813 calcd. for $[C_{17}H_{16}NOClNa]^+$ $[M+Na]^+$, found: 308.0811.

The analytical data are consistent with those reported in the literature.[15]

5-Bromo-3-ethyl-1-methyl-3-phenylindolin-2-one (3al)

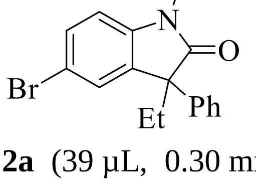

The title compound was prepared following the general procedure **GP4** using 4-bromo-*N*-methylaniline **1l** (37.2 mg, 0.200 mmol, 1.0 equiv.), EtMgBr (3.0 M-solution in Et_2O, 73 µL, 0.22 mmol, 1.1 equiv.), ketene **2a** (39 µL, 0.30 mmol, 1.5 equiv.) and iodine (112 mg, 0.440 mmol, 2.2 equiv.) in THF (20 mL). Purification by RP-MPLC (MeOH/H_2O, gradient from 20% to 90%) to give the title compound as a colourless oil (45.3 mg, 0.137 mmol, 69%).

^1H NMR (300 MHz, $CDCl_3$, 300 K): δ_H (ppm) = 7.42 – 7.39 (m, 1H), 7.28 – 7.18 (m, 5H), 6.75 – 6.72 (m, 1H), 3.16 (d, 3H), 2.44 – 2.32 (m, 1H), 2.22 – 2.10 (m, 1H), 0.64 (t, *J* = 7.3 Hz, 3H).

^{13}C NMR (75 MHz, $CDCl_3$, 300 K): δ_C (ppm) = 178.0 (C_q), 143.2 (C_q), 139.5 (C_q), 134.4 (C_q), 131.0 (*C*H), 128.7 (*C*H), 127.8 (*C*H), 127.5 (*C*H), 126.8 (*C*H), 115.4 (C_q), 109.6 (*C*H), 57.6 (C_q), 30.7 (CH_2), 26.4 (CH_3), 9.0 (CH_3).

HRMS (ESI) *m/z* = 352.0308 calcd. for $[C_{17}H_{16}NOBrNa]^+$ $[M+Na]^+$, found: 352.0309.

The analytical data are consistent with those reported in the literature.[15]

5-Iodo-3-ethyl-1-methyl-3-phenylindolin-2-one (3am)

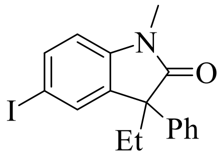

The title compound was prepared following the general procedure **GP4** using 4-iodo-*N*-methylaniline **1m** (46.6 mg, 0.200 mmol, 1.0 equiv.), EtMgBr (3.0 M-solution in Et_2O, 73 µL, 0.22 mmol, 1.1 equiv.), ketene **2a** (39 µL, 0.30 mmol, 1.5 equiv.) and iodine (112 mg, 0.440 mmol, 2.2 equiv.) in THF (20 mL). Purification by RP-MPLC (MeOH/H_2O, gradient from 20% to 90%) to give the title compound as a colourless oil (65.6 mg, 0.174 mmol, 87%).

FT-IR (neat): ν (cm^{-1}) = 3058, 2966, 2932, 2875, 1710, 1601, 1484, 1457, 1361, 1341, 1320, 1111, 1077, 807, 726, 696, 627.

^{1}H NMR (300 MHz, $CDCl_3$, 300 K): δ_H (ppm) = 7.68 – 7.64 (m, 1H), 7.50 (d, *J* = 1.7 Hz, 1H), 7.33 – 7.22 (m, 5H), 6.70 (d, *J* = 8.1 Hz, 1H), 3.22 (s, 3H), 2.49 – 2.38 (m, 1H), 2.28 – 2.16 (m, 1H), 0.70 (t, *J* = 7.1 Hz, 3H).

^{13}C NMR (75 MHz, $CDCl_3$, 300 K): δ_C (ppm) = 177.9 (C_q), 143.9 (C_q), 139.6 (C_q), 137.0 (*C*H), 134.7 (C_q), 133.3 (*C*H), 128.7 (*C*H), 127.5 (*C*H), 126.8 (*C*H), 110.2 (*C*H), 85.2 (C_q), 57.4 (C_q), 30.7 (CH_2), 26.4 (CH_3), 9.0 (CH_3).

HRMS (ESI) *m/z* = 400.0169 calcd. for $[C_{17}H_{16}NOINa]^+$ $[M+Na]^+$, found: 400.0167.

3-Ethyl-1-methyl-2-oxo-3-phenylindoline-5-carbonitrile (3an)

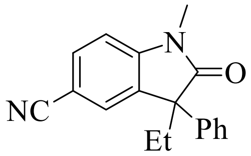

The title compound was prepared following the general procedure **GP4** using 4-cyano-*N*-methylaniline **1n** (26.4 mg, 0.200 mmol, 1.0 equiv.), EtMgBr (3.0 M-solution in Et_2O, 73 µL, 0.22 mmol, 1.1 equiv.), ketene **2a** (39 µL, 0.30 mmol, 1.5 equiv.) and iodine (112 mg, 0.440 mmol, 2.2 equiv.) in THF (20 mL). Purification by RP-MPLC (MeOH/H_2O, gradient from 20% to 90%) to give the title compound as a colourless oil (50.8 mg, 0.184 mmol, 92%).

FT-IR (neat): ν (cm^{-1}) = 2972, 2252, 2224, 1718, 1613, 1496, 1343, 1259, 1069, 904, 821, 695, 647, 610.

^{1}H NMR (300 MHz, $CDCl_3$, 300 K): δ_H (ppm) = 7.71 – 7.67 (m, 1H), 7.50 (d, *J* = 1.1 Hz, 1H), 7.37 – 7.26 (m, 5H), 7.02 – 6.99 (m, 1H), 3.28 (s, 3H), 2.53 – 2.34 (m, 1H), 2.31 – 2.22 (m, 1H), 0.71 (t, *J* = 7.3 Hz, 3H).

^{13}C NMR (75 MHz, $CDCl_3$, 300 K): δ_C (ppm) = 178.3 (C_q), 147.9 (C_q), 138.7 (C_q), 133.5 (*C*H), 133.3 (C_q), 128.8 (*C*H), 128.0 (*C*H), 127.8 (*C*H), 126.7 (*C*H), 119.2 (C_q), 108.7 (*C*H), 105.8 (C_q), 57.1 (C_q), 30.8 (CH_2), 26.6 (CH_3), 9.0 (CH_3).

HRMS (ESI) *m/z* = 299.1155 calcd. for $[C_{18}H_{16}N_2ONa]^+$ $[M+Na]^+$, found: 299.1157.

5-Acetyl-3-ethyl-1-methyl-3-phenylindolin-2-one (3ao)

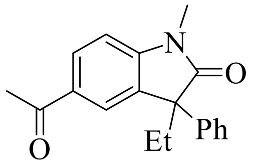

The title compound was prepared following the general procedure **GP4** using 1-(4-(methylamino)phenyl)ethan-1-one **1o** (29.8 mg, 0.200 mmol, 1.0 equiv.), EtMgBr (3.0 M-solution in Et_2O, 73 µL, 0.22 mmol, 1.1 equiv.), ketene **2a** (39 µL, 0.30 mmol, 1.5 equiv.) and iodine (112 mg, 0.440 mmol, 2.2 equiv.) in THF (20 mL). Purification by RP-MPLC (MeOH/H_2O, gradient from 20% to 90%) to give the title compound as a colourless oil (17.6 mg, 0.060 mmol, 30%).

FT-IR (neat): ν (cm^{-1}) = 2968, 1719, 1674, 1607, 1497, 1441, 1372, 1346, 1270, 1221, 1056, 747, 698, 614.

^{1}H NMR (600 MHz, $CDCl_3$, 300 K): δ_H (ppm) = 8.02 (dd, *J* = 8.2, 1.8 Hz, 1H), 7.88 (dd, *J* = 1.8, 0.5 Hz, 1H), 7.37 – 7.24 (m, 5H), 6.97 (d, *J* = 8.2 Hz, 1H), 3.29 (s, 3H), 2.61 (s, 3H), 2.47 (dq, *J* = 13.4, 7.3 Hz, 1H), 2.30 (dq, *J* = 13.4, 7.3 Hz, 1H), 0.70 (t, *J* = 7.3 Hz, 3H).

^{13}C NMR (151 MHz, $CDCl_3$, 300 K): δ_C (ppm) = 196.9 (C_q), 178.9 (C_q), 148.4 (C_q), 139.5 (C_q), 132.5 (C_q), 132.2 (C_q), 130.2 (*C*H), 128.7 (*C*H), 127.5 (*C*H), 126.8 (*C*H), 124.6 (*C*H), 107.6 (*C*H), 57.2 (C_q), 30.8 (CH_2), 26.6 (CH_3), 26.4 (CH_3), 9.0 (CH_3).

HRMS (ESI) *m/z* = 316.1308 calcd. for $[C_{19}H_{19}NO_2Na]^+$ $[M+Na]^+$, found: 316.1310.

Methyl 3-ethyl-1-methyl-2-oxo-3-phenylindoline-5-carboxylate (3ap)

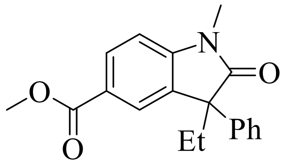

The title compound was prepared following the general procedure **GP4** using methyl 4-(methylamino)benzoate **1p** (33.0 mg, 0.200 mmol, 1.0 equiv.), EtMgBr (3.0 M-solution in Et_2O, 73 µL, 0.22 mmol, 1.1 equiv.), ketene **2a** (39 µL, 0.30 mmol, 1.5 equiv.) and iodine (112 mg, 0.440 mmol, 2.2 equiv.) in THF (20 mL). Purification by RP-MPLC (MeOH/H_2O,

gradient from 5% to 60%) to give the title compound as a colourless oil (51.0 mg, 0.165 mmol, 82%).

FT-IR (neat): ν (cm^{-1}) = 2950, 2878, 1709, 1613, 1498, 1442, 1345, 1273, 1224, 1103, 1064, 913, 768, 728, 631.

^{1}H NMR (300 MHz, $CDCl_3$, 300 K): δ_H (ppm) = 8.12 – 8.09 (m, 1H), 7.92 (d, *J* = 1.7 Hz, 1H), 7.38 – 7.25 (m, 5H), 6.96 (d, *J* = 8.2 Hz, 1H), 3.91 (s, 3H), 3.27 (s, 3H), 2.52 – 2.41 (m, 1H), 2.35 – 2.23 (m, 1H), 0.69 (t, *J* = 7.3 Hz, 3H).

^{13}C NMR (75 MHz, $CDCl_3$, 300 K): δ_C (ppm) = 178.9 (C_q), 167.0 (C_q), 148.2 (C_q), 139.5 (C_q), 132.1 (C_q), 130.9 (*C*H), 128.7 (*C*H), 127.5 (*C*H), 126.9 (*C*H), 126.0 (*C*H), 124.6 (C_q), 107.8 (*C*H), 57.2 (C_q), 52.1 (CH_3), 30.9 (CH_2), 26.5 (CH_3), 9.0 (CH_3).

HRMS (ESI) *m/z* = 332.1257 calcd. for $[C_{19}H_{19}NO_3Na]^+$ $[M+Na]^+$, found: 332.1257.

3-Ethyl-5-methoxy-1-methyl-3-phenylindolin-2-one (3aq)

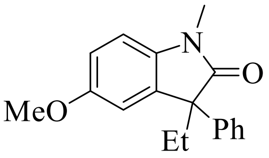

The title compound was prepared following the general procedure **GP4** using 4-(methoxy)-*N*-methylaniline **1q** (27.4 mg, 0.200 mmol, 1.0 equiv.), EtMgBr (3.0 M-solution in Et_2O, 73 µL, 0.22 mmol, 1.1 equiv.), ketene **2a** (39 µL, 0.30 mmol, 1.5 equiv.) and iodine (112 mg, 0.440 mmol, 2.2 equiv.) in THF (20 mL). Purification by RP-MPLC (MeOH/H_2O, gradient from 20% to 90%) to give the title compound as a colourless oil (40.0 mg, 0.142 mmol, 71%).

FT-IR (neat): ν (cm^{-1}) = 2935, 1706, 1600, 1497, 1459, 1288, 1041, 807, 698.

^{1}H NMR (300 MHz, $CDCl_3$, 300 K): δ_H (ppm) = 7.39 – 7.22 (m, 5H), 6.90 – 6.81 (m, 3H), 3.82 (m, 3H), 3.23 – 3.22 (m, 3H), 2.52 – 2.40 (m, 1H), 2.28 – 2.16 (m, 1H), 0.73 – 0.68 (m, 3H).

^{13}C NMR (75 MHz, $CDCl_3$, 300 K): δ_C (ppm) = 178.3 (C_q), 156.0 (C_q), 140.3 (C_q), 137.7 (C_q), 133.5 (C_q), 128.5 (*C*H), 127.2 (*C*H), 126.9 (*C*H), 112.3 (*C*H), 112.2 (*C*H), 108.4 (*C*H), 57.8 (C_q), 55.8 (CH_3), 30.7 (CH_2), 26.4 (CH_3), 9.0 (CH_3).

HRMS (ESI) *m/z* = 304.1308 calcd. for $[C_{18}H_{19}NO_2Na]^+$ $[M+Na]^+$, found: 304.1308.

3-Ethyl-1,4-dimethyl-3-phenylindolin-2-one (3ar) and 3-Ethyl-1,6-dimethyl-3-phenylindolin-2-one (3ar'):

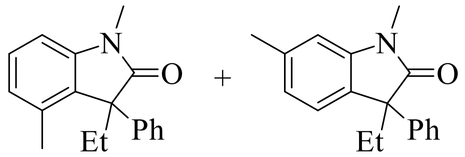

The title compound was prepared following the general procedure **GP4** using *N*,3-dimethylaniline **1r** (25 µL, 0.20 mmol, 1.0 equiv.), EtMgBr (3.0 M-solution in Et_2O, 73 µL, 0.22 mmol, 1.1 equiv.), ketene **2a** (39 µL, 0.30 mmol, 1.5 equiv.) and iodine (112 mg, 0.440 mmol, 2.2 equiv.) in THF (20 mL). Purification by RP-MPLC (MeOH/H_2O, gradient from 20% to 90%) to give the title compound as a colourless oil. (40.3 mg, 0.152 mmol, 76%, 1.8:1 r.r.). Purification by preparative HPLC (MeCN/H_2O, gradient from 5% to 50%) gave analytically pure samples of both regioisomers.

3-Ethyl-1,4-dimethyl-3-phenylindolin-2-one (major regioisomer, **3ar**):

FT-IR (neat): ν (cm^{-1}) = 2967, 2934, 1713, 1600, 1471, 1365, 1346, 1243, 1054, 959, 913, 771, 741, 697, 667, 596.

^{1}H NMR (300 MHz, $CDCl_3$, 300 K): δ_H (ppm) = 7.31 – 7.19 (m, 6H), 6.89 (d, *J* = 7.8 Hz, 1H), 6.78 (d, *J* = 7.7 Hz, 1H), 3.22 (s, 3H), 2.71 (dq, *J* = 13.3, 7.3 Hz, 1H), 2.34 (dq, *J* = 13.3, 7.3 Hz, 1H), 2.03 (s, 3H), 0.61 (t, *J* = 7.3 Hz, 3H).

^{13}C NMR (151 MHz, $CDCl_3$, 300 K): δ_C (ppm) = 178.6 (C_q), 144.6 (C_q), 139.6 (C_q), 135.1 (C_q), 129.7 (C_q), 128.5 (*C*H), 128.1 (*C*H), 127.2 (*C*H), 126.7 (*C*H), 125.0 (*C*H), 105.7 (*C*H), 57.8 (C_q), 27.1 (CH_2), 26.4 (CH_3), 18.2 (CH_3), 9.0 (CH_3).

HRMS (ESI) *m/z* = 288.1357 calcd. for $[C_{18}H_{19}NONa]^+$ $[M+Na]^+$, found: 288.1354.

3-Ethyl-1,6-dimethyl-3-phenylindolin-2-one (minor regioisomer, **3ar'**):

FT-IR (neat): ν (cm^{-1}) = 3025, 2968, 2934, 1707, 1619, 1496, 1469, 1456, 1372, 1315, 1276, 1257, 1075, 1038, 957, 910, 730, 697, 602.

^{1}H NMR (300 MHz, $CDCl_3$, 300 K): δ_H (ppm) = 7.40 – 7.36 (m, 2H), 7.32 – 7.20 (m, 3H), 7.12 (d, *J* = 7.5 Hz, 1H), 6.96 – 6.93 (m, 1H), 6.74 (s, 1H), 3.22 (s, 3H), 2.47 – 2.32 (m, 4H), 2.22 (dq, *J* = 13.4, 7.4 Hz, 1H), 0.69 (t, *J* = 7.4 Hz, 3H).

^{13}C NMR (151 MHz, $CDCl_3$, 300 K): δ_C (ppm) = 178.9 (C_q), 144.2 (C_q), 140.4 (C_q), 138.2 (C_q), 129.0 (C_q), 128.4 (*C*H), 127.1 (*C*H), 127.0 (*C*H), 124.5 (*C*H), 123.1 (*C*H), 109.1 (*C*H), 57.1 (C_q), 30.9 (CH_2), 26.3 (CH_3), 21.8 (CH_3), 9.1 (CH_3).

HRMS (ESI) *m/z* = 288.1357 calcd. for $[C_{18}H_{19}NONa]^+$ $[M+Na]^+$, found: 288.1359.

3-Ethyl-1-methyl-3-phenyl-1,3-dihydro-2H-pyrrolo[3,2-b]pyridin-2-one (3as)

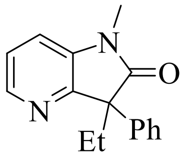

The title compound was prepared following the general procedure **GP4** using *N*-methylpyridin-3-amine **1s** (21 µL, 0.20 mmol, 1.0 equiv.), EtMgBr (3.0 M-solution in Et_2O, 73 µL, 0.22 mmol, 1.1 equiv.), ketene **2a** (39 µL, 0.30 mmol, 1.5 equiv.) and iodine (112 mg, 0.440 mmol, 2.2 equiv.) in THF (20 mL). Purification by RP-MPLC (MeOH/H_2O, gradient from 20% to 90%) to give the title compound as a colourless oil (19.7 mg, 0.078 mmol, 39%).

^{1}H NMR (300 MHz, $CDCl_3$, 300 K): δ_H (ppm) = 8.27 – 8.25 (m, 1H), 7.52 – 7.49 (m, 1H), 7.39 – 7.29 (m, 5H), 7.06 – 7.02 (m, 1H), 3.33 (s, 3H), 2.45 – 2.21 (m, 2H), 0.74 (t, *J* = 7.4 Hz, 3H).

^{13}C NMR (75 MHz, $CDCl_3$, 300 K): δ_C (ppm) = 178.2 (C_q), 157.5 (C_q), 147.1 (*C*H), 138.9 (C_q), 132.1 (*C*H), 128.7 (*C*H), 127.6 (*C*H), 126.8 (*C*H), 126.6 (C_q), 118.1 (*C*H), 57.0 (C_q), 30.9 (CH_2), 25.5 (CH_3), 9.1 (CH_3).

HRMS (ESI) *m/z* = 275.1160 calcd. for $[C_{16}H_{16}N_2ONa]^+$ $[M+Na]^+$, found: 275.1153.

The analytical data are consistent with those reported in the literature.[18]

1,3-Dimethyl-3-phenylindolin-2-one (3ba)

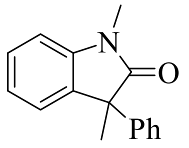

The title compound was prepared following the general procedure **GP4** using *N*-methylaniline **1a** (22 µL, 0.20 mmol, 1.0 equiv.), EtMgBr (3.0 M-solution in Et_2O, 73 µL, 0.22 mmol, 1.1 equiv.), ketene **2b** (39.6 mg, 0.300 mmol, 1.5 equiv.) and iodine (112 mg, 0.440 mmol, 2.2 equiv.) in THF (20 mL). Purification by RP-MPLC (MeOH/H_2O, gradient from 20% to 90%) to give the title compound as a colourless oil (27.6 mg, 0.116 mmol, 58%).

^{1}H NMR (300 MHz, $CDCl_3$, 300 K): δ_H (ppm) = 7.37 – 7.19 (m, 7H), 7.13 – 7.08 (m, 1H), 6.94 – 6.92 (m, 1H), 3.26 (s, 3H), 1.80 (s, 3H).

^{13}C NMR (75 MHz, $CDCl_3$, 300 K): δ_C (ppm) = 179.5 (C_q), 143.3 (C_q), 140.8 (C_q), 134.9 (C_q), 128.5 (*C*H), 128.1 (*C*H), 127.2 (*C*H), 126.7 (*C*H), 124.2 (*C*H), 122.8 (*C*H), 108.3 (*C*H), 52.2 (C_q), 26.5 (CH_3), 23.8 (CH_3).

HRMS (ESI) *m/z* = 260.1046 calcd. for $[C_{16}H_{15}NONa]^+$ $[M+Na]^+$, found: 260.1046.

The analytical data are consistent with those reported in the literature.[19]

3-(4-Bromophenyl)-3-ethyl-1-methylindolin-2-one (3ca)

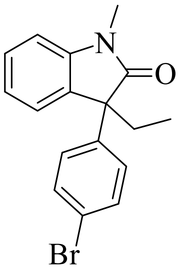

The title compound was prepared following the general procedure **GP4** using *N*-methylaniline **1a** (22 µL, 0.20 mmol, 1.0 equiv.), EtMgBr (3.0 M-solution in Et_2O, 73 µL, 0.22 mmol, 1.1 equiv.), ketene **2c** (67.5 mg, 0.300 mmol, 1.5 equiv.) and iodine (112 mg, 0.440 mmol, 2.2 equiv.) in THF (20 mL). Purification by RP-MPLC (MeOH/H_2O, gradient from 20% to 90%) to give the title compound as a colourless oil (46.1 mg, 0.140 mmol, 70%).

FT-IR (neat): ν (cm^{-1}) = 3055, 2966, 2932, 2877, 2247, 1705, 1609, 1489, 1469, 1370, 1347, 1254, 1098, 1074, 909, 814, 744, 731, 699, 570.

^{1}H NMR (300 MHz, $CDCl_3$, 300 K): δ_H (ppm) = 7.43 – 7.11 (m, 7H), 6.94 – 6.91 (m, 1H), 3.23 (s, 3H), 2.38 (dq, *J* = 13.3, 7.3 Hz, 1H), 2.20 (dq, *J* = 13.3, 7.3 Hz, 1H), 0.68 (t, *J* = 7.3 Hz, 3H).

^{13}C NMR (75 MHz, $CDCl_3$, 300 K): δ_C (ppm) = 178.1 (C_q), 144.1 (C_q), 139.3 (C_q), 131.5 (*C*H), 131.4 (C_q), 128.9 (*C*H), 128.4 (*C*H), 124.8 (*C*H), 122.7 (*C*H), 121.4 (C_q), 108.4 (*C*H), 56.9 (C_q), 31.0 (CH_2), 26.4 (CH_3), 9.0 (CH_3).

HRMS (ESI) *m/z* = 352.0309 calcd. for $[C_{17}H_{16}NOBrNa]^+$ $[M+Na]^+$, found: 352.0308.

3-(4-Iodophenyl)-3-ethyl-1-methylindolin-2-one (3da)

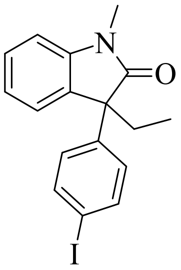

The title compound was prepared following the general procedure **GP4** using *N*-methylaniline **1a** (22 µL, 0.20 mmol, 1.0 equiv.), EtMgBr (3.0 M-solution in Et_2O, 73 µL, 0.22 mmol, 1.1 equiv.), ketene **2d** (81.6 mg, 0.300 mmol, 1.5 equiv.) and iodine (112 mg, 0.440 mmol, 2.2 equiv.) in THF (20 mL). Purification by RP-MPLC (MeOH/H_2O, gradient from 20% to 90%) to give the title compound as a colourless oil (57.1 mg, 0.151 mmol, 76%).

FT-IR (neat): ν (cm^{-1}) = 3054, 2966, 2932, 2876, 1709, 1610, 1486, 1469, 1371, 1348, 1255, 1090, 1075, 1005, 911, 813, 750, 698, 570.

^{1}H NMR (300 MHz, $CDCl_3$, 300 K): δ_H (ppm) = 7.64 – 7.59 (m, 2H), 7.39 – 7.33 (m, 1H), 7.23 – 7.10 (m, 4H), 6.94 – 6.91 (m, 1H), 3.23 (s, 3H), 2.44 – 2.32 (m, 1H), 2.25 – 2.13 (m, 1H), 0.68 (t, *J* = 7.3 Hz, 3H).

^{13}C NMR (75 MHz, $CDCl_3$, 300 K): δ_C (ppm) = 178.1 (C_q), 144.1 (C_q), 140.0 (C_q), 137.5 (*C*H), 131.3 (C_q), 129.1 (*C*H), 128.4 (*C*H), 124.7 (*C*H), 122.7 (*C*H), 108.4 (*C*H), 93.1 (C_q), 57.0 (C_q), 30.9 (CH_2), 26.4 (CH_3), 9.0 (CH_3).

HRMS (ESI) *m/z* = 400.0169 calcd. for $[C_{17}H_{xx}NOINa]^+$ $[M+Na]^+$, found: 400.0169.

3-(4-Isobutylphenyl)-1,3-dimethylindolin-2-one (3ea)

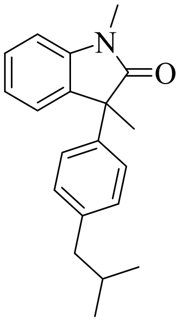

The title compound was prepared following the general procedure **GP4** using *N*-methylaniline **1a** (22 µL, 0.20 mmol, 1.0 equiv.), EtMgBr (3.0 M-solution in Et_2O, 73 µL, 0.22 mmol, 1.1 equiv.), ketene **2e** (56.5 mg, 0.300 mmol, 1.5 equiv.) and iodine (112 mg, 0.440 mmol, 2.2 equiv.) in THF (20 mL). Purification by RP-MPLC (MeOH/H_2O, gradient from 20% to 90%) to give the title compound as a colourless oil (23.6 mg, 80.4 µmol, 40%).

^{1}H NMR (300 MHz, $CDCl_3$, 300 K): δ_H (ppm) = 7.35 – 7.30 (m, 1H), 7.22 – 7.19 (m, 3H), 7.13 – 7.05 (m, 3H), 6.93 – 6.90 (m, 1H), 3.25 (s, 3H), 2.42 (d, *J* = 7.2 Hz, 2H), 1.87 – 1.78 (m, 4H), 0.89 – 0.87 (m, 6H).

^{13}C NMR (75 MHz, $CDCl_3$, 300 K): δ_C (ppm) = 179.7 (C_q), 143.3 (C_q), 140.7 (C_q), 138.0 (C_q), 135.0 (C_q), 129.3 (*C*H), 128.0 (*C*H), 126.3 (*C*H), 124.2 (*C*H), 122.7 (*C*H), 108.2 (*C*H), 51.9 (C_q), 45.0 (CH_2), 30.1 (*C*H), 26.5 (CH_3), 23.8 (CH_3), 22.4 (2 x CH_3).

HRMS (ESI) *m/z* = 316.1672 calcd. for $[C_{20}H_{23}NONa]^+$ $[M+Na]^+$, found: 316.1672.

The analalytical data are consistent with those reported in the literature.[20]

3-Isopropyl-1-methyl-3-phenylindolin-2-one (3fa) and *N*,3-Dimethyl-N,2-diphenylbutanamide (4fa)

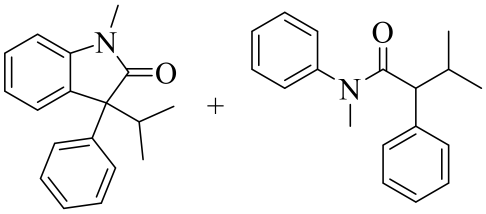

The title compounds were prepared following the general procedure **GP4** using *N*-methylaniline **1a** (22 µL, 0.20 mmol, 1.0 equiv.), EtMgBr (3.0 M-solution in Et_2O, 73 µL, 0.22 mmol, 1.1 equiv.), ketene **2f** (48.1 mg, 0.300 mmol, 1.5 equiv.) and iodine (112 mg, 0.440 mmol, 2.2 equiv.) in THF (20 mL). Purification by RP-MPLC (MeOH/H_2O, gradient from 5% to 60%) and preparative HPLC (MeCN/H_2O, gradient from 5% to 50%) to give the title compounds as a colourless oil and inseparable mixture (1:1.4 mixture, combined yield: 29.5 mg, 0.111 mmol, 56%).

^{1}H NMR (300 MHz, $CDCl_3$, 300 K): δ_H (ppm) = 7.49 – 6.90 (m, 10H from **4fa** and 9H from **3fa**), 3.24 (s, 3H from **4fa**), 3.20 (s, 3H from **3fa**), 3.01 (d, *J* = 10.5 Hz, 1H from **4fa**), 2.96 – 2.87 (m, 1H from **4fa**), 2.46 – 2.36 (m, 1H from **3fa**), 1.02 (d, *J* = 6.5 Hz, 3H from **4fa**), 0.96

(d, J = 7.0 Hz, 3H from **3fa**), 0.69 (d, J = 6.7 Hz, 3H from **3fa**), 0.50 (d, J = 6.7 Hz, 3H from **4fa**).

^{13}C NMR (75 MHz, $CDCl_3$, 300 K): δ_C (ppm) = 178.4 (C_q from **3fa**), 173.4 (C_q from **4fa**), 144.4 (C_q from **3fa**), 144.0 (C_q from **4fa**), 139.5 (C_q from **4fa**), 139.1 (C_q from **3fa**), 129.6, 129.5 (C_q from **3fa**), 128.7 (*C*H from **4fa**), 128.4 (*C*H from **4fa**), 128.3 (*C*H from **4fa**), 128.2 (*C*H from **3fa**), 127.9 (*C*H from **4fa**), 127.5 (*C*H from **4fa**), 127.1 (*C*H from **3fa**), 126.7 (*C*H from **4fa**), 126.2 (*C*H from **3fa**), 122.0 (*C*H from **3fa**), 108.2 (*C*H from **3fa**), 60.8 (C_q from **3fa**), 56.9 (*C*H from **4fa**), 37.6 (CH_3 from **4fa**), 36.1 (*C*H from **3fa**), 33.0 (*C*H from **4fa**), 26.2 (CH_3 from **3fa**), 21.9 (CH_3 from **4fa**), 20.2 (CH_3 from **4fa**), 17.6 (CH_3 from **3fa**), 17.5 (CH_3 from **3fa**).

HRMS (ESI) *m/z* = 288.1359 calcd. for $[C_{18}H_{19}NONa]^+$ [M(**3fa**)+Na]$^+$, found: 288.1360; 290.1515 calcd. for $[C_{18}H_{21}NONa]^+$ [M(**4fa**)+Na]$^+$, found: 290.1515.

The analytical data are consistent with those reported in the literature.[21]

1-Methyl-3,3-diphenylindolin-2-one (3ga)

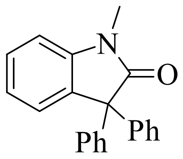

The title compound was prepared following the general procedure **GP4** using *N*-methylaniline **1a** (22 µL, 0.20 mmol, 1.0 equiv.), EtMgBr (3.0 M-solution in Et_2O, 73 µL, 0.22 mmol, 1.1 equiv.), ketene **2g** (58.3 mg, 0.300 mmol, 1.5 equiv.) and iodine (112 mg, 0.440 mmol, 2.2 equiv.) in THF (20 mL). Purification by RP-MPLC ($MeOH/H_2O$, gradient from 20% to 90%) to give the title compound as a colourless oil (33.2 mg, 0.111 mmol, 55%).

^{1}H NMR (600 MHz, $CDCl_3$, 300 K): δ_H (ppm) = 7.39 – 7.28 (m, 7H), 7.26 – 7.19 (m, 5H), 7.12 – 7.09 (m, 1H), 6.95 – 6.94 (m, 1H), 3.31 (s, 3H).

^{13}C NMR (151 MHz, $CDCl_3$, 300 K): δ_C (ppm) = 177.6 (C_q), 143.1 (C_q), 141.9 (C_q), 128.8 (C_q), 128.4 (2 x *C*H), 128.3 (*C*H), 127.3 (*C*H), 126.1 (*C*H), 122.8 (*C*H), 108.5 (*C*H), 26.7 (CH_3).[5]

HRMS (ESI) *m/z* = 322.1203 calcd. for $[C_{21}H_{17}NONa]^+$ [M+Na]$^+$, found: 322.1202.

The analytical data are consistent with those reported in the literature.[22]

---

[5] The signal of the quaternary α-C-atom is not resolved.

1'-Methylspiro[cycloheptane-1,3'-indolin]-2'-one (3ha)

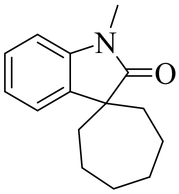

The title compound was prepared following the general procedure **GP4** using *N*-methylaniline **1a** (22 µL, 0.20 mmol, 1.0 equiv.), EtMgBr (3.0 M-solution in Et_2O, 73 µL, 0.22 mmol, 1.1 equiv.), ketene **2h** (37.3 mg, 0.300 mmol, 1.5 equiv.) and iodine (112 mg, 0.440 mmol, 2.2 equiv.) in THF (20 mL). Purification by RP-MPLC (MeOH/H_2O, gradient from 20% to 90%) to give the title compound as a colourless oil (11.9 mg, 51.9 µmol, 26%).

^{1}H NMR (300 MHz, $CDCl_3$, 300 K): δ_H (ppm) = 7.37 – 7.33 (m, 1H), 7.29 – 7.23 (m, 1H), 7.08 – 7.03 (m, 1H), 6.84 – 6.81 (m, 1H), 3.19 (s, 3H), 2.02 – 1.95 (m, 4H), 1.77 – 1.69 (m, 8H).

^{13}C NMR (75 MHz, $CDCl_3$, 300 K): δ_C (ppm) = 182.2 (C_q), 142.5 (C_q), 137.4 (C_q), 127.4 (*C*H), 122.7 (*C*H), 122.3 (*C*H), 107.8 (*C*H), 50.1 (C_q), 36.9 (CH_2), 31.3 (CH_2), 26.1 (CH_3), 23.8 (CH_2).

HRMS (ESI) *m/z* = 230.1539 calcd. for $[C_{15}H_{19}NOH]^+$ $[M+H]^+$, found: 230.1539.

The analytical data are consistent with those reported in the literature.[23]

3.4 Synthesis of Amides

N-Methyl-*N*,2-diphenylbutanamide (4aa)

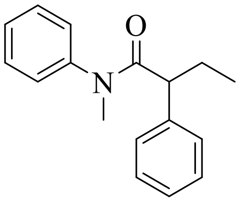

To a solution of *N*-methylaniline (22 µL, 0.20 mmol, 1.0 equiv.) in anhydrous THF (20 mL) was added EtMgBr (3.0 M-solution in THF, 73 µL, 0.22 mmol, 1.1 equiv.) at room temperature and the mixture was stirred for 30 minutes. Ketene **2a** (39 µL, 0.30 mmol, 1.5 equiv.) was added at room temperature and stirring was continued for further 30 minutes. Water (20 mL) and EtOAc (20 mL) were added and the phases were separated. The aqueous phase was extracted with EtOAc (3 x 20 mL), the combined organic phases were dried over $MgSO_4$ and concentrated *in vacuo*. Purification by RP-MPLC (MeOH/H_2O, gradient from 20% to 90%) to give the title compound as a colourless oil (46.1 mg, 0.182 mmol, 91%).

^{1}H NMR (300 MHz, $CDCl_3$, 300 K): δ_H (ppm) = 7.40 – 7.35 (m, 3H), 7.25 – 7.15 (m, 3H), 7.07 – 6.99 (m, 4H), 3.36 (dd, *J* = 8.4, 6.7 Hz, 1H), 3.25 (s, 3H), 2.17 – 2.03 (m, 1H), 1.74 – 1.60 (m, 1H), 0.80 (t, *J* = 7.3 Hz, 3H).

^{13}C NMR (75 MHz, $CDCl_3$, 300 K): δ_C (ppm) = 173.4 (C_q), 143.8 (C_q), 140.4 (C_q), 129.6 (*C*H), 128.3 (*C*H), 128.1 (2 x *C*H), 127.9 (*C*H), 126.7 (*C*H), 50.7 (*C*H), 37.6 (CH_2), 28.4 (CH_3), 12.4 (CH_3).

HRMS (ESI) *m/z* = 276.1359 calcd. for $[C_{17}H_{19}NONa]^+$ $[M+Na]^+$, found: 276.1359.

The analytical data are consistent with those reported in the literature.[24]

N-Methyl-*N*,2-diphenylbut-2-enamide (5aa)

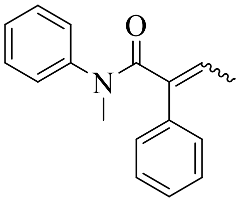

To a solution of *N*-methylaniline (22 µL, 0.20 mmol, 1.0 equiv.) in anhydrous THF (20 mL) was added EtMgBr (3.0 M-solution in THF, 73 µL, 0.22 mmol, 1.1 equiv.) at room temperature and the mixture was stirred for 30 minutes. Ketene **2a** (39 µL, 0.30 mmol, 1.5 equiv.) was added at room temperature and stirring was continued for further 30 minutes. Iodine (112 mg, 0.440 mmol, 2.2 equiv.) was added and the flask opened to air. The mixture was stirred at room temperature for 18 hours and then quenched by addition of water (20 mL) and EtOAc (20 mL). The phases were separated and the aqueous phase was extracted with EtOAc (3 x 20 mL). The combined organic phases were dried over $MgSO_4$ and concentrated *in vacuo*. Purification by RP-MPLC (MeOH/H_2O, gradient from 20% to 90%) followed by preparative HPLC (MeCN/H_2O, gradient from 5% to 40%) to give the title compound as a colourless oil (22.5 mg, 89.5 µmol, 45%).

^{1}H NMR (300 MHz, $CDCl_3$, 300 K): δ_H (ppm) = 7.20 – 7.10 (m, 8H), 6.88 – 6.85 (m, 2H), 5.74 (q, J = 7.0 Hz, 1H), 3.41 (s, 3H), 1.82 (d, J = 7.1 Hz, 3H).

^{13}C NMR (126 MHz, $CDCl_3$, 300 K): δ_C (ppm) = 169.8 (C_q), 143.2 (C_q), 139.4 (C_q), 137.8 (C_q), 128.6 (*C*H), 128.2 (*C*H), 127.1 (2 x *C*H), 126.4 (*C*H), 125.8 (*C*H), 125.6 (*C*H), 36.7 (*C*H_3), 16.0 (*C*H_3).

HRMS (ESI) *m/z* = 274.1202 calcd. for $[C_{15}H_{19}NONa]^+$ $[M+Na]^+$, found: 274.1202.

The analytical data are consistent with those reported in the literature.[25]

2-Chloro-*N*-methyl-*N*,2-diphenylacetamide (6)

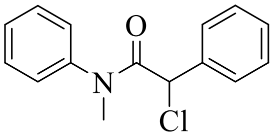

To a solution of *N*-methylaniline (1.08 mL, 10.0 mmol, 1.0 equiv.) in CH_2Cl_2 (50 mL) was added NEt_3 (2.77 mL, 20.0 mmol, 2.0 equiv.) at 0 °C. 2-Chloro-2-phenylacetyl chloride (1.90 mL, 12.0 mmol, 1.2 equiv.) was added dropwise over 5 minutes at this temperature and the mixture was allowed to warm to room temperature overnight. Water (50 mL) was added and the phases were separated. The aqueous phase was extracted with CH_2Cl_2 (3 x 50 mL). The combined organic phases were dried over $MgSO_4$, filtered and concentrated *in vacuo*. Purification by FC (EtOAc/pentane, *v/v* = 3:17) gave the title compound as a yellow oil (2.43 g, 9.34 mmol, 93%).

^{1}H NMR (300 MHz, $CDCl_3$, 300 K): δ_H (ppm) = 7.46 – 7.31 (m, 3H), 7.32– 7.27 (m, 5H), 7.13 (s, 2H), 5.33 (s, 1H), 3.31 (s, 3H).

^{13}C NMR (75 MHz, $CDCl_3$, 300 K): δ_C (ppm) = 167.5 (C_q), 142.7 (C_q), 136.5 (C_q), 130.1 (*C*H), 129.0 (*C*H), 128.7 (*C*H), 128.6 (*C*H), 128.3 (*C*H), 127.6 (*C*H), 56.9 (*C*H), 38.3 (*C*H_3).

HRMS (ESI) *m/z* = 282.0656 calcd. for $[C_{15}H_{14}NOClNa]^+$ $[M+Na]^+$, found: 282.0654.

The analytical data are consistent with those reported in the literature.[26]

2-Iodo-*N*-methyl-*N*,2-diphenylacetamide (7)

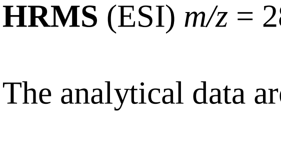

2-Chloro-*N*-methyl-*N*,2-diphenylacetamide **6** (100 mg, 0.385 mmol, 1.0 equiv.) was dissolved in anhydrous acetone (0.5 mL). Sodium iodide (69.3 mg, 0.462 mmol, 1.2 equiv.) was added at room temperature and mixture was stirred overnight. Brine (10 mL) and EtOAc (10 mL) were added and the phases separated. The aqueous phase was extracted with EtOAc (3 x 10 mL). The combined organic phases were dried over $MgSO_4$, filtered and concentrated *in vacuo*.

Purification by RP-MPLC (MeOH/H_2O, gradient from 5% to 90%) gave the title compound as a yellow oil (77.3 mg, 0.220 mmol, 57%).

FT-IR (neat): ν (cm^{-1}) = 3060, 2925, 1659, 1594, 1494, 1454, 1416, 1376, 1289, 1247, 1109, 1073, 1036, 1001, 912, 840, 773, 728, 695, 943, 557.

^{1}H NMR (300 MHz, $CDCl_3$, 300 K): δ_H (ppm) = 7.50 – 7.43 (m, 5H), 7.28 – 7.20 (m, 5H), 5.51 (s, 1H), 3.28 (s, 3H).

^{13}C NMR (75 MHz, $CDCl_3$, 300 K): δ_C (ppm) = 168.7 (C_q), 143.3 (C_q), 138.3 (C_q), 130.1 (*C*H), 128.9 (*C*H), 128.7 (*C*H), 128.5 (*C*H), 127.0 (*C*H), 38.5 (*C*H), 22.3 (CH_3).[6]

HRMS (ESI) *m/z* = 374.0012 calcd. for $[C_{15}H_{14}NOINa]^+$ $[M+Na]^+$, found: 374.0015.

[6] One aromatic *C*H-signal was not resolved.

4. NMR Data of compounds

^{1}H NMR (300 MHz, $CDCl_3$):

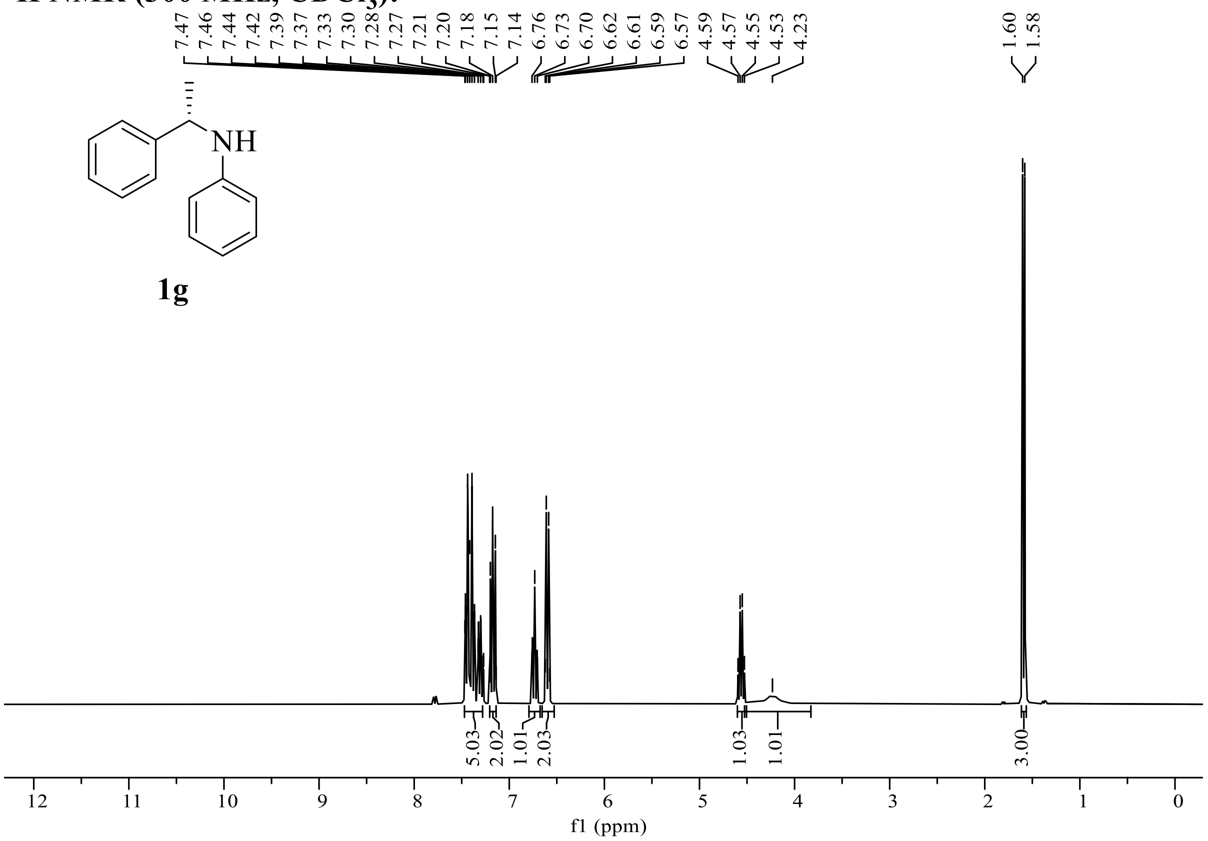

^{13}C NMR (75 MHz, $CDCl_3$):

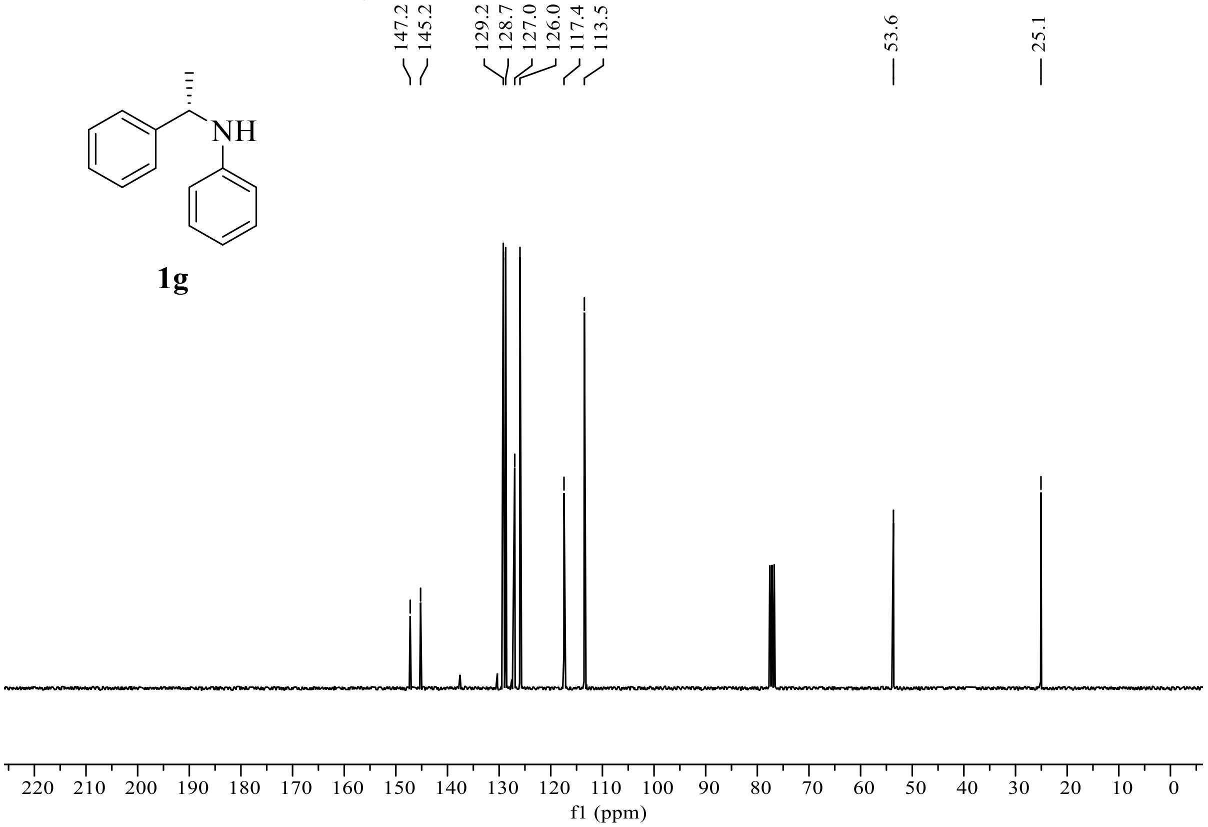

^{1}H NMR (300 MHz, $CDCl_3$):

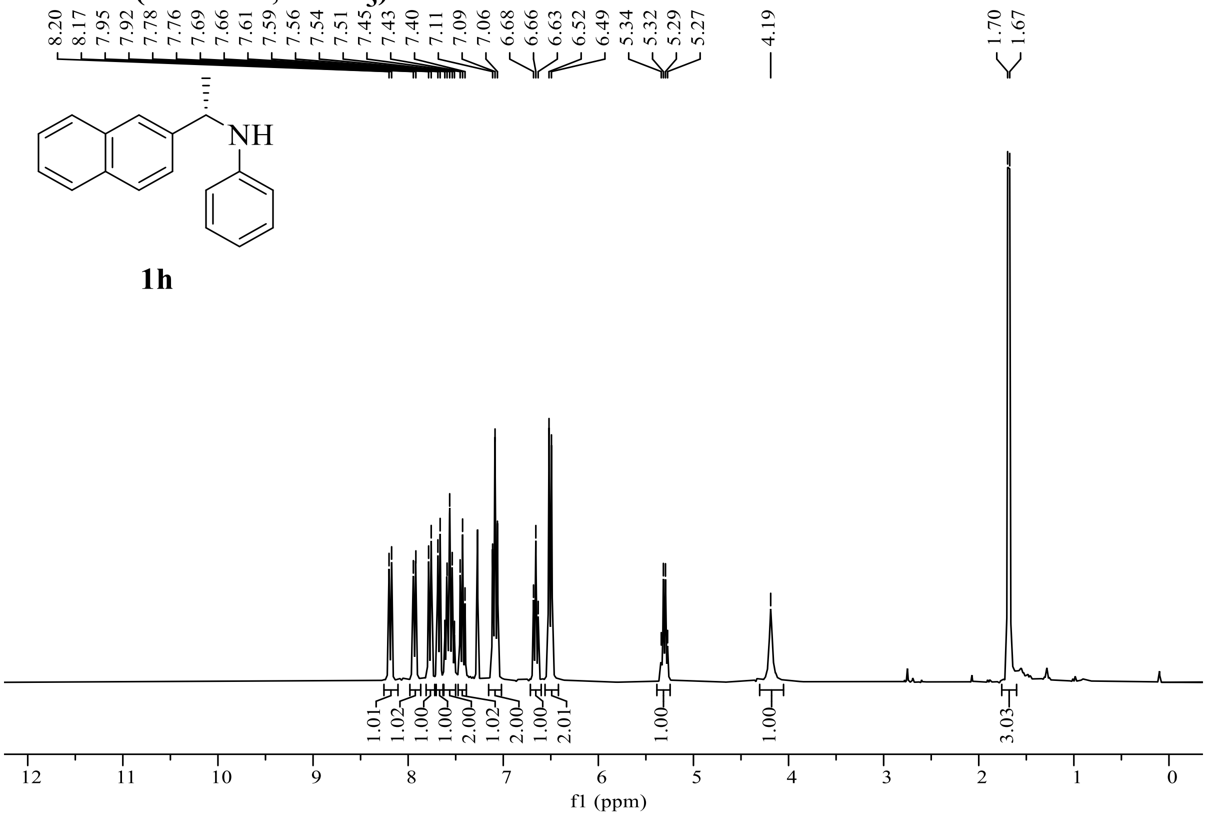

^{13}C NMR (75 MHz, $CDCl_3$):

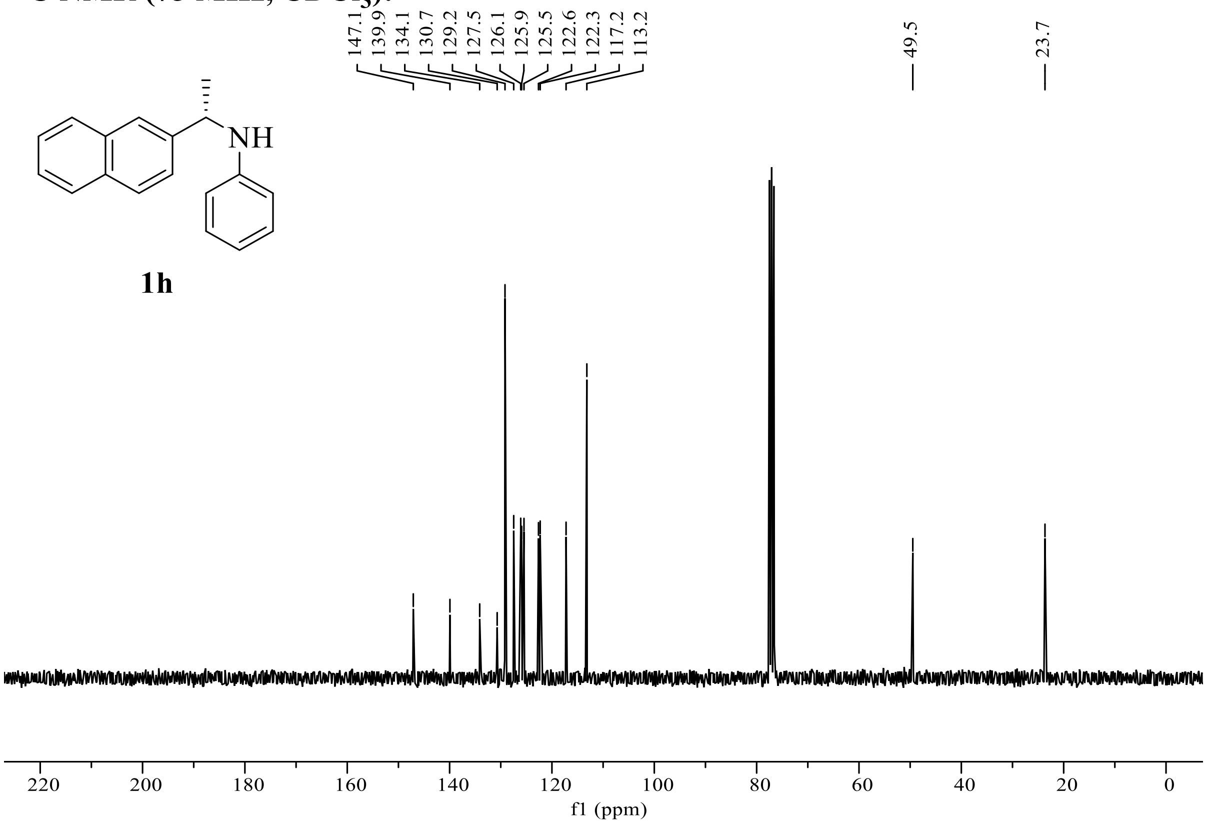

^{1}H NMR (300 MHz, $CDCl_3$):

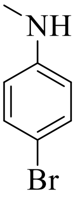

1l

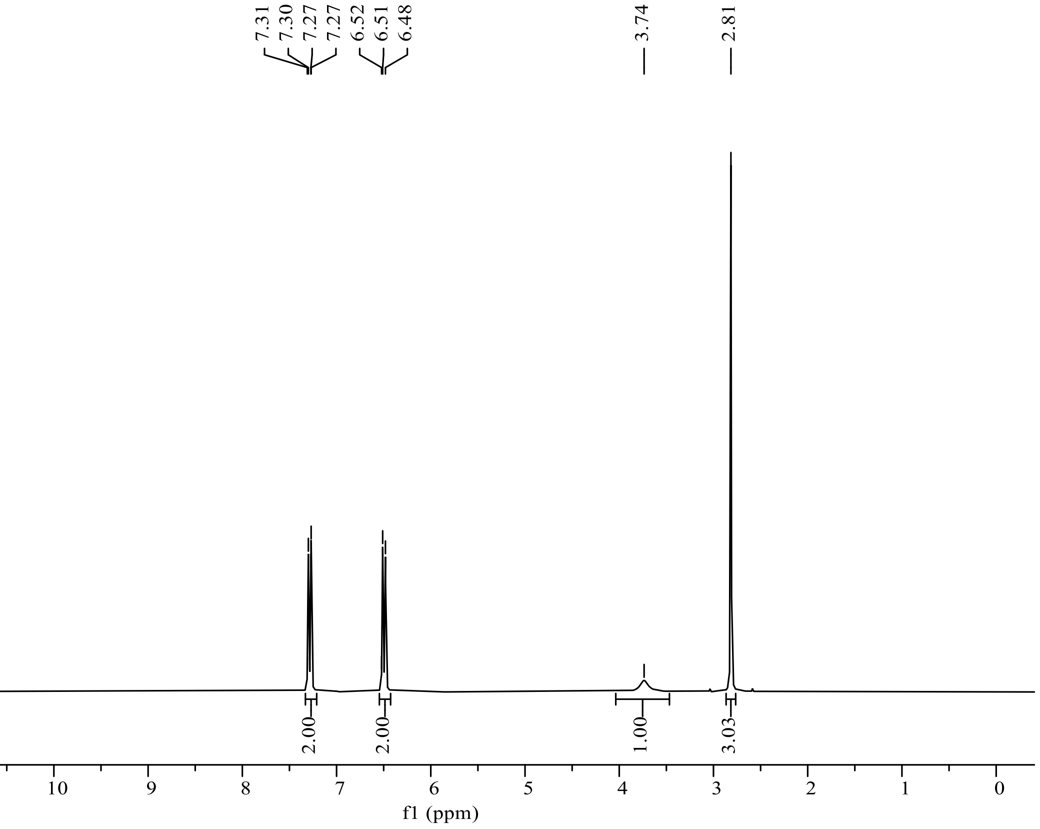

^{13}C NMR (75 MHz, $CDCl_3$):

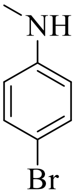

1l

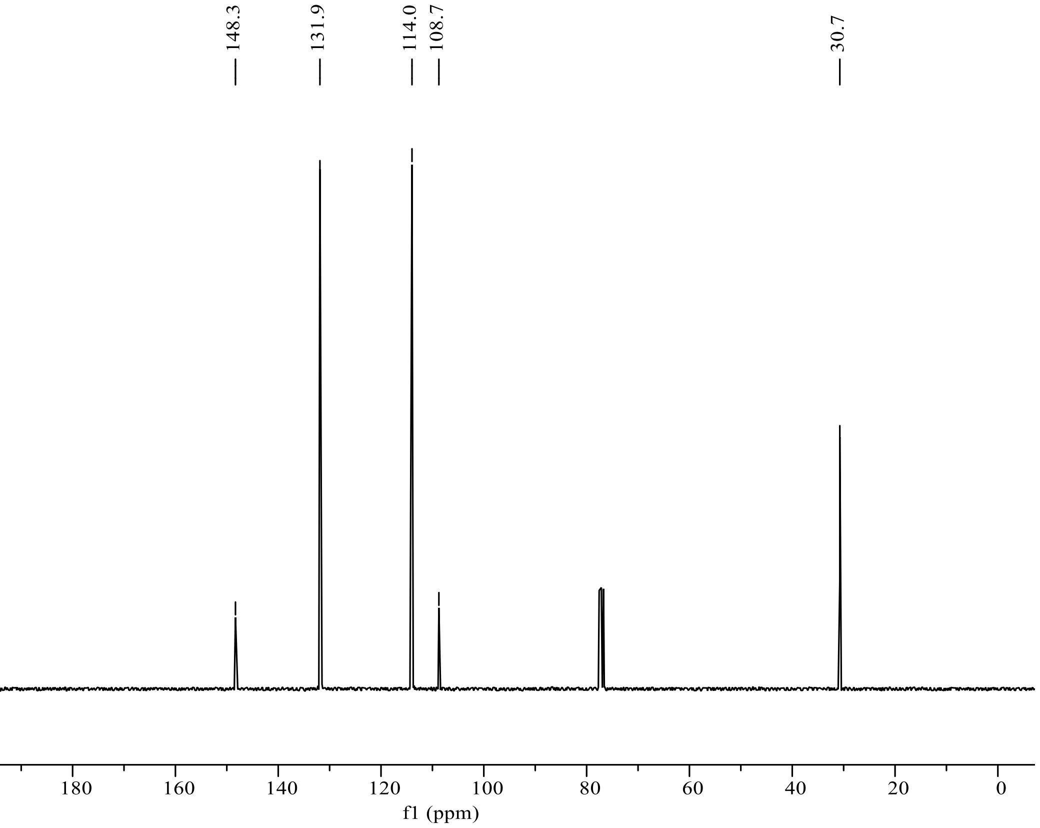

^{1}H NMR (300 MHz, $CDCl_3$):

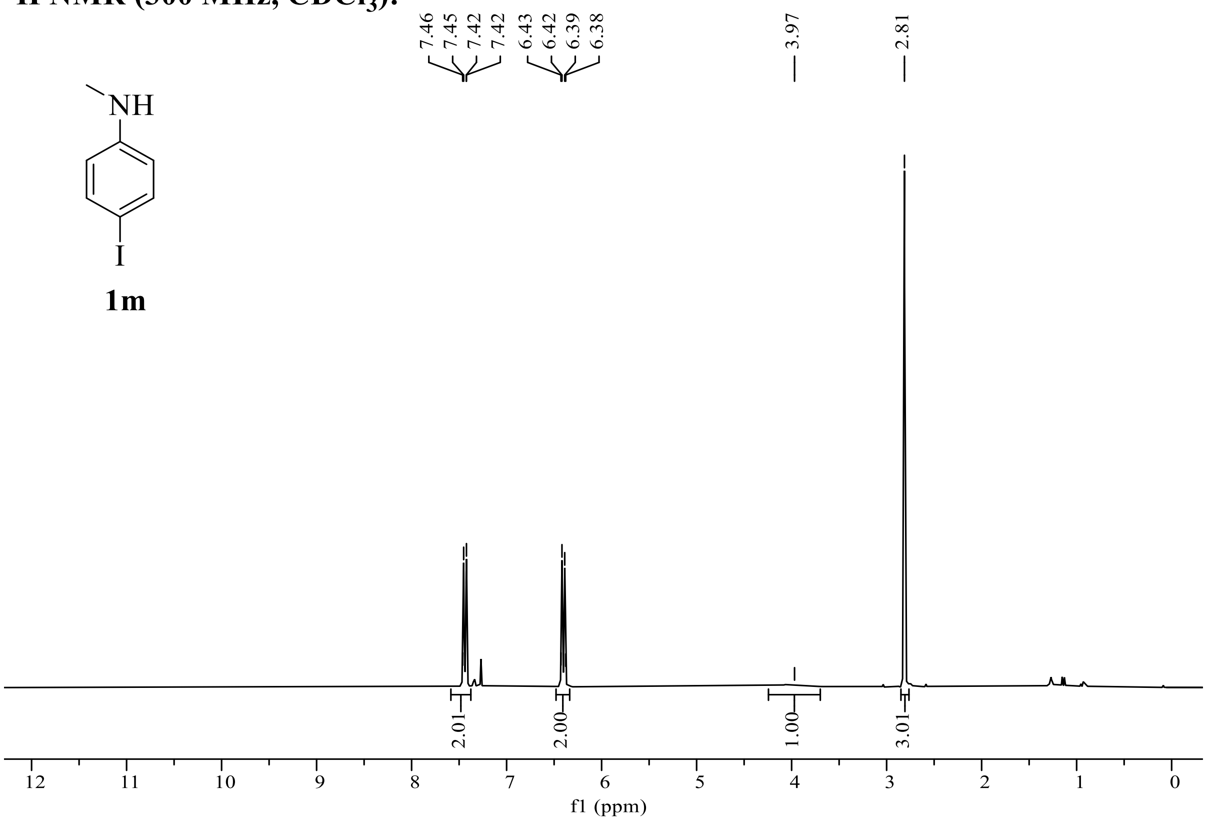

^{13}C NMR (75 MHz, $CDCl_3$):

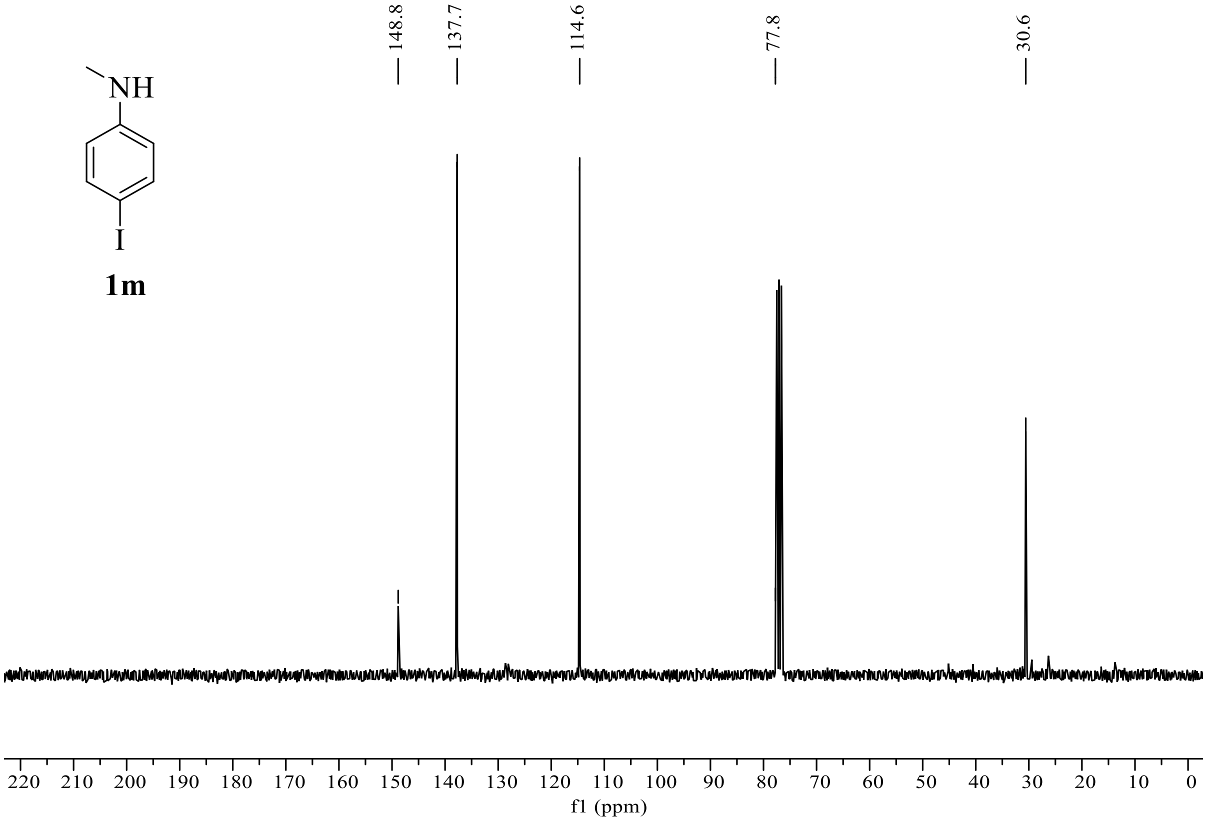

^{1}H NMR (300 MHz, $CDCl_3$):

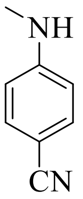

1n

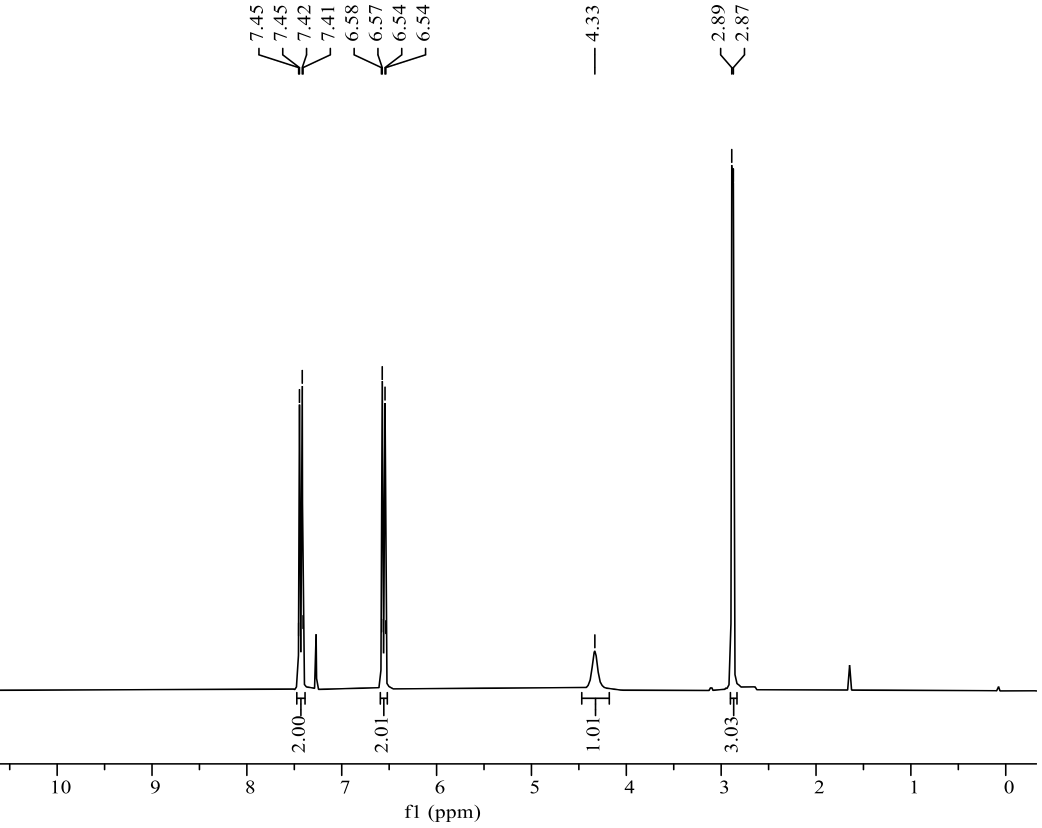

^{13}C NMR (75 MHz, $CDCl_3$):

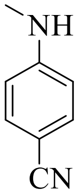

1n

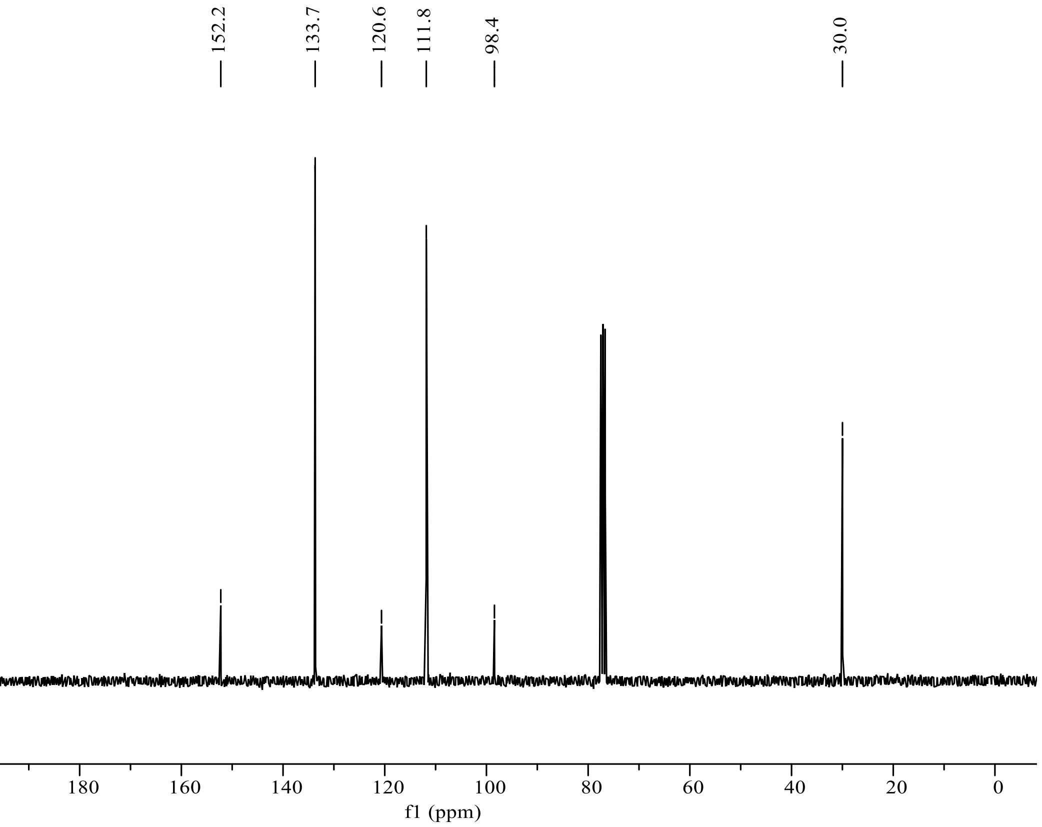

^{1}H NMR (300 MHz, $CDCl_3$):

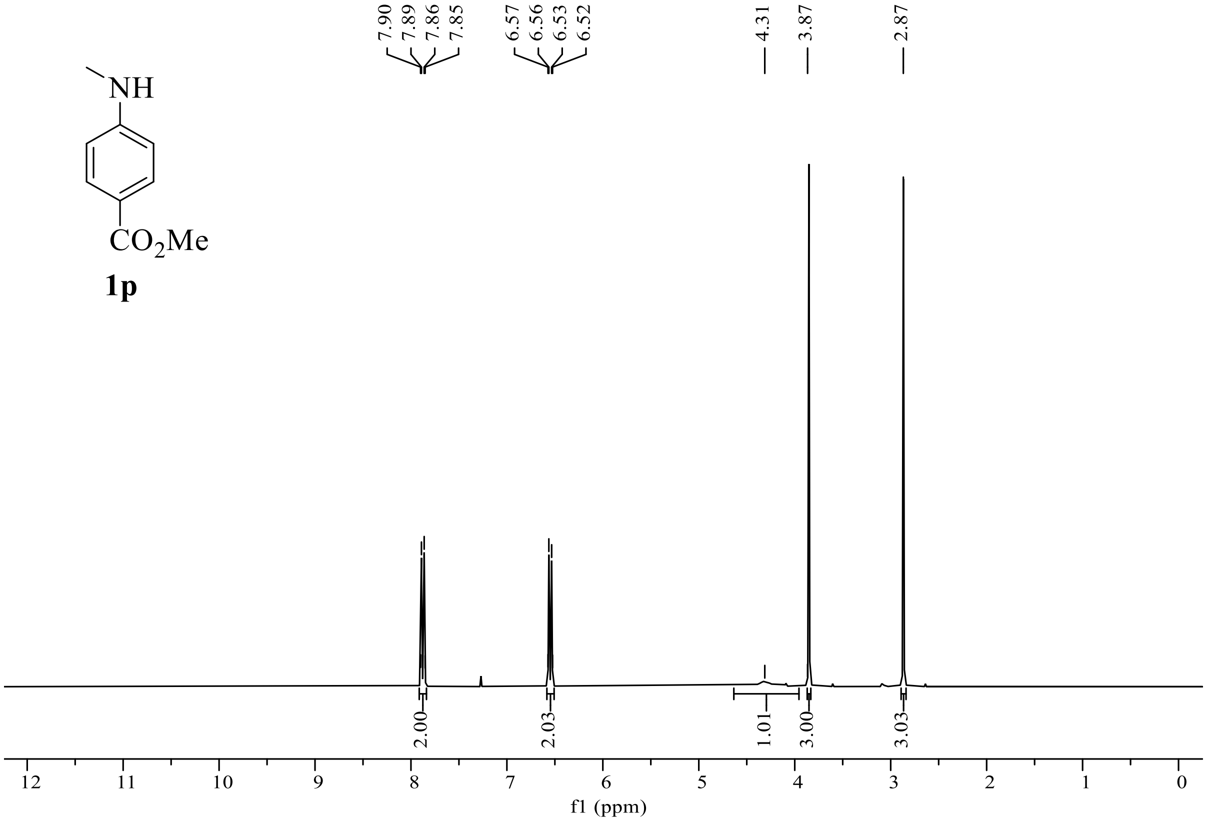

^{13}C NMR (75 MHz, $CDCl_3$):

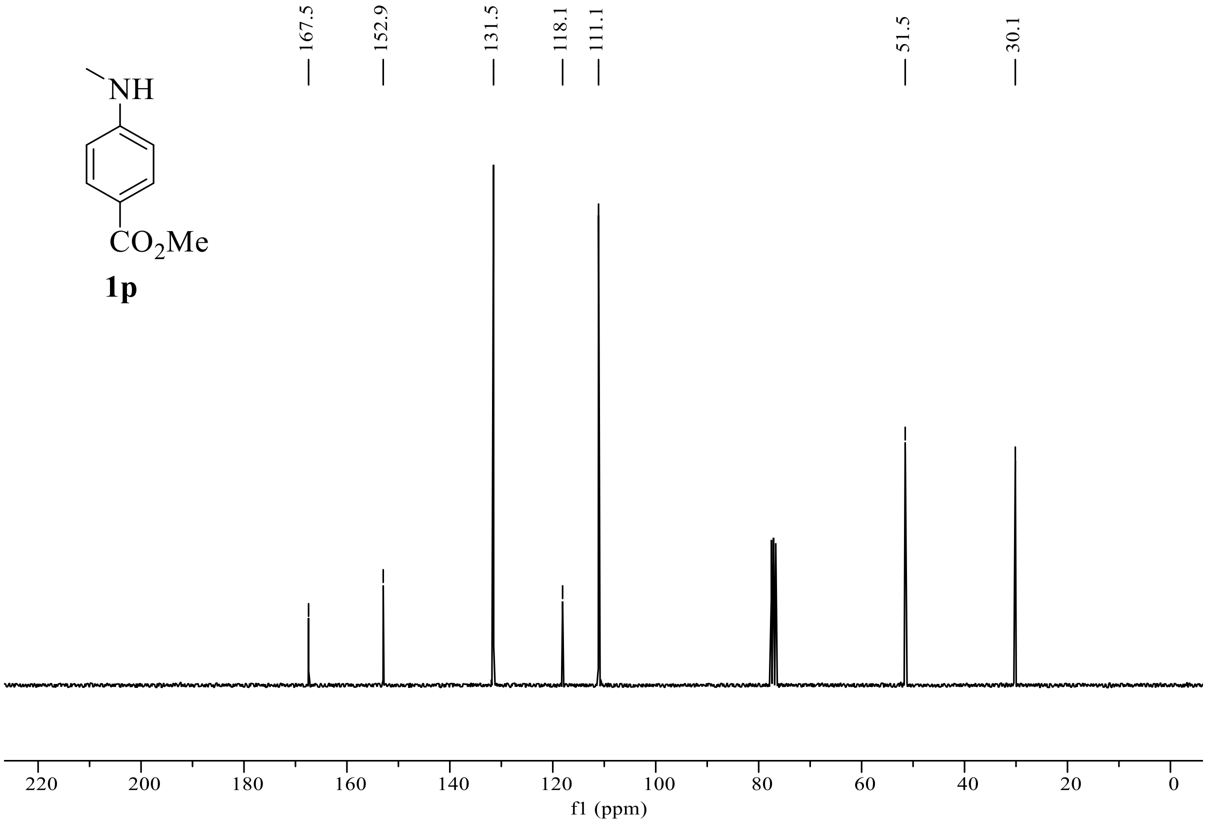

^{1}H NMR (300 MHz, $CDCl_3$):

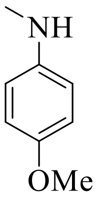

1q

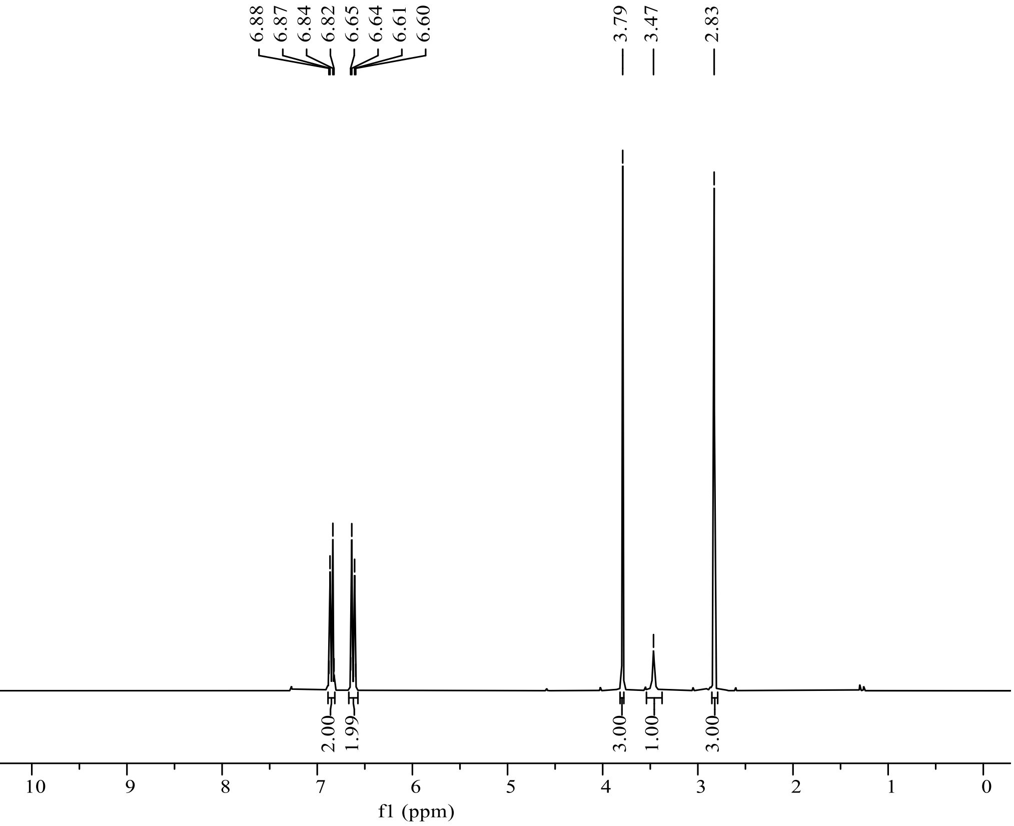

^{13}C NMR (75 MHz, $CDCl_3$):

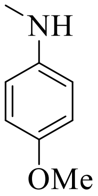

1q

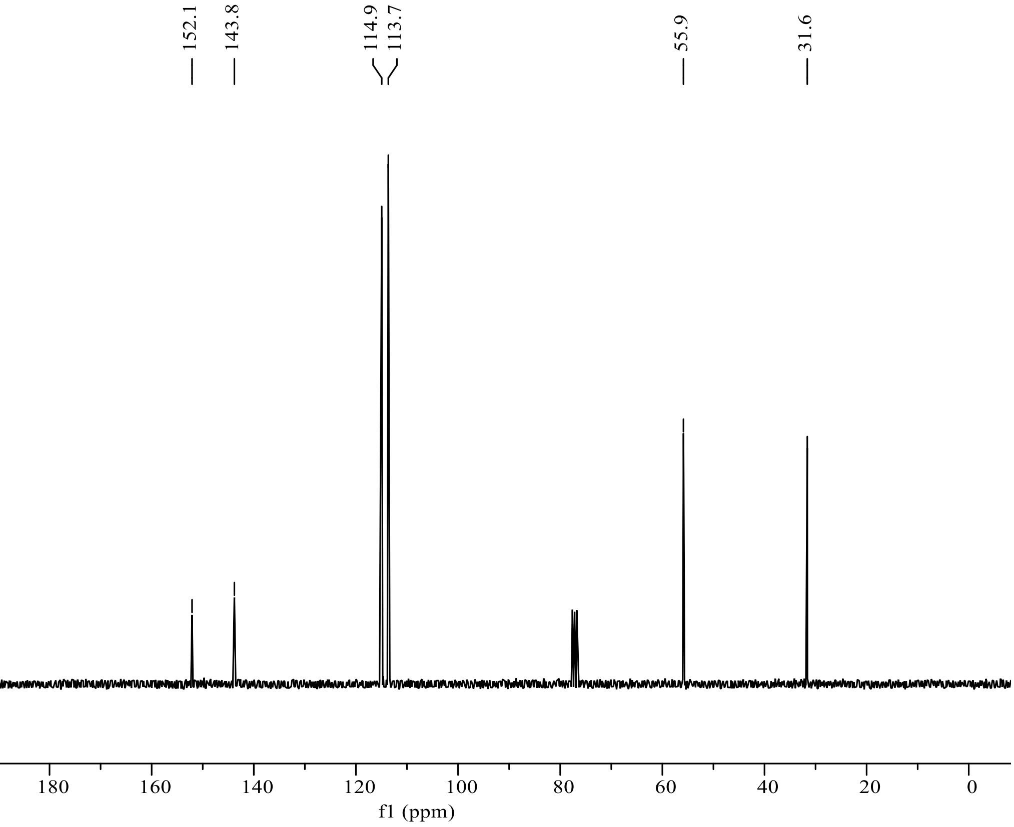

^{1}H NMR (300 MHz, $CDCl_3$):

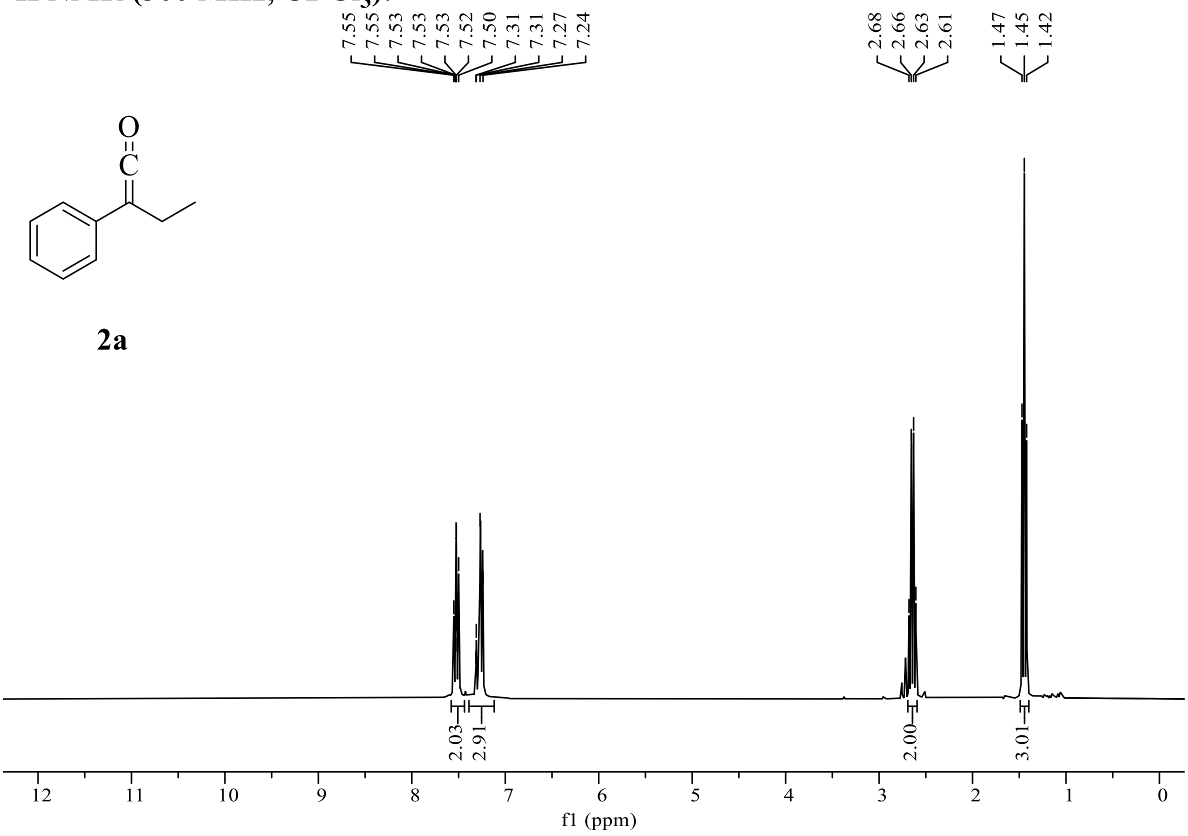

^{13}C NMR (75 MHz, $CDCl_3$):

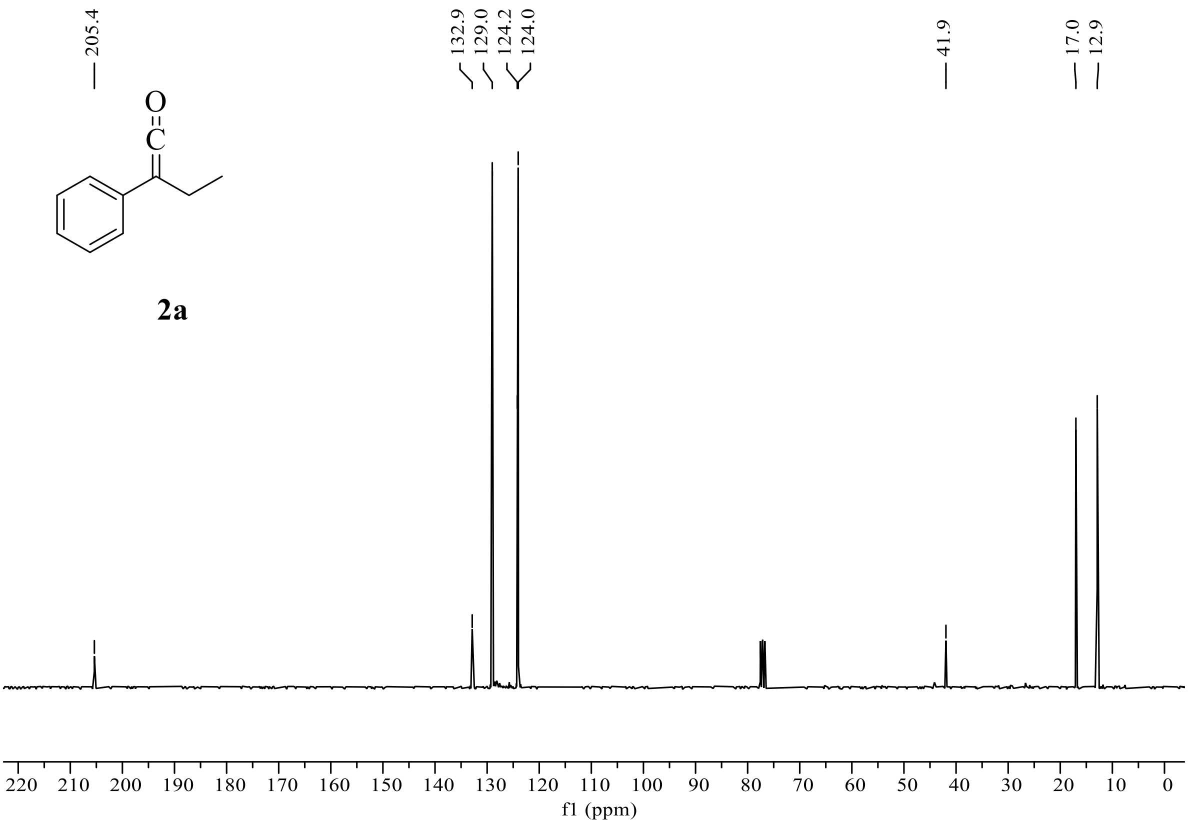

^{1}H NMR (300 MHz, CDCl$_3$):

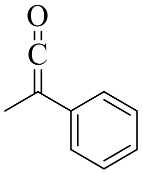

2b

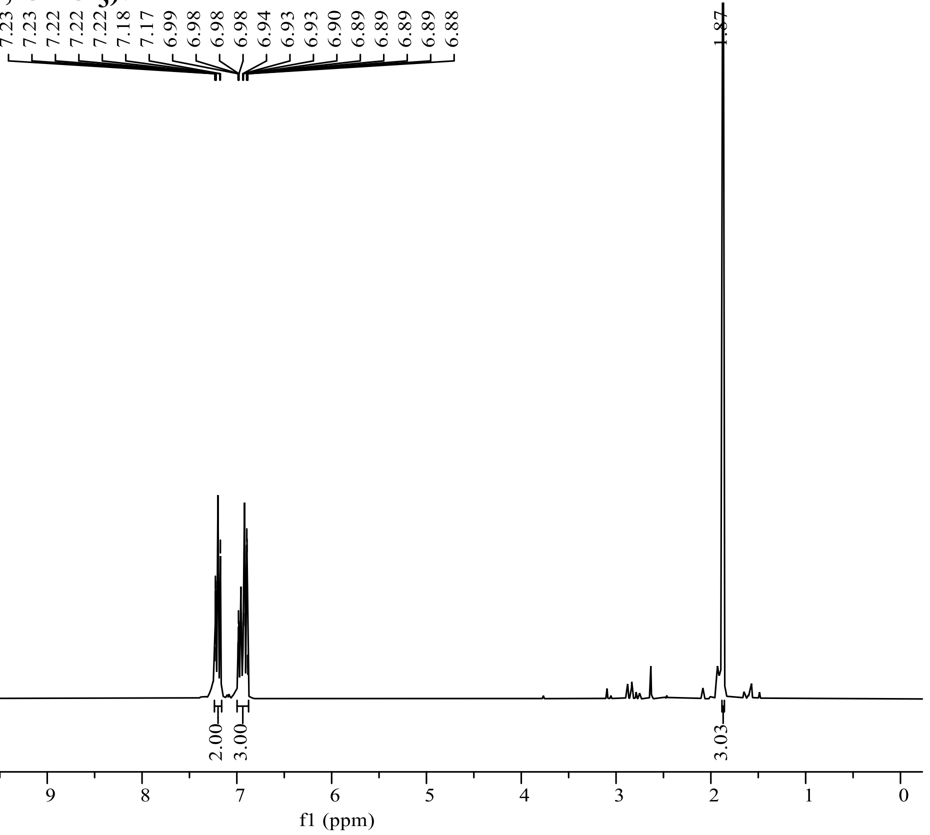

^{13}C NMR (75 MHz, CDCl$_3$):

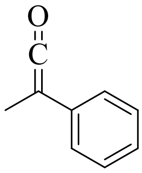

2b

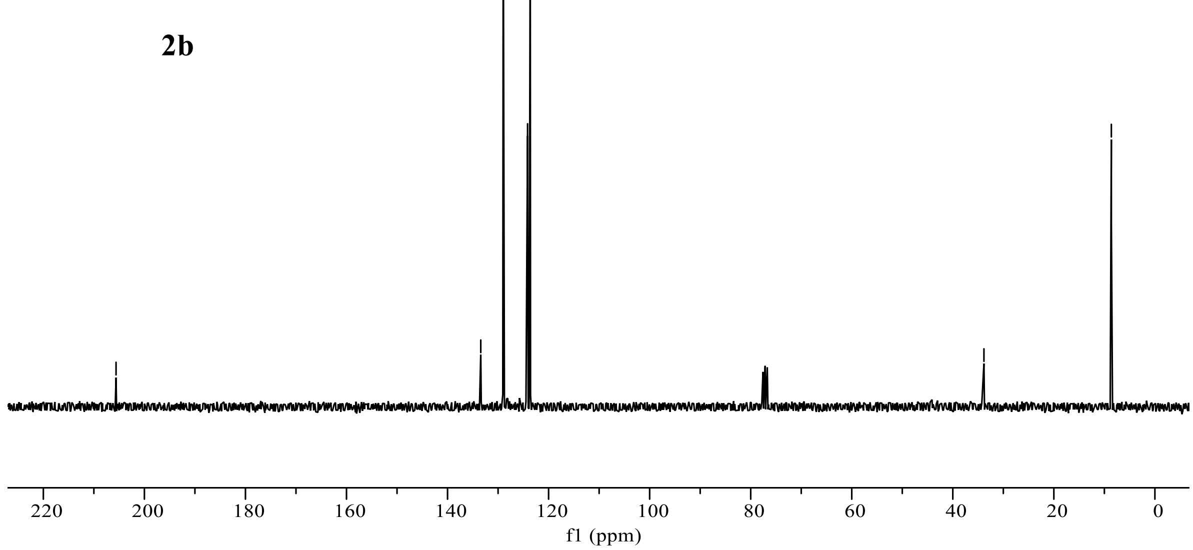

^{1}H NMR (300 MHz, $CDCl_3$):

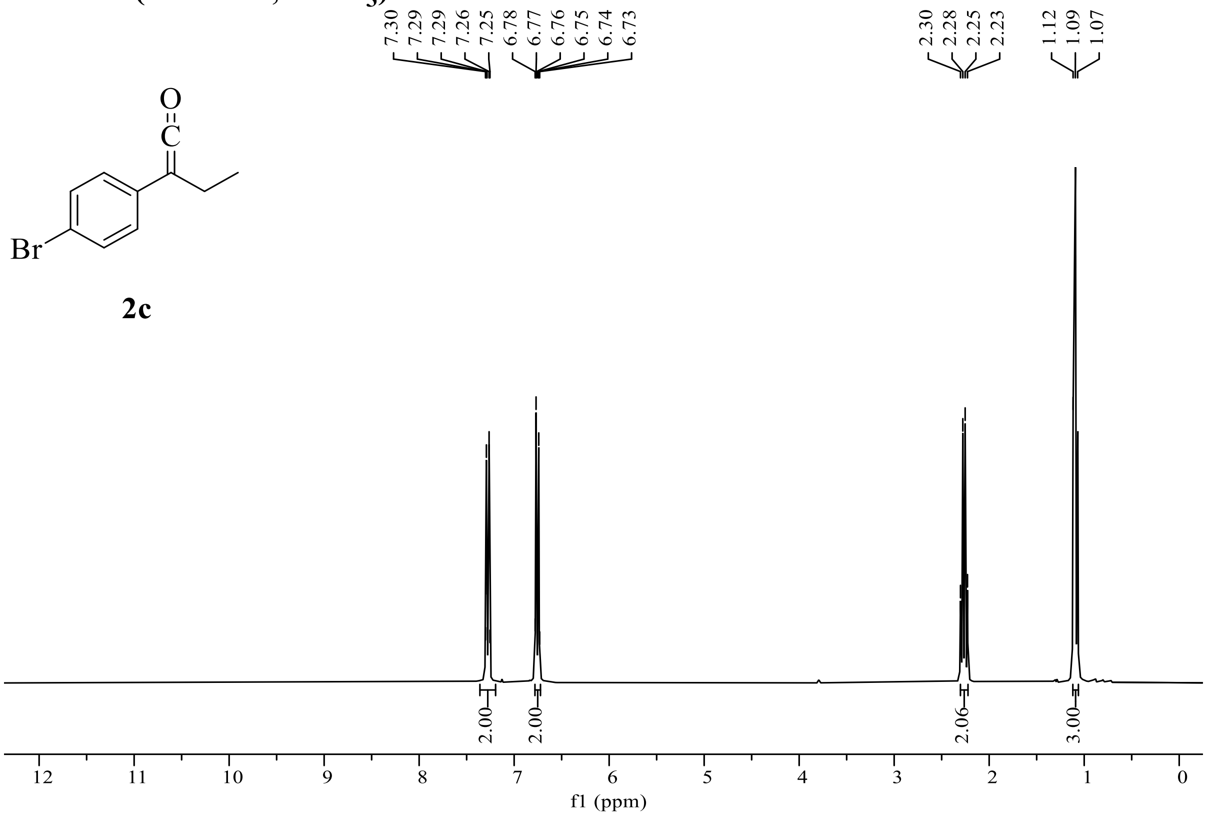

^{13}C NMR (75 MHz, $CDCl_3$):

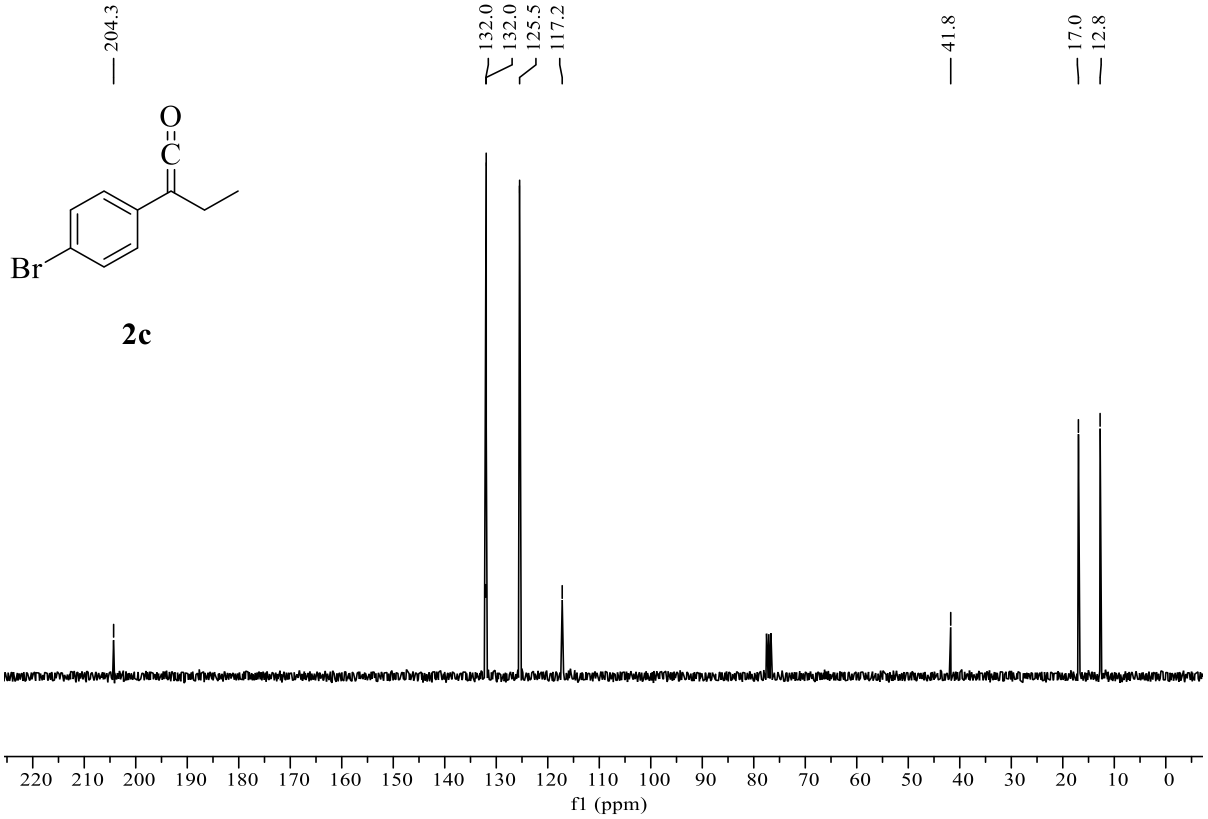

^{1}H NMR (300 MHz, $CDCl_3$):

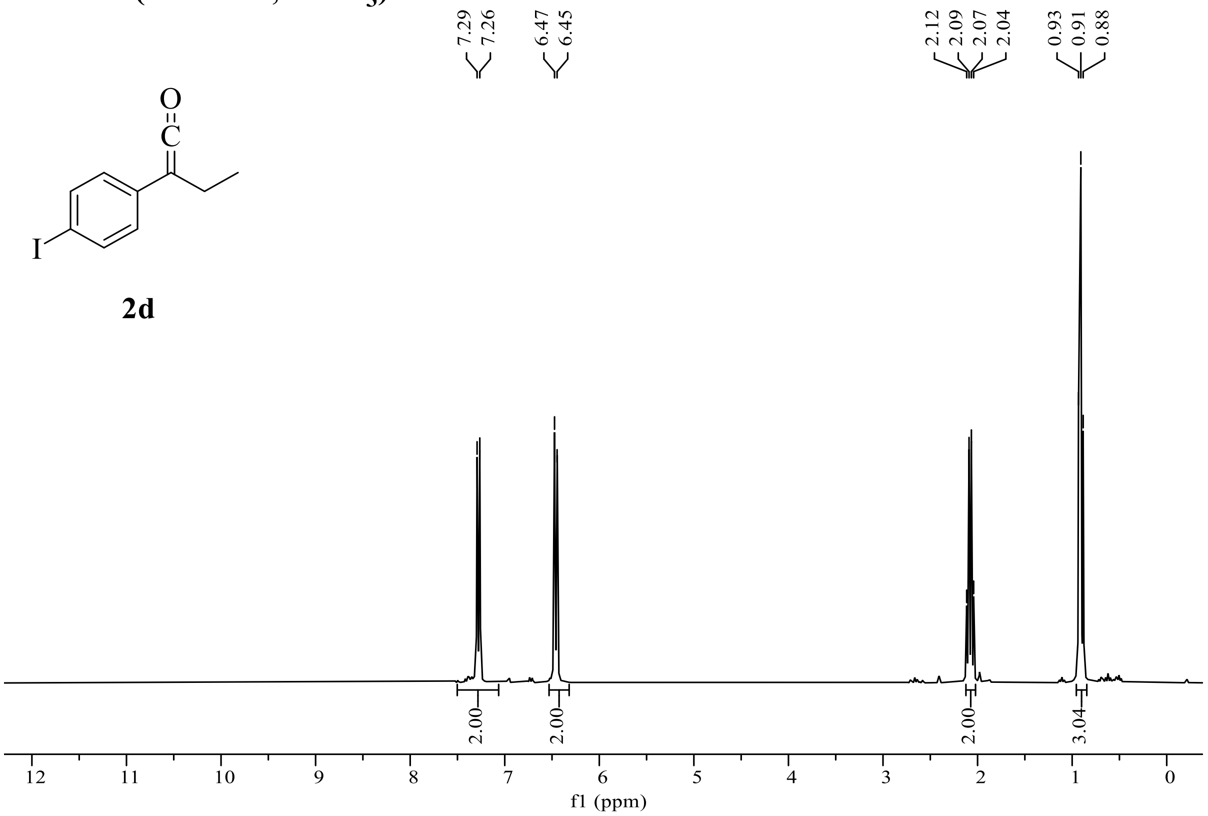

^{13}C NMR (75 MHz, $CDCl_3$):

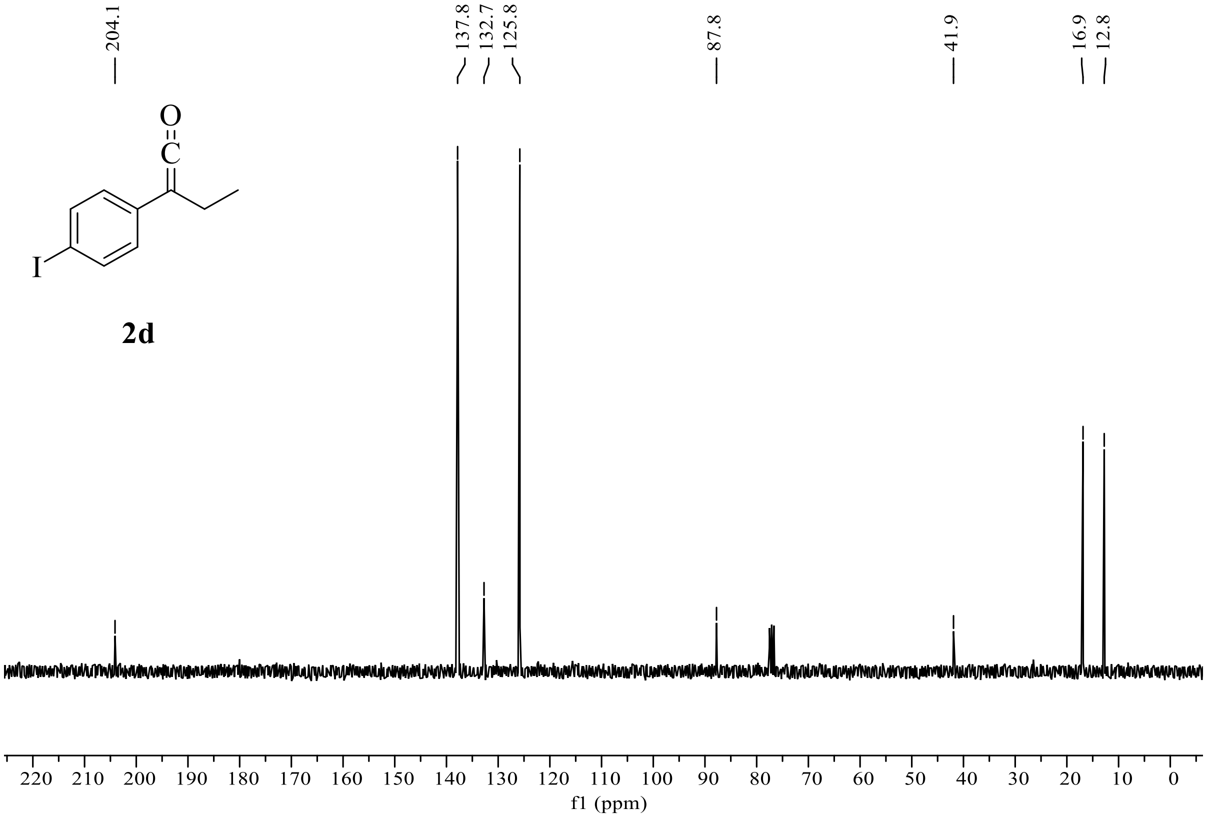

^{1}H NMR (300 MHz, $CDCl_3$):

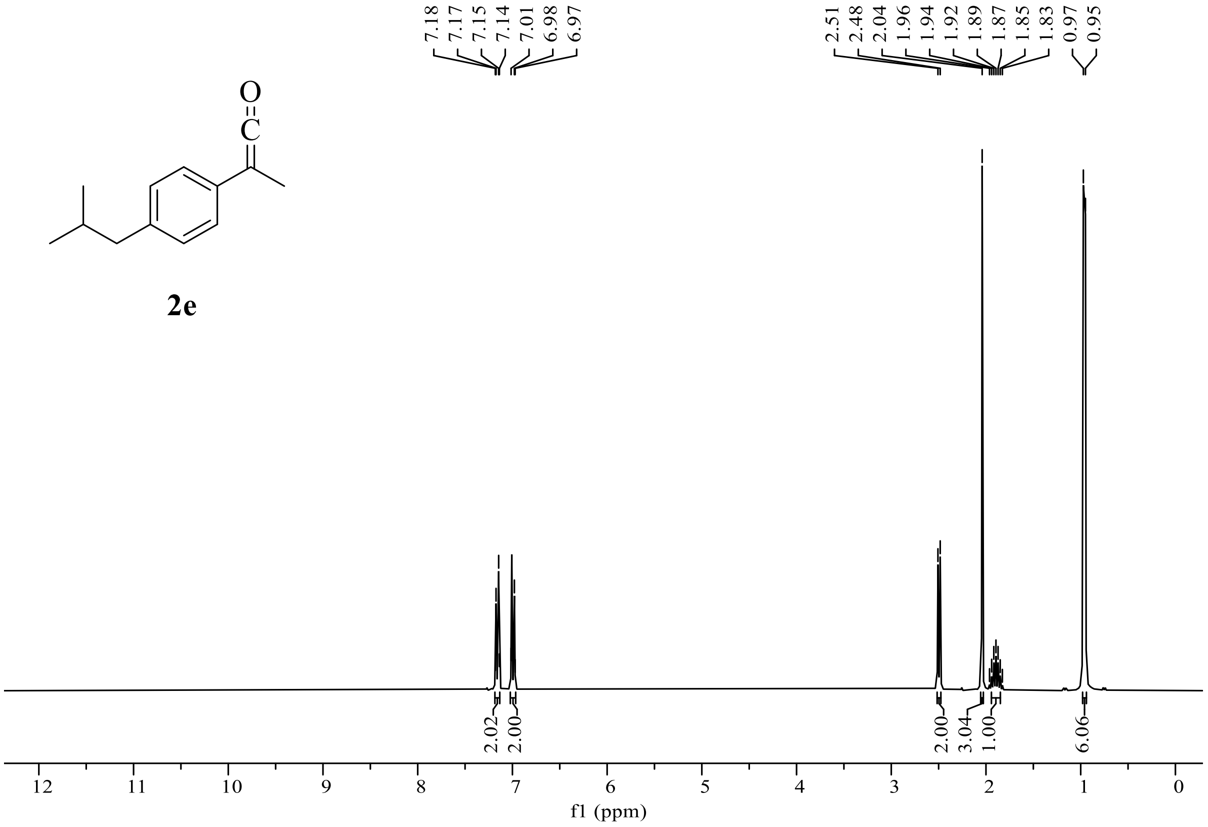

^{13}C NMR (75 MHz, $CDCl_3$):

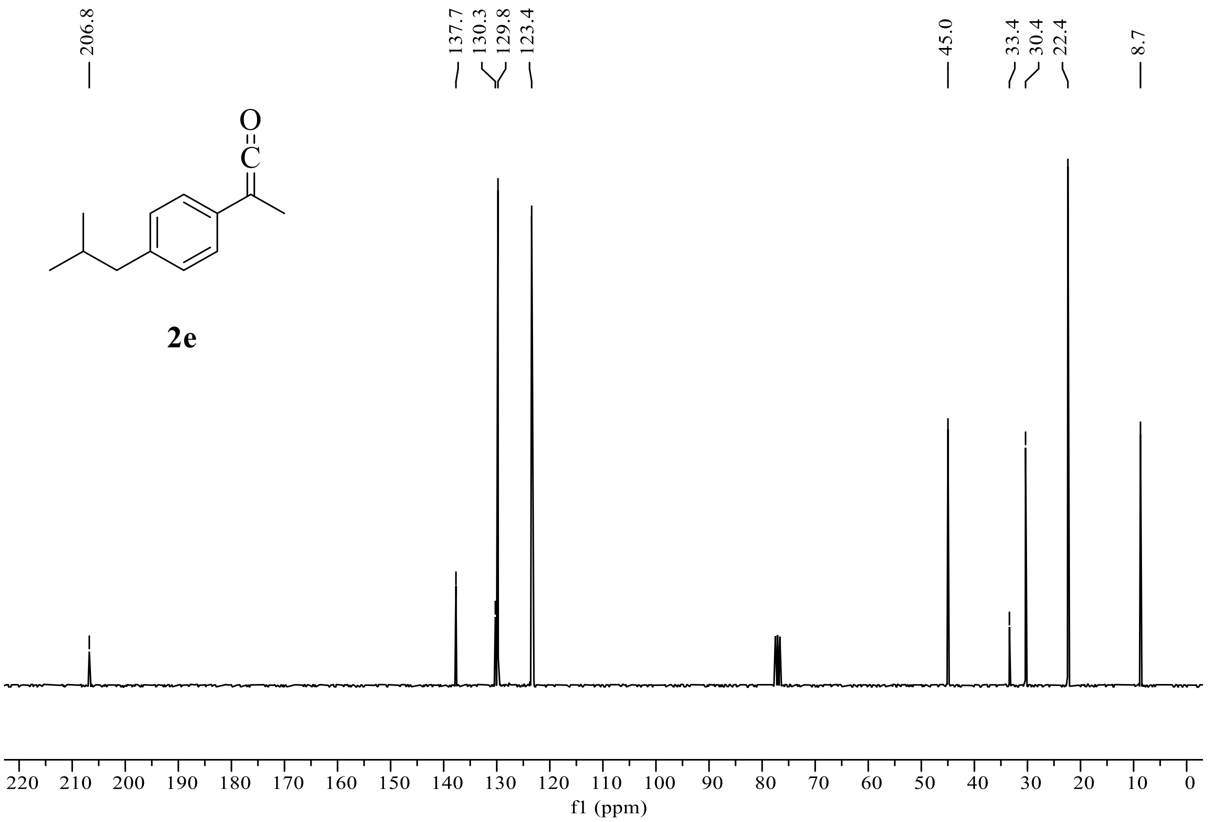

^{1}H NMR (300 MHz, $CDCl_3$):

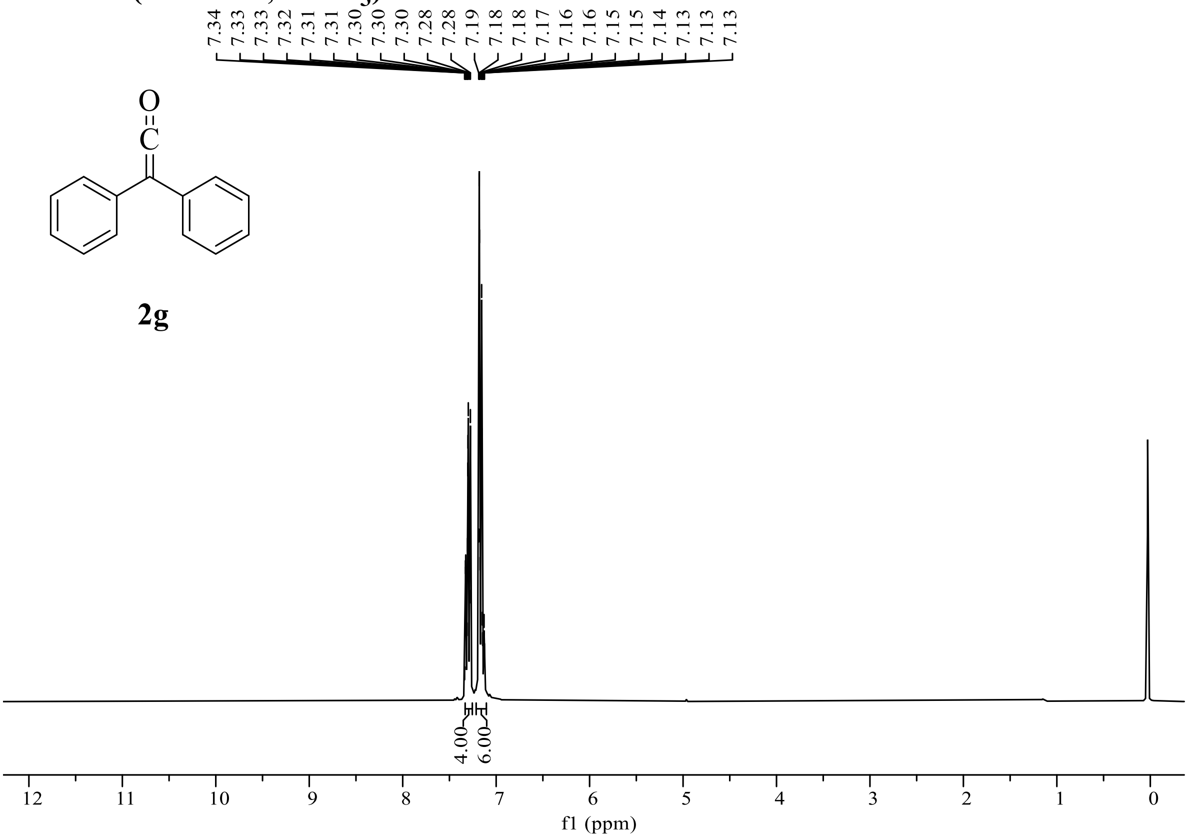

^{13}C NMR (75 MHz, $CDCl_3$):

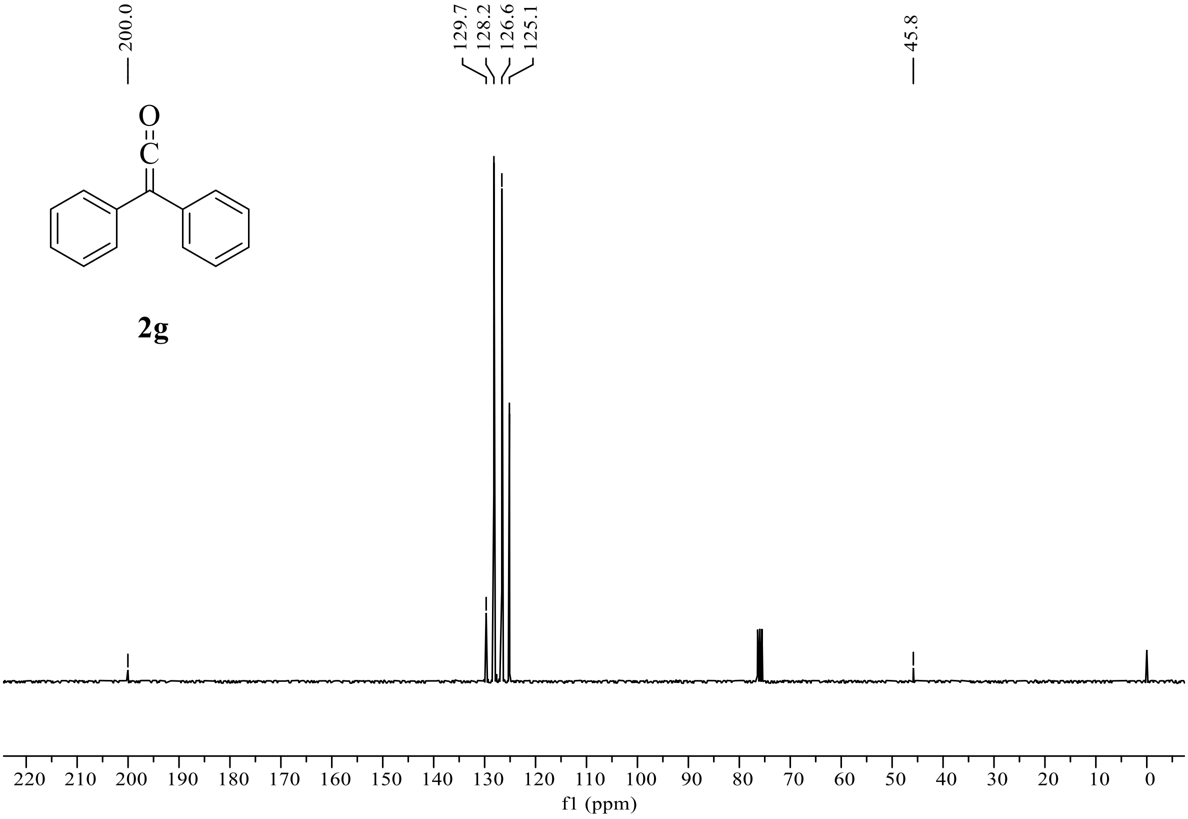

^{1}H NMR (300 MHz, $CDCl_3$):

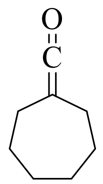

2h

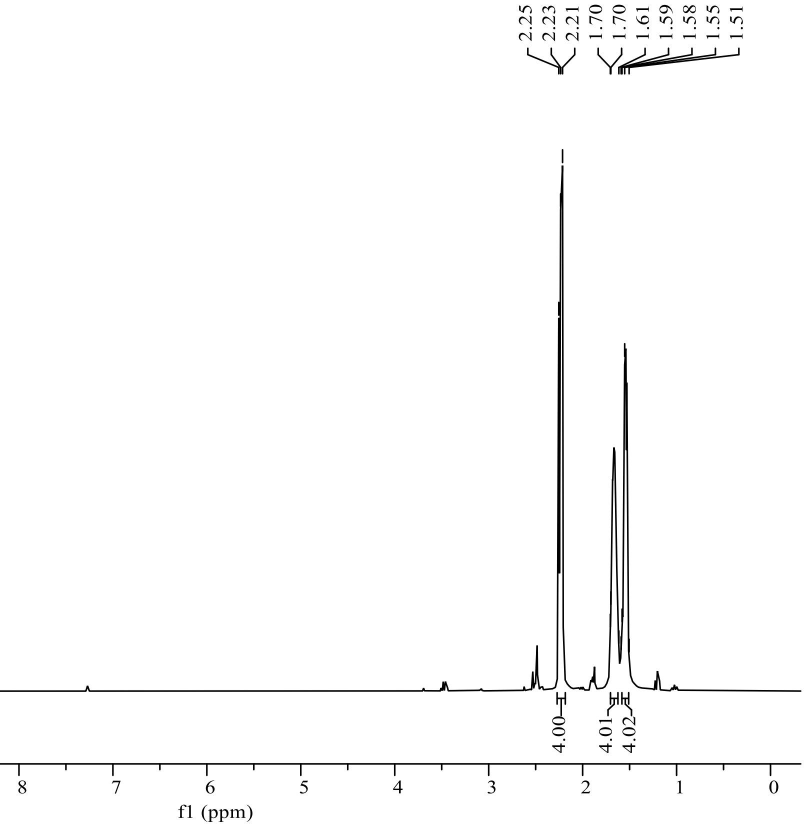

^{1}H NMR (300 MHz, CDCl$_3$):

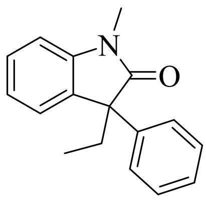

3aa

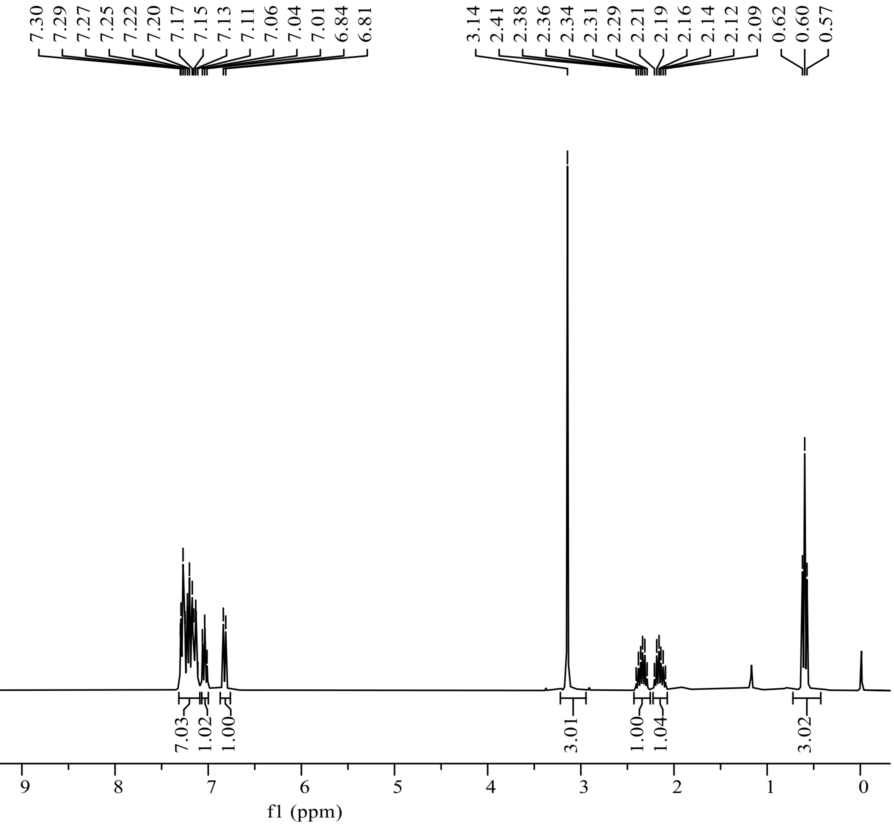

^{13}C NMR (75 MHz, CDCl$_3$):

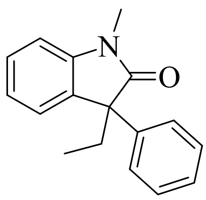

3aa

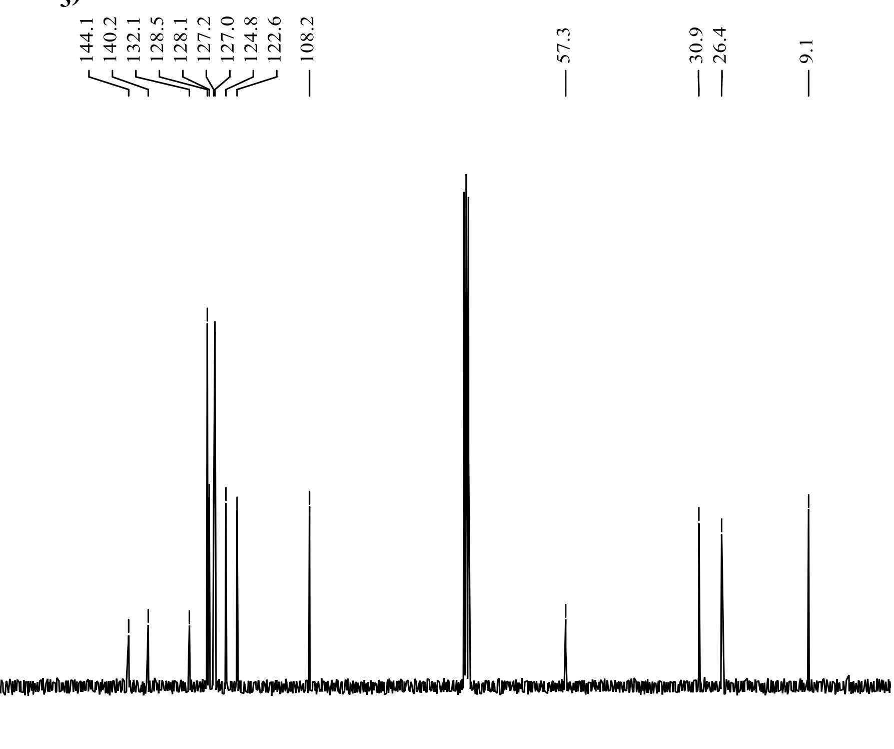

^{1}H NMR (300 MHz, CDCl$_3$):

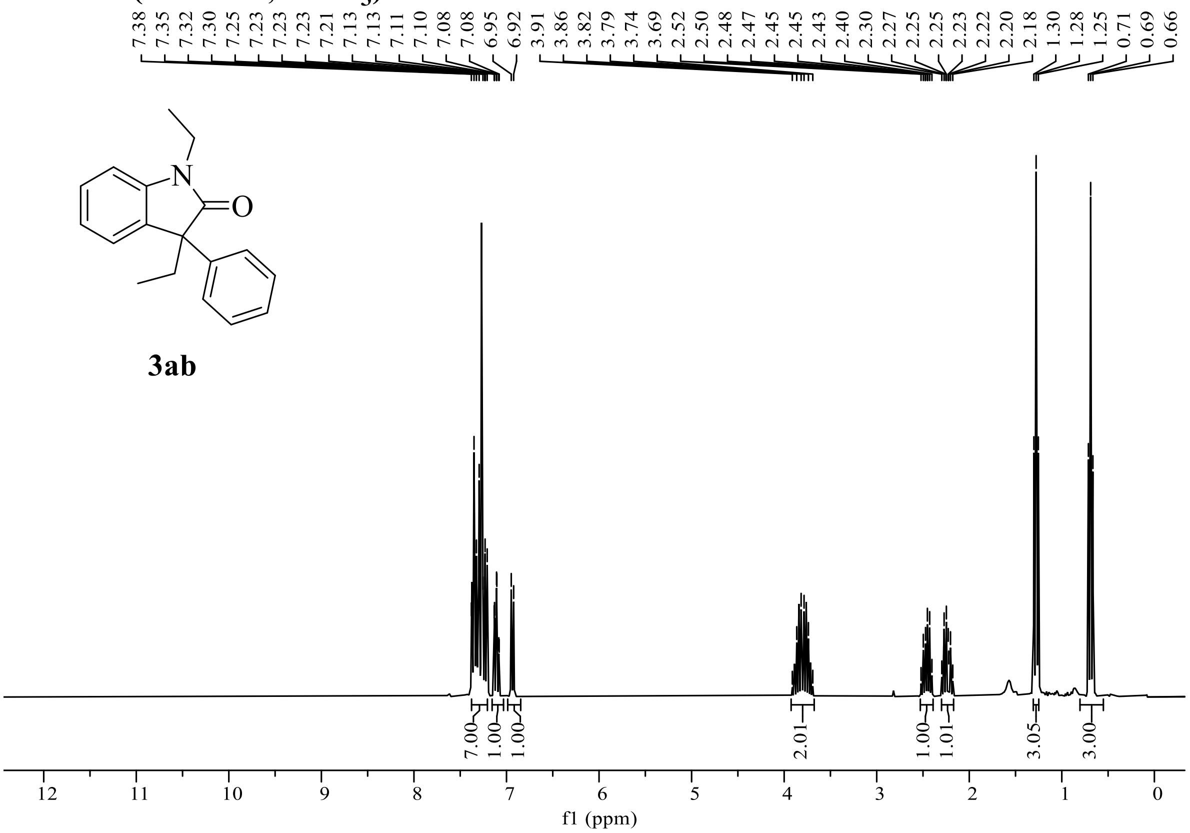

^{13}C NMR (75 MHz, CDCl$_3$):

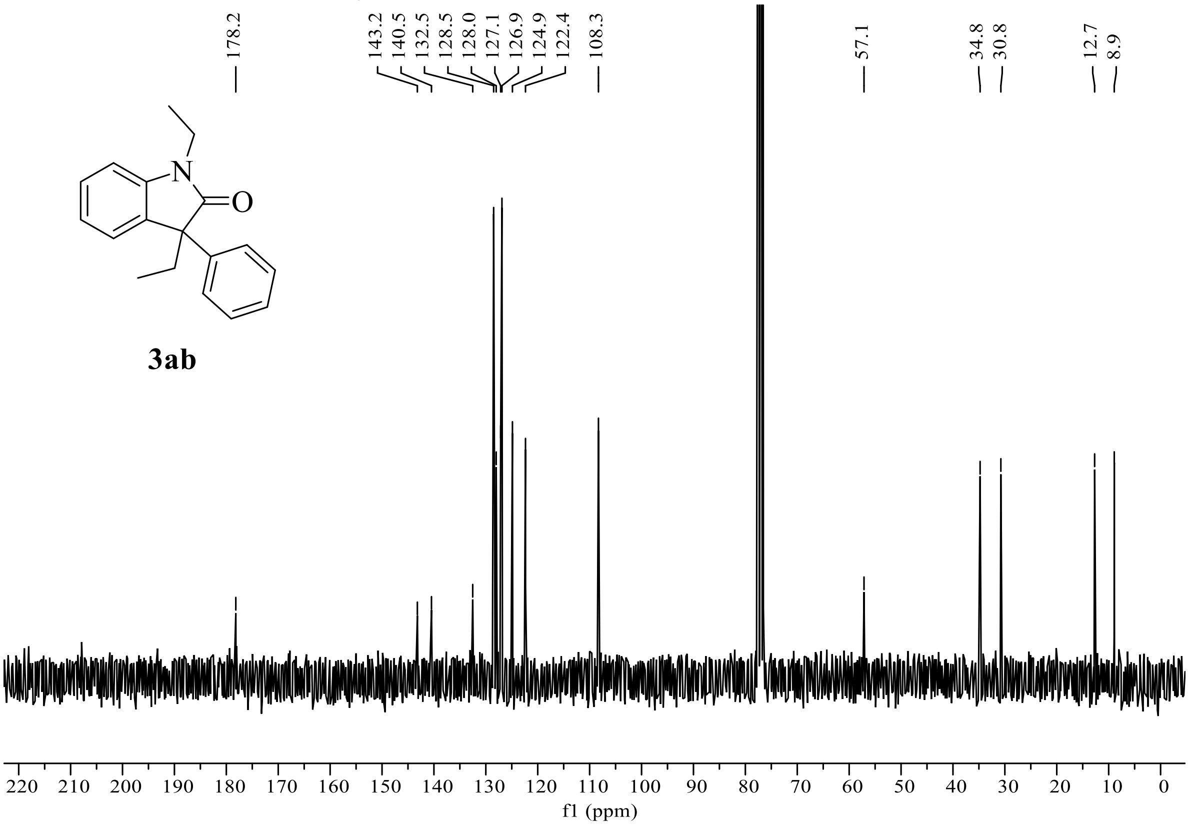

^{1}H NMR (300 MHz, CDCl$_3$):

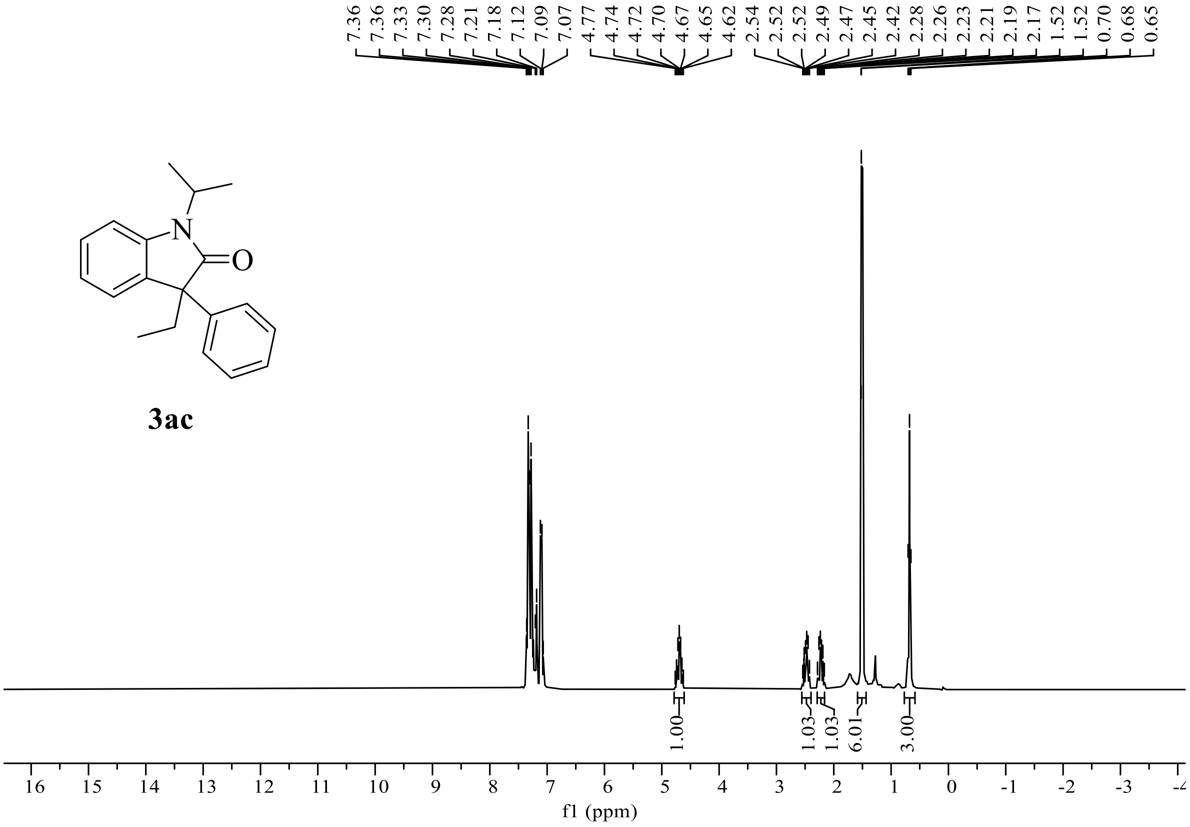

3ac

^{13}C NMR (75 MHz, CDCl$_3$):

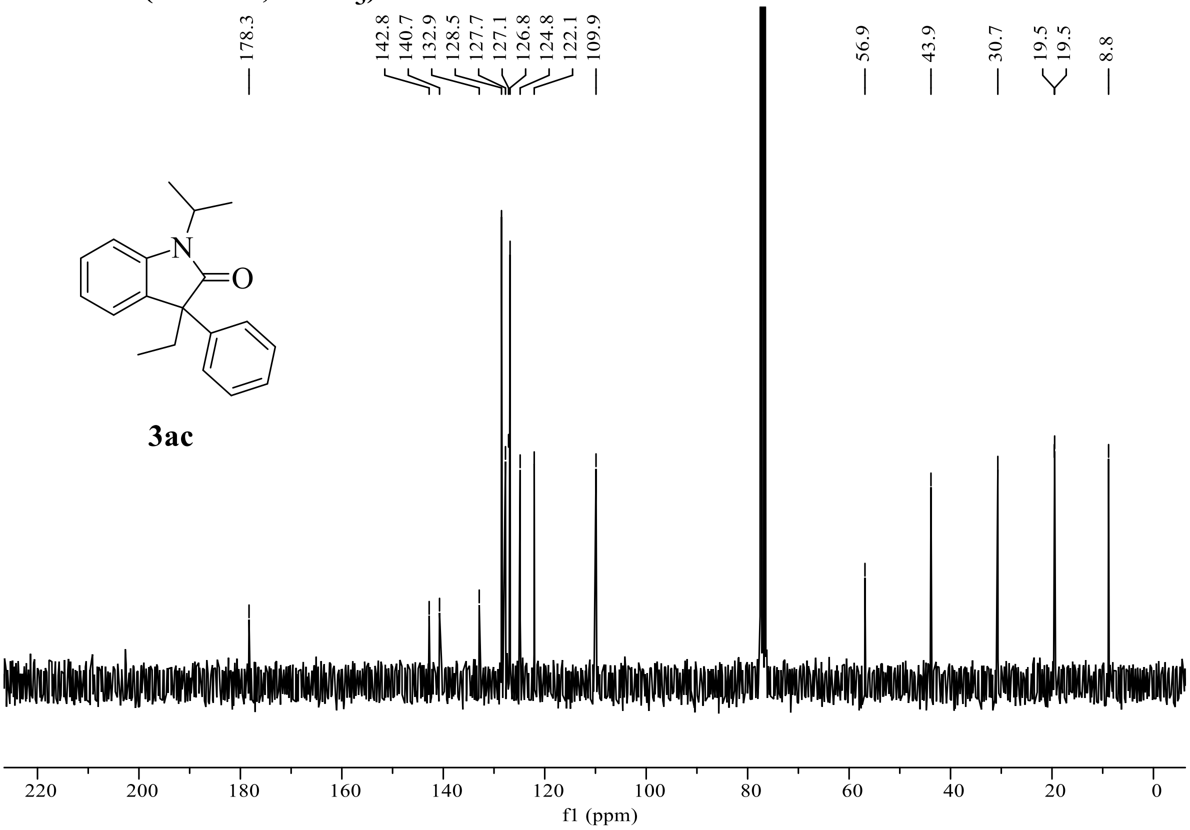

3ac

^{1}H NMR (600 MHz, $CDCl_3$):

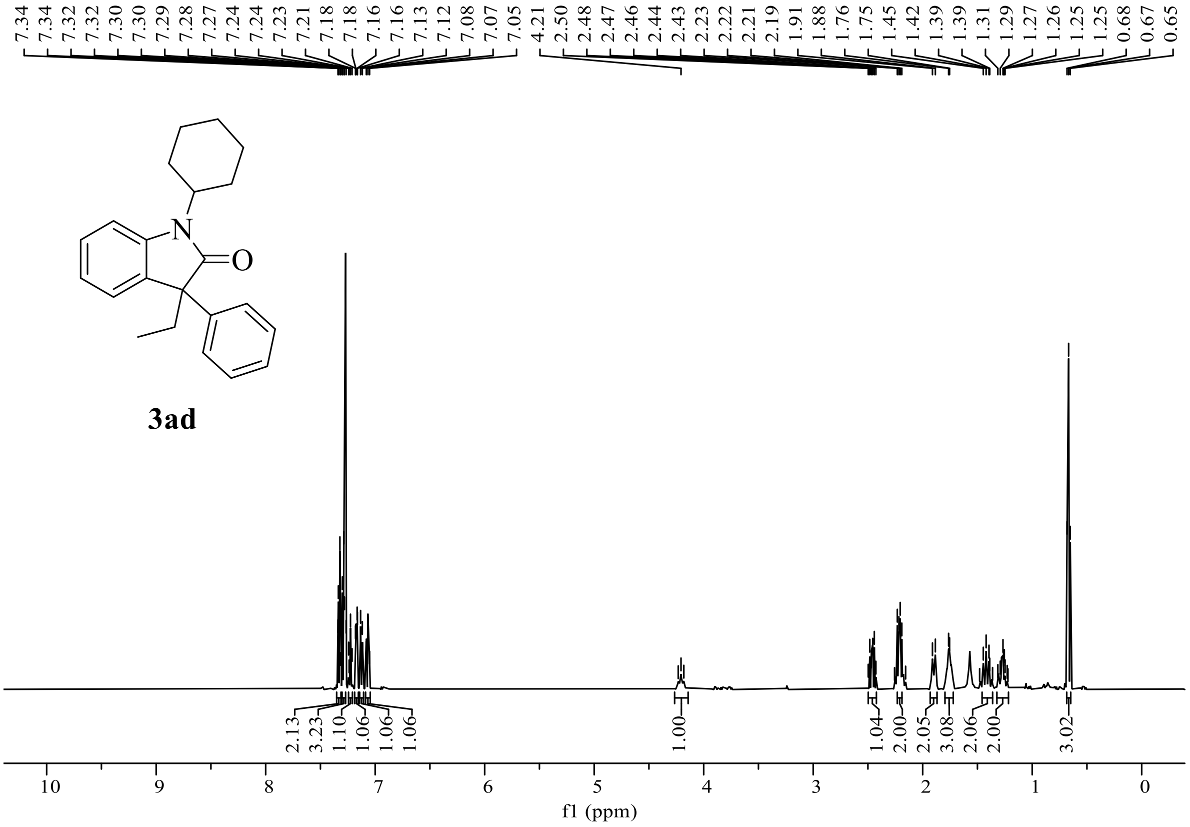

^{13}C NMR (151 MHz, $CDCl_3$):

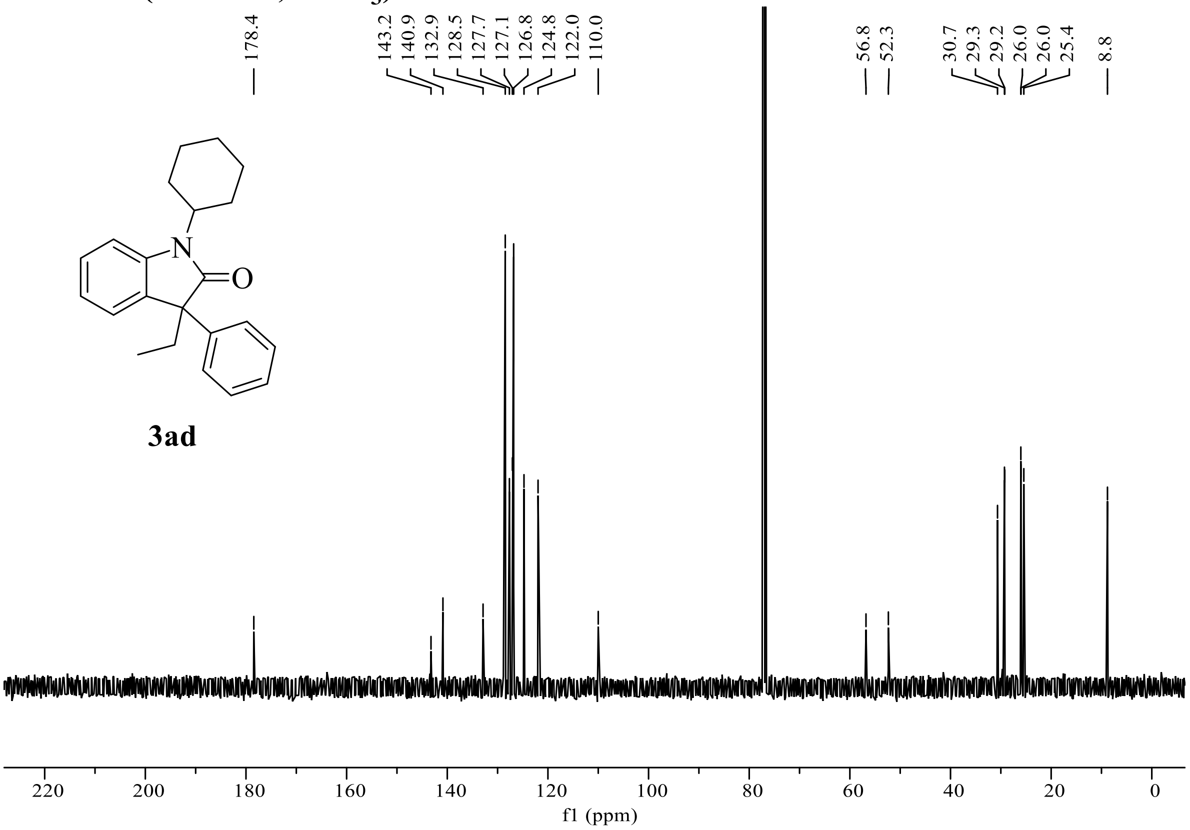

^{1}H NMR (300 MHz, $CDCl_3$):

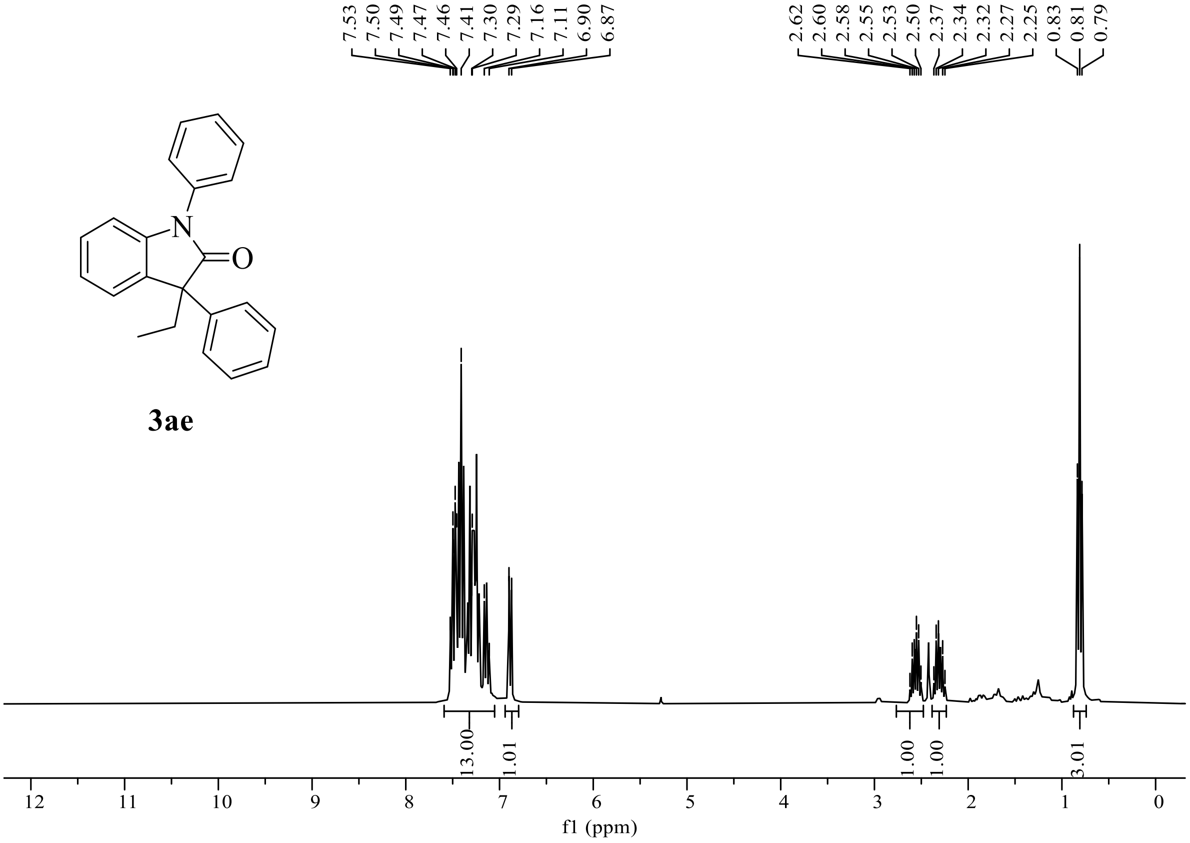

^{13}C NMR (75 MHz, $CDCl_3$):

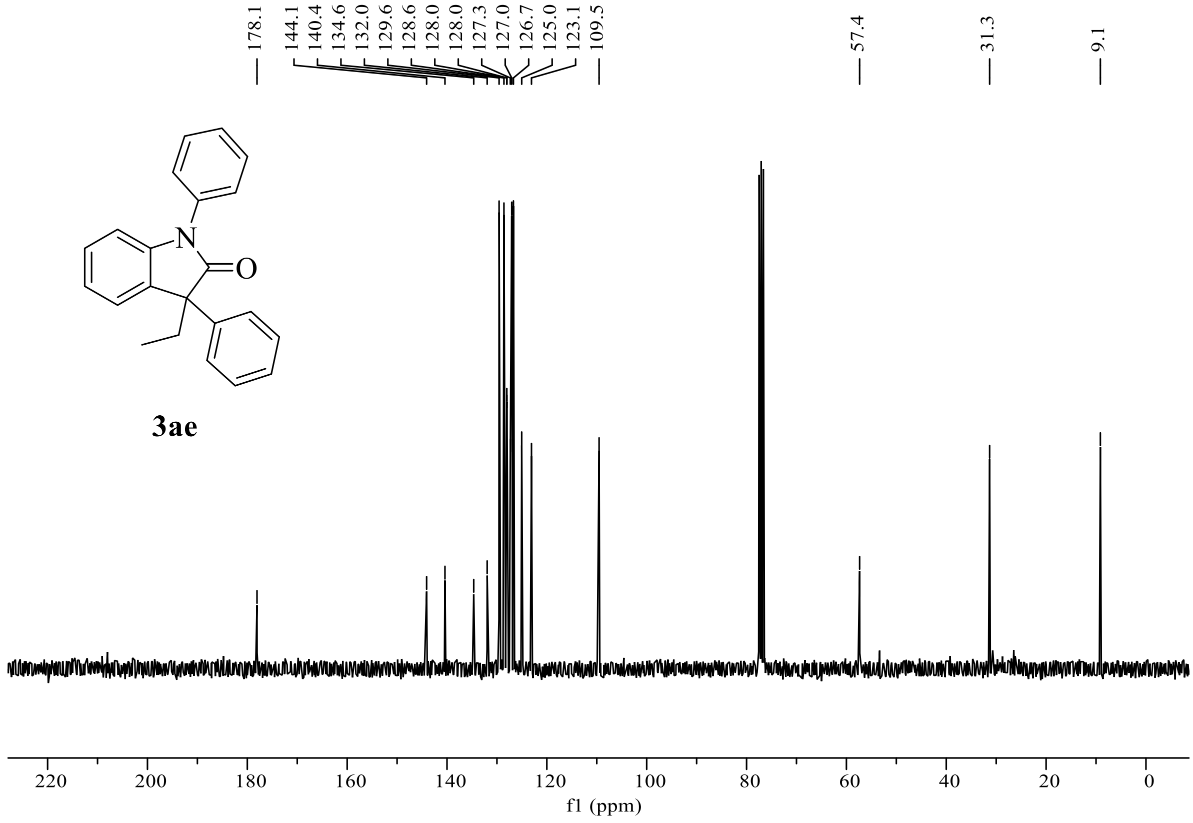

^{1}H NMR (300 MHz, $CDCl_3$):

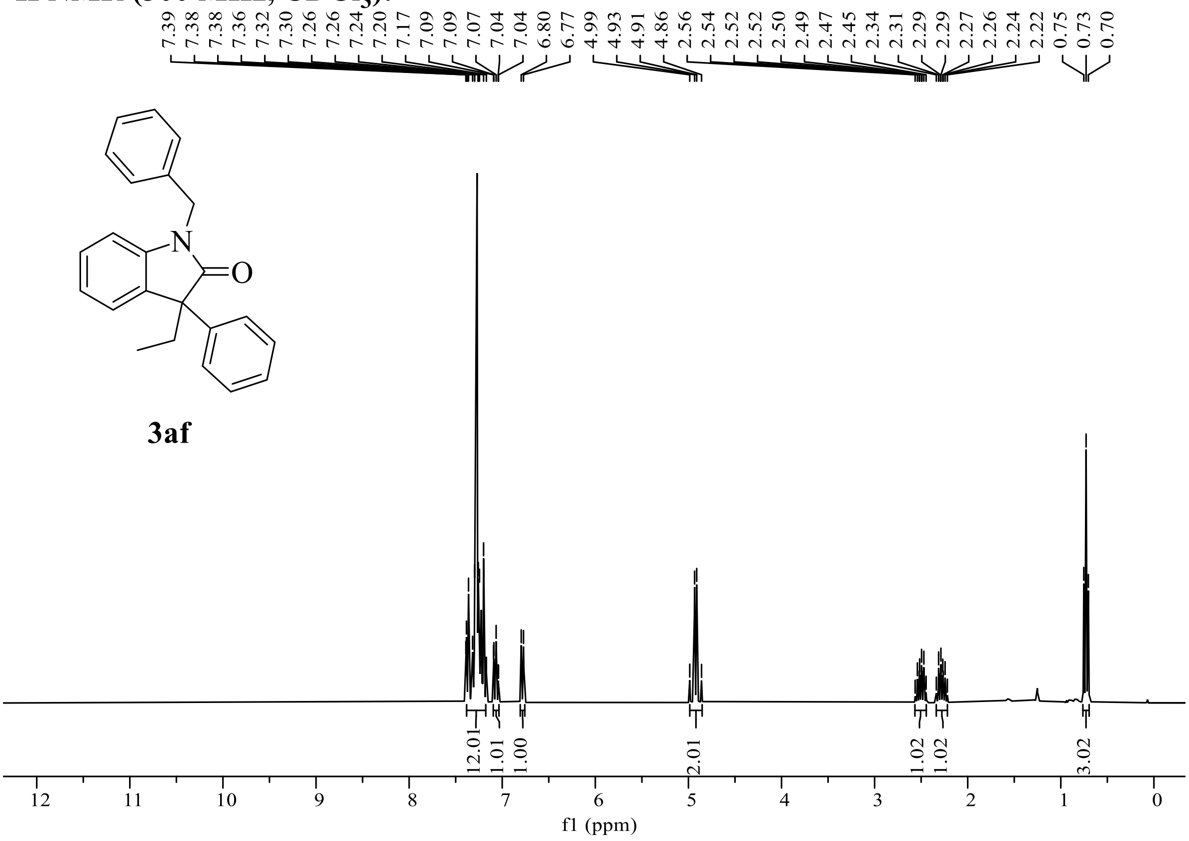

^{13}C NMR (75 MHz, $CDCl_3$):

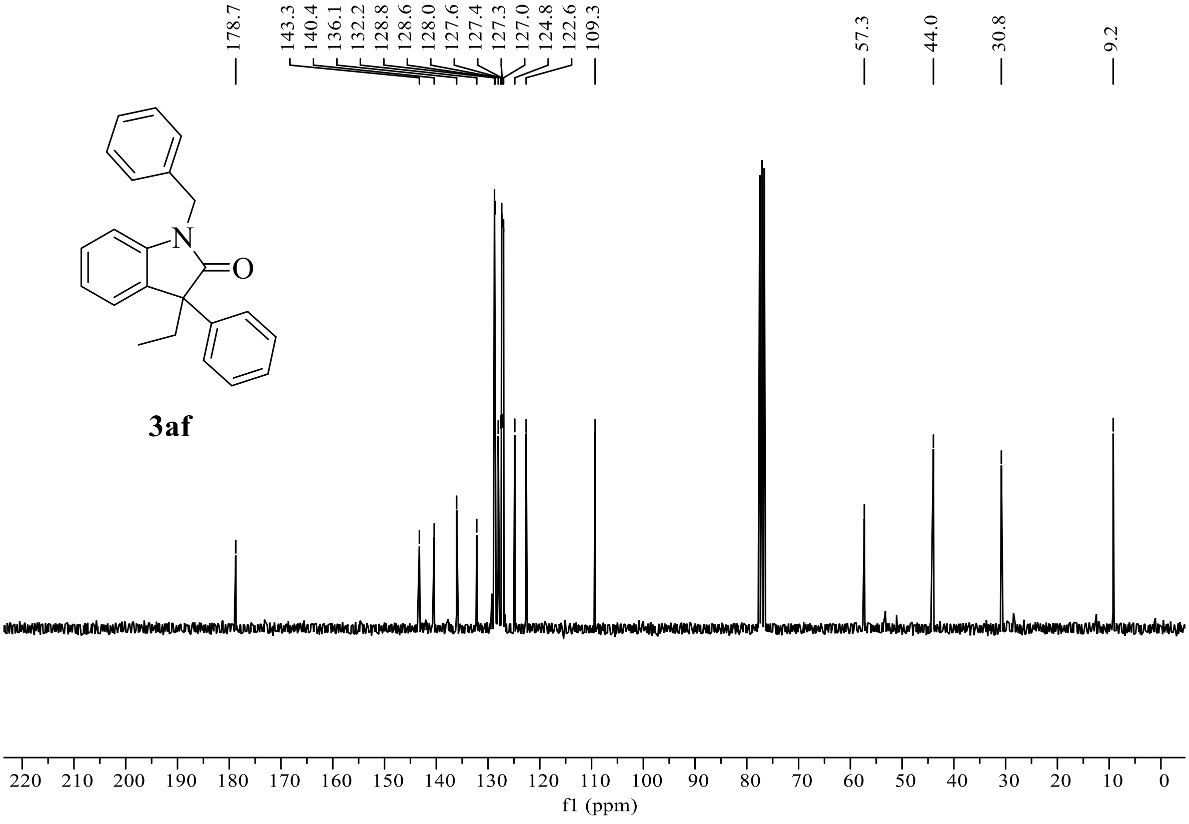

^{1}H NMR (600 MHz, $CDCl_3$):

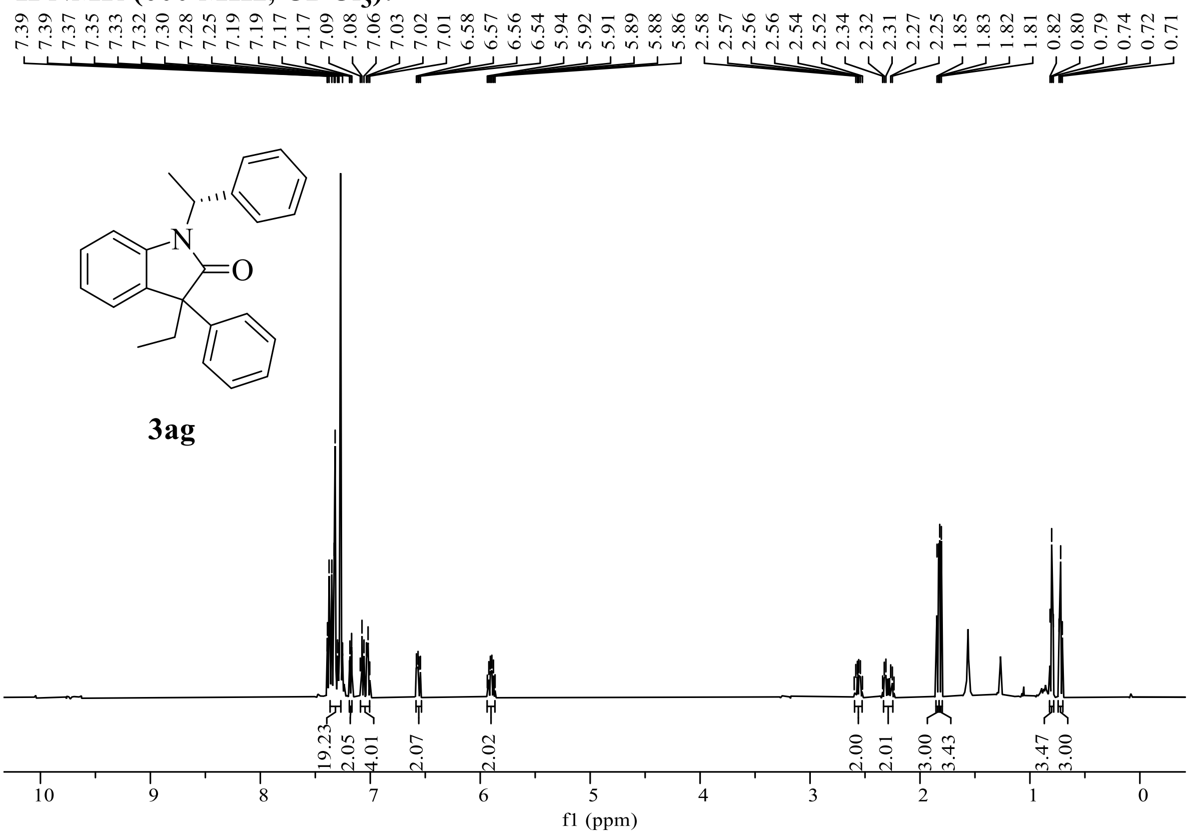

^{13}C NMR (151 MHz, $CDCl_3$):

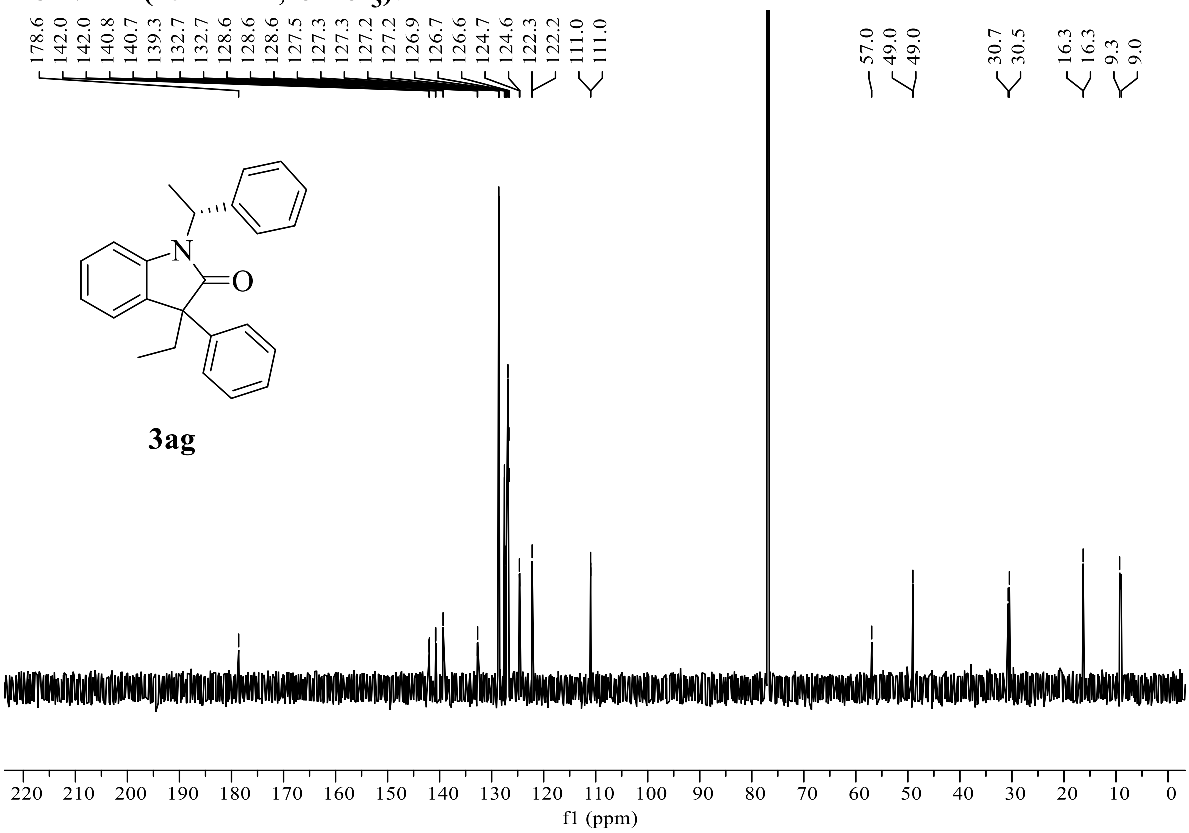

^{1}H NMR (600 MHz, $CDCl_3$):

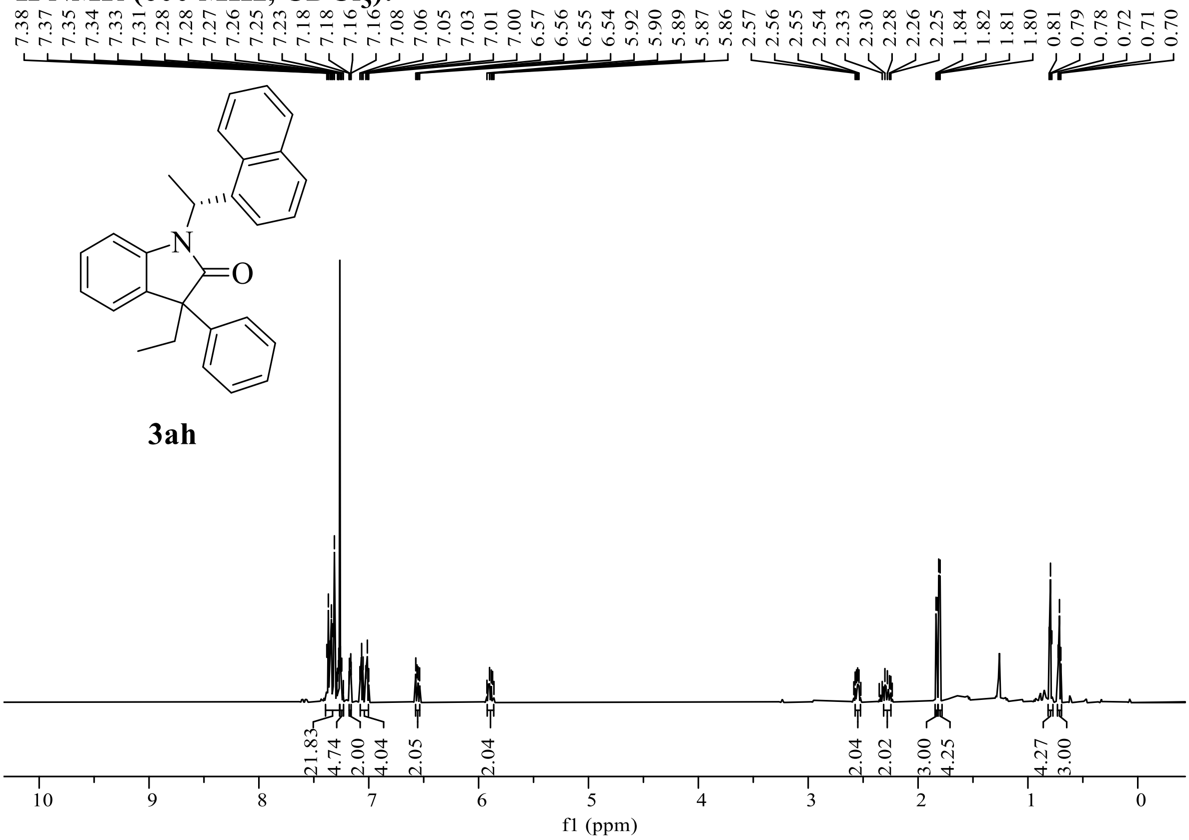

^{13}C NMR (151 MHz, $CDCl_3$):

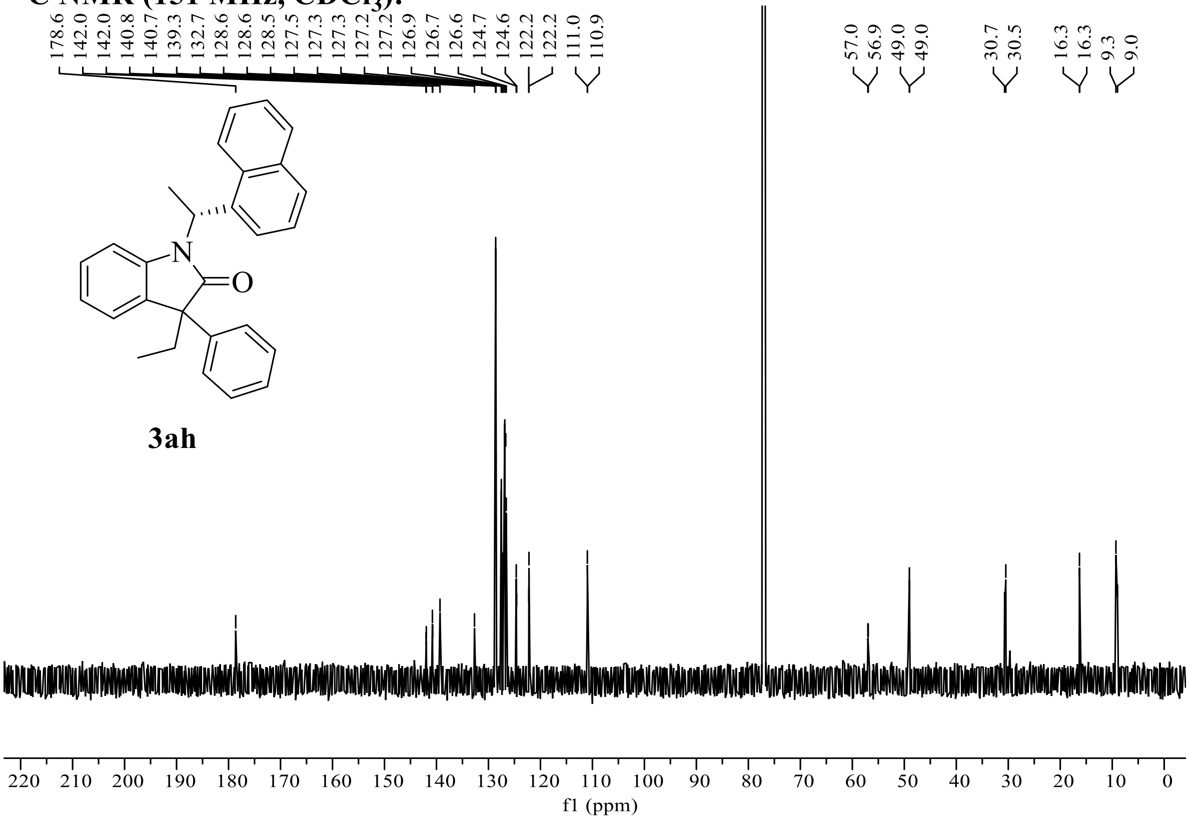

^{1}H NMR (300 MHz, $CDCl_3$):

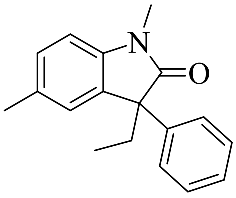

3ai

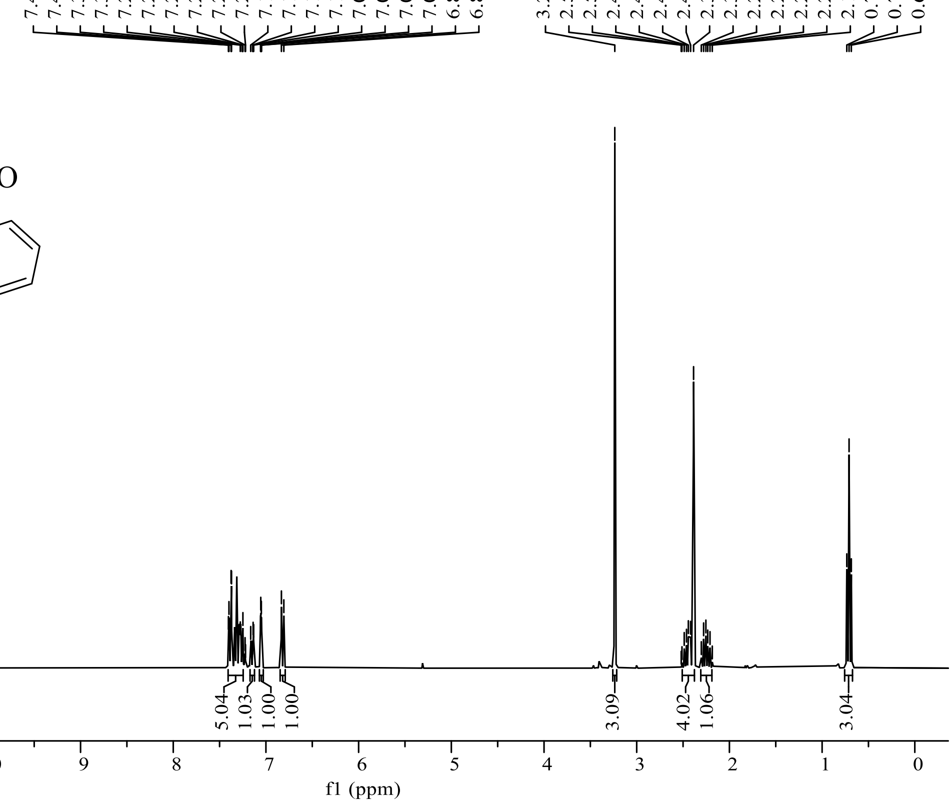

^{13}C NMR (75 MHz, $CDCl_3$):

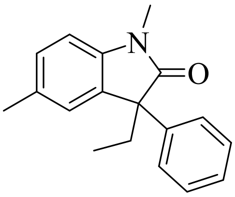

3ai

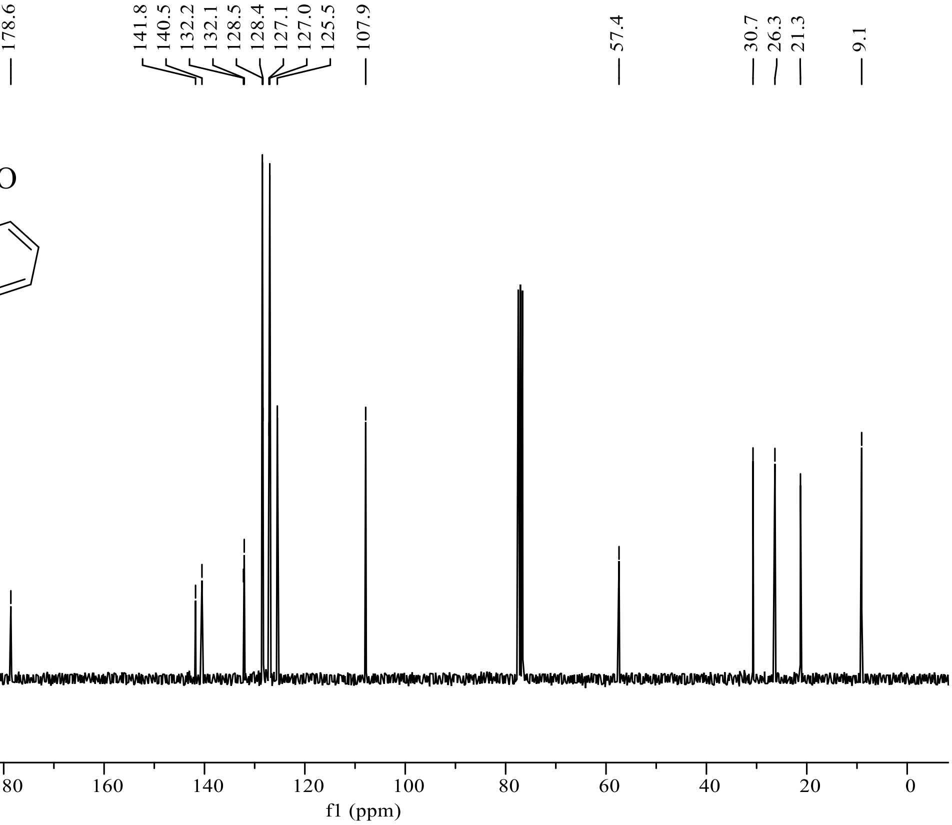

^{1}H NMR (600 MHz, $CDCl_3$):

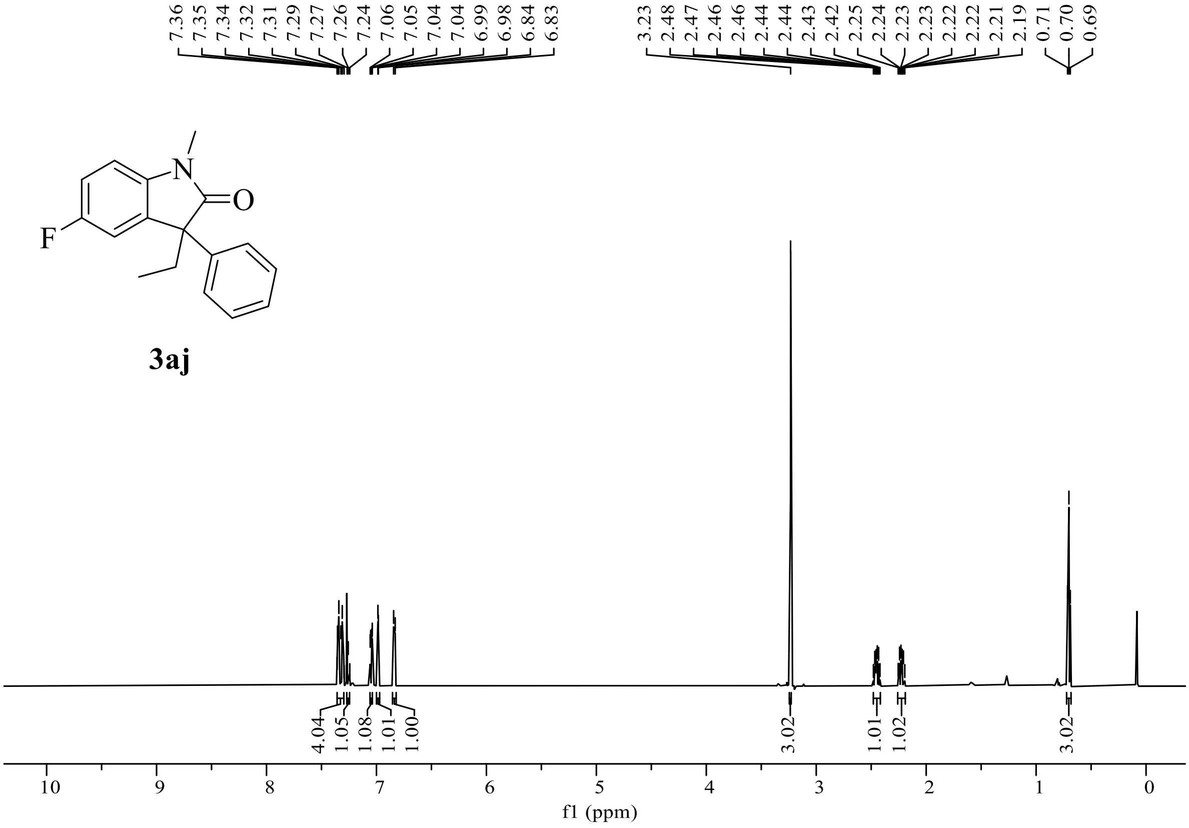

$^{13}C\{^{1}H,^{19}F\}$ NMR (151 MHz, $CDCl_3$):

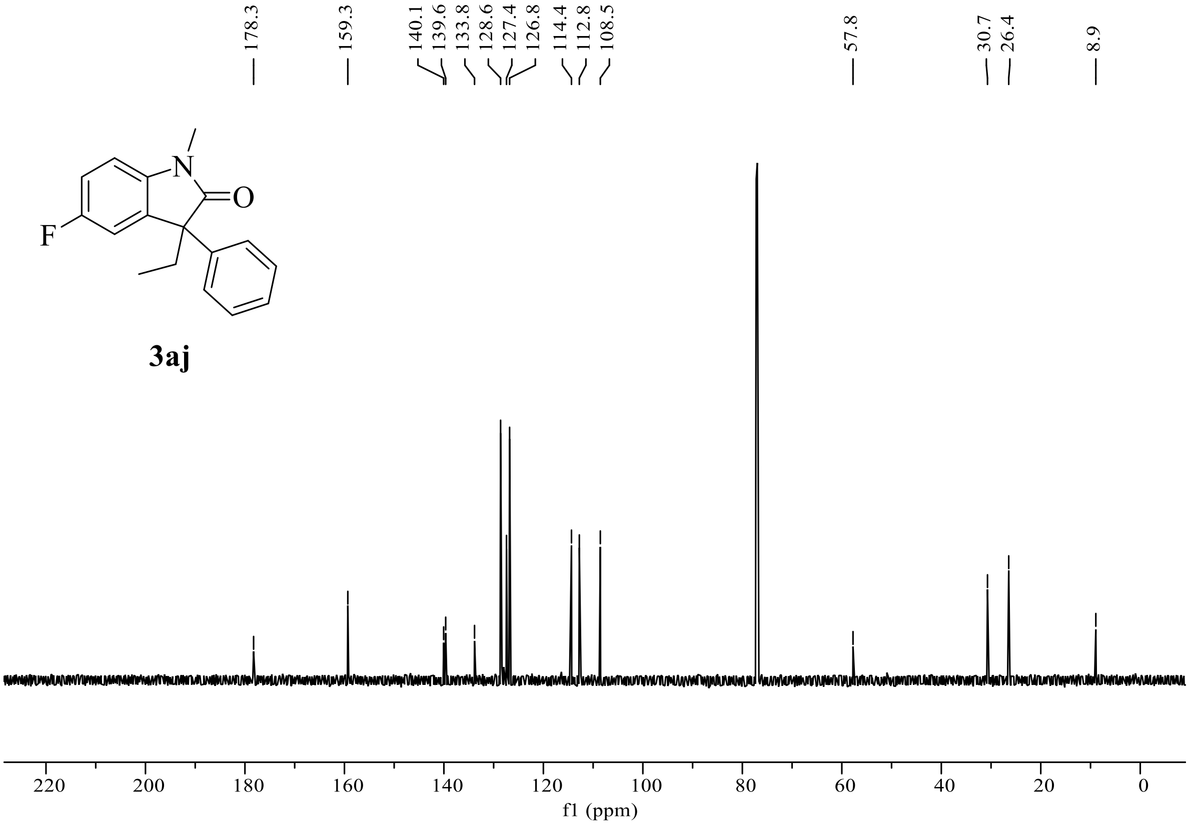

$^{19}F\{^{1}H,^{13}C\}$ NMR (282 MHz, $CDCl_3$):

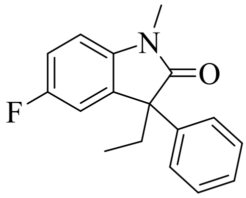

3aj

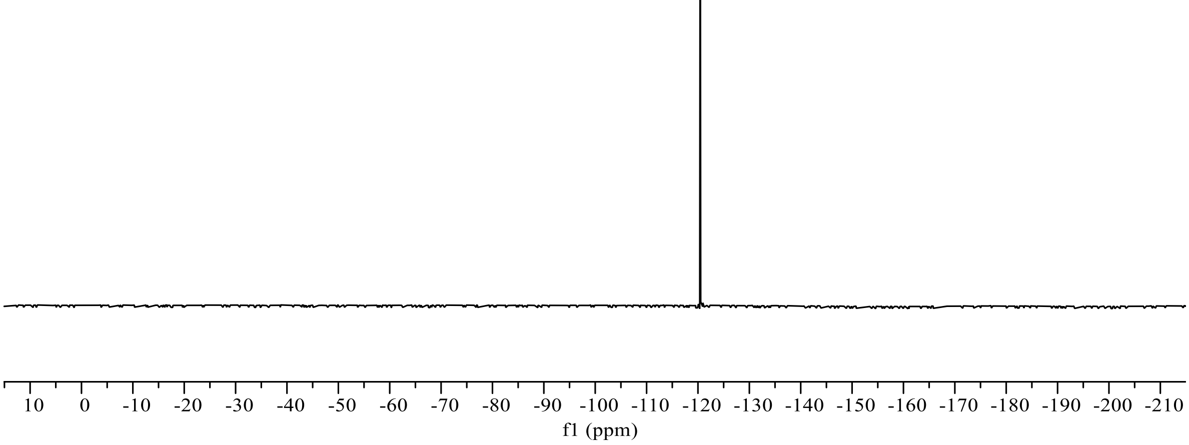

^{1}H NMR (300 MHz, $CDCl_3$):

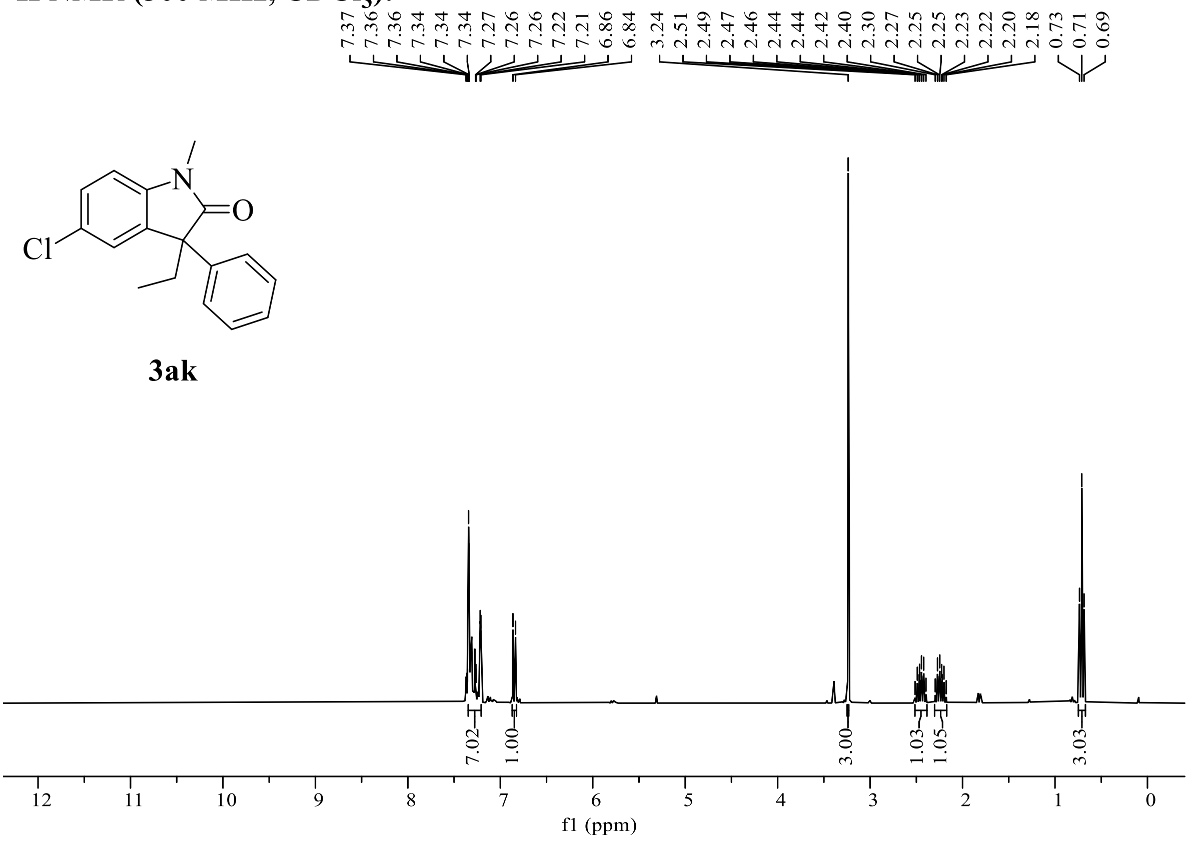

^{13}C NMR (75 MHz, $CDCl_3$):

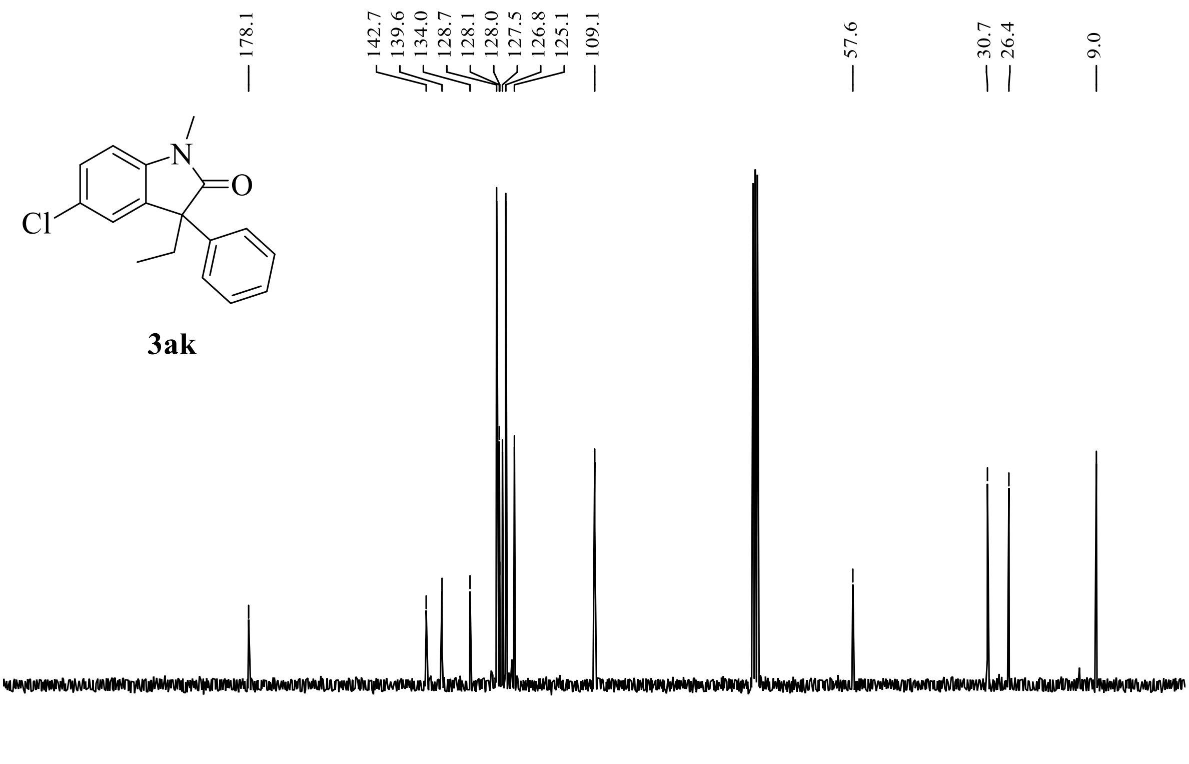

^{1}H NMR (300 MHz, $CDCl_3$):

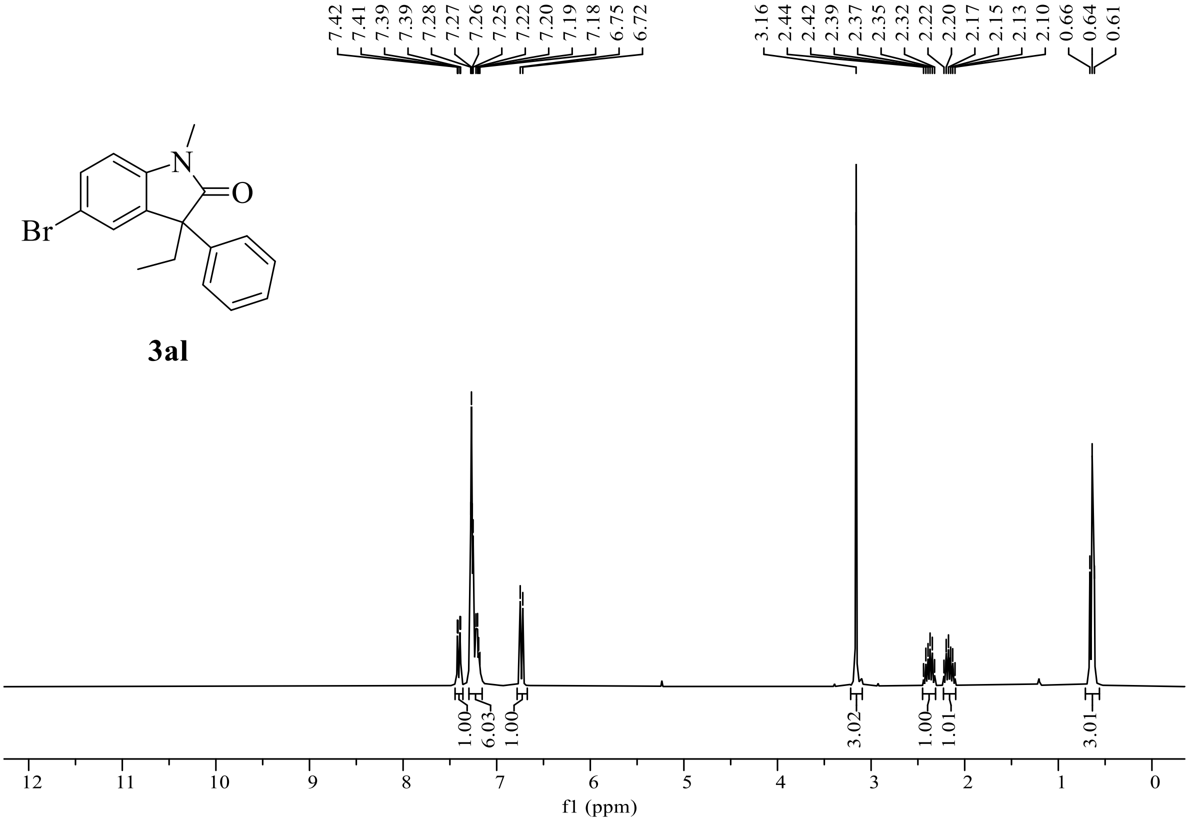

3al

^{13}C NMR (75 MHz, $CDCl_3$):

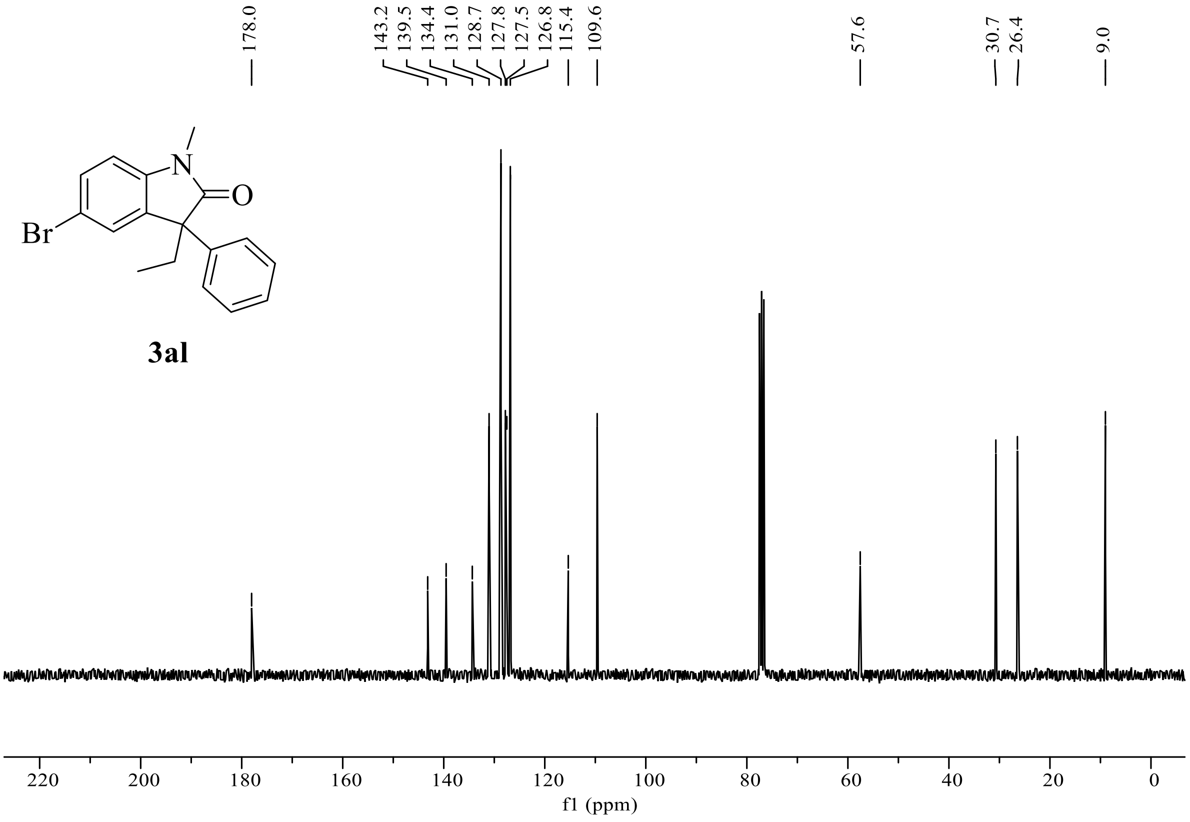

3al

^{1}H NMR (300 MHz, $CDCl_3$):

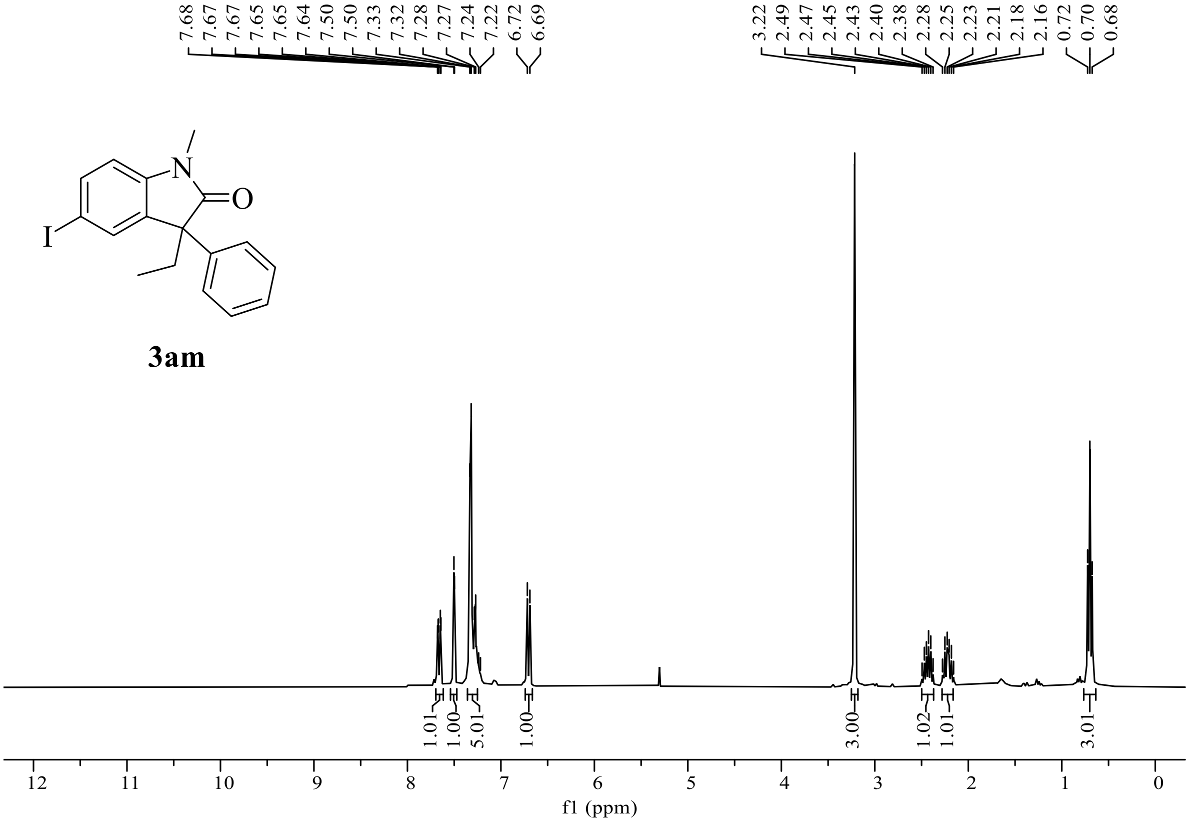

^{13}C NMR (75 MHz, $CDCl_3$):

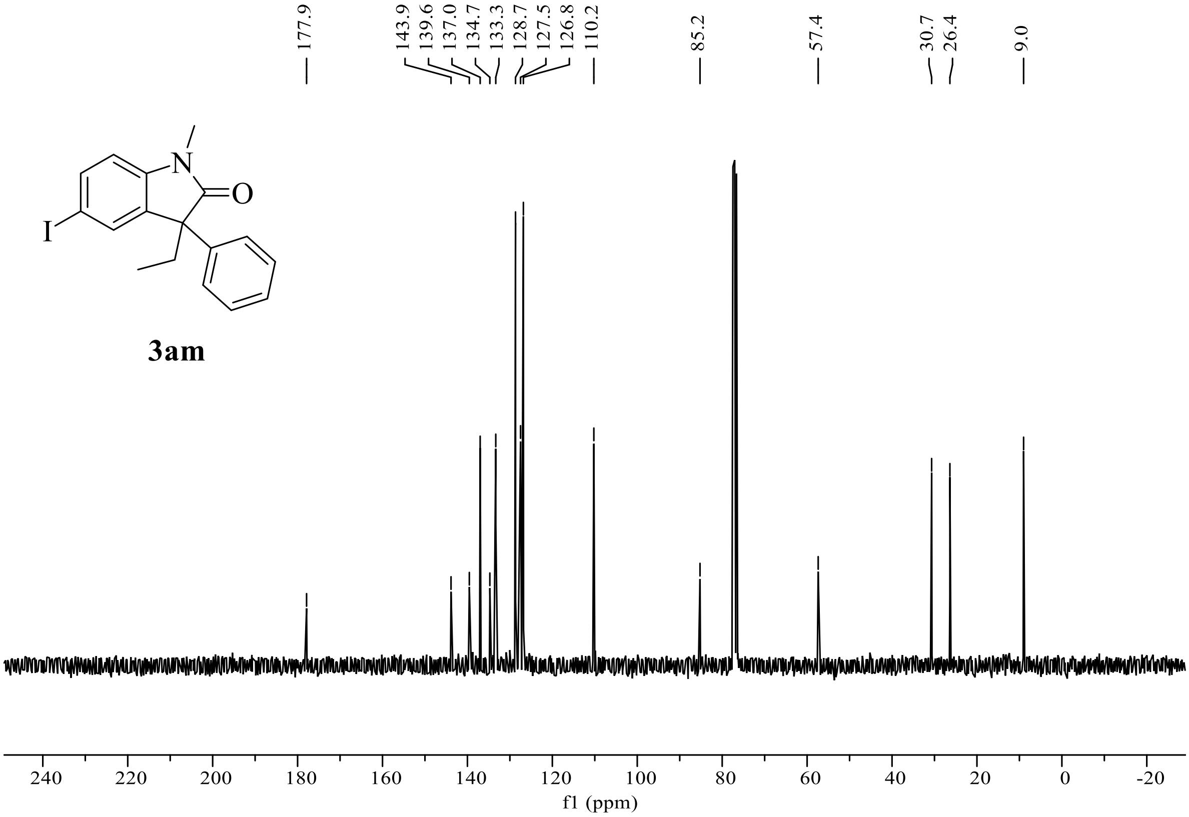

^{1}H NMR (300 MHz, $CDCl_3$):

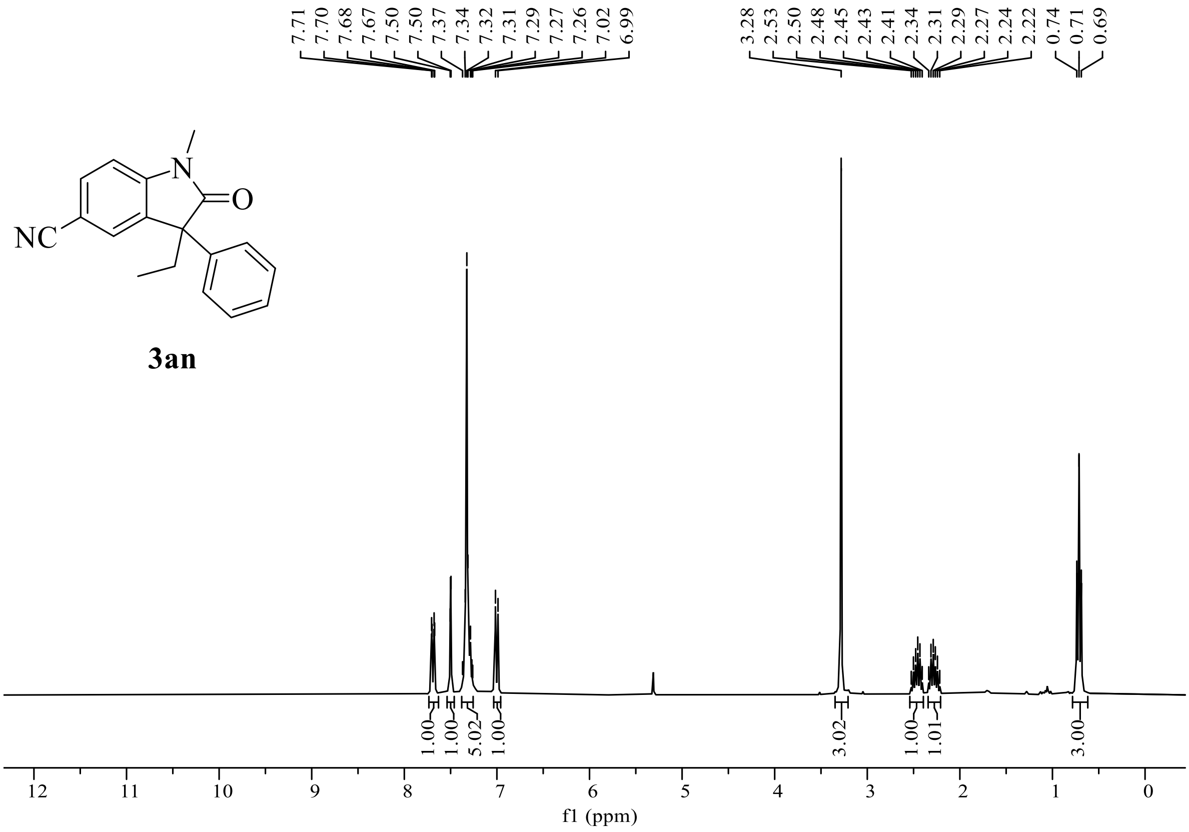

3an

^{13}C NMR (75 MHz, $CDCl_3$):

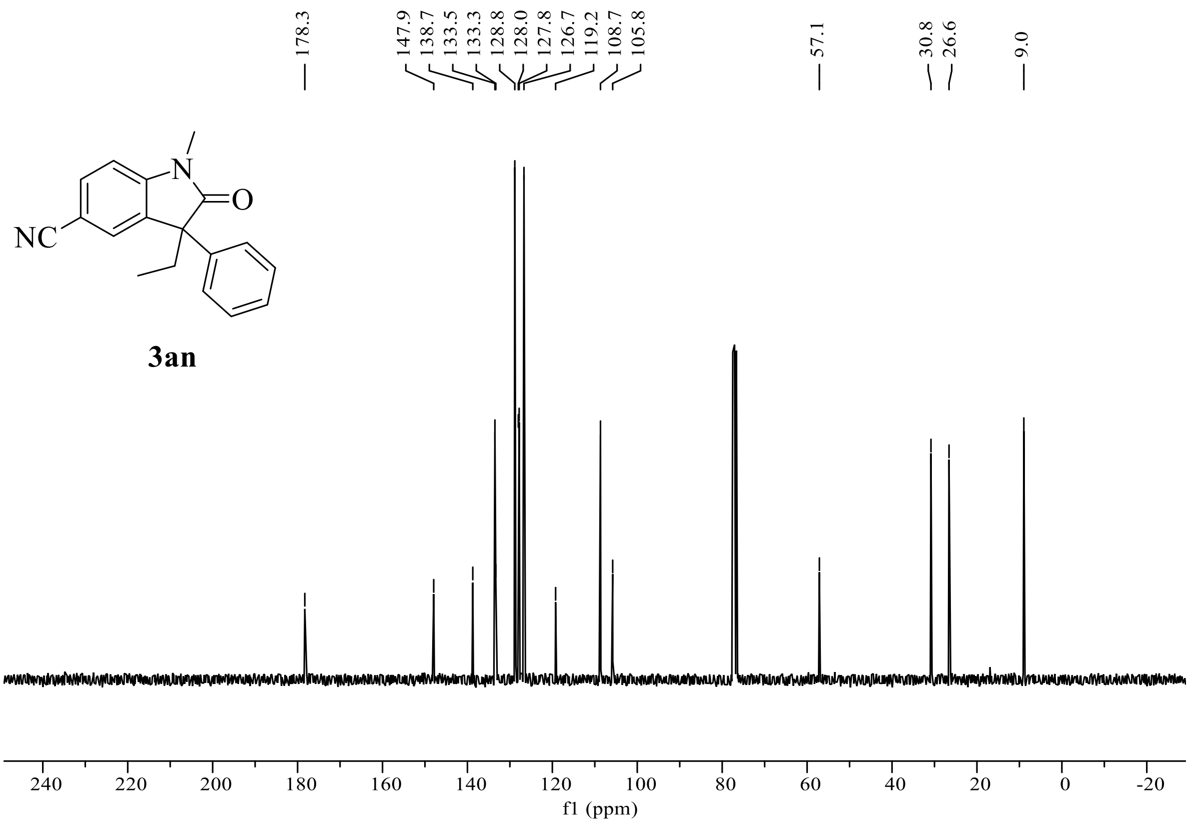

3an

^{1}H NMR (600 MHz, CDCl$_3$):

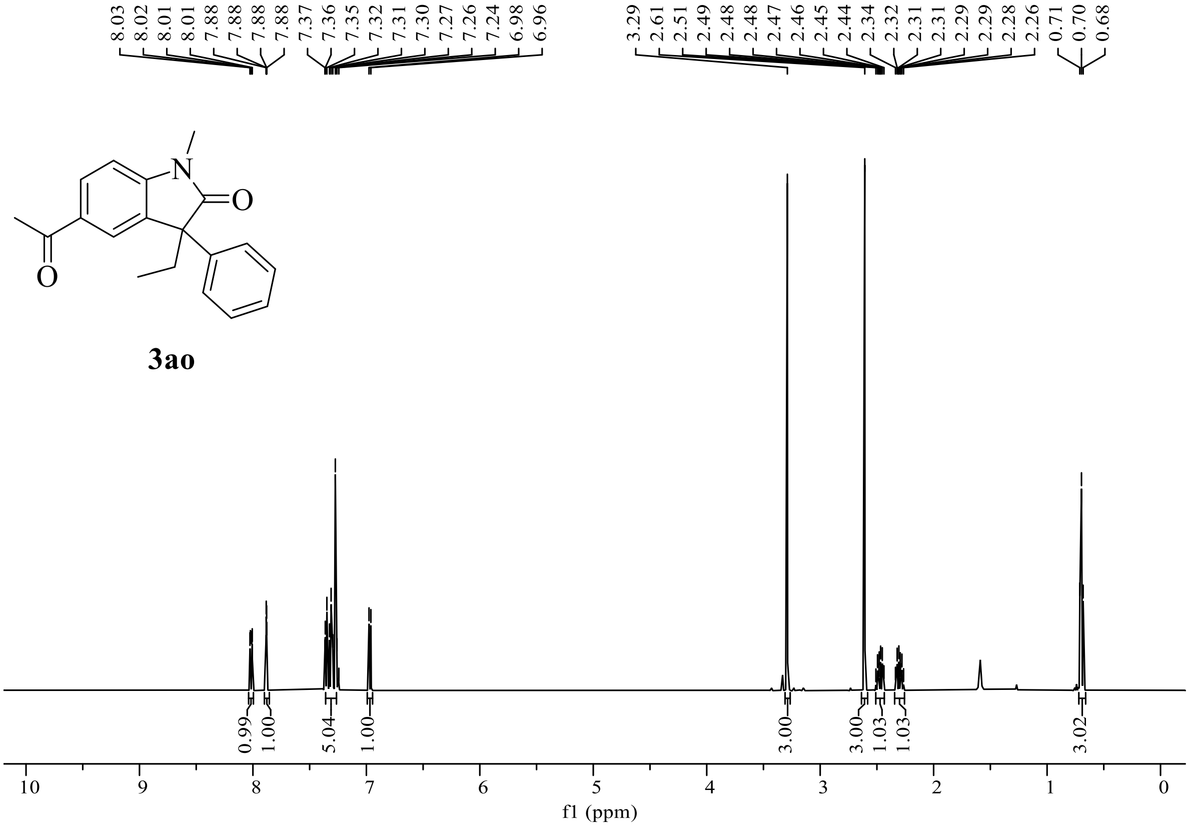

^{13}C NMR (151 MHz, CDCl$_3$):

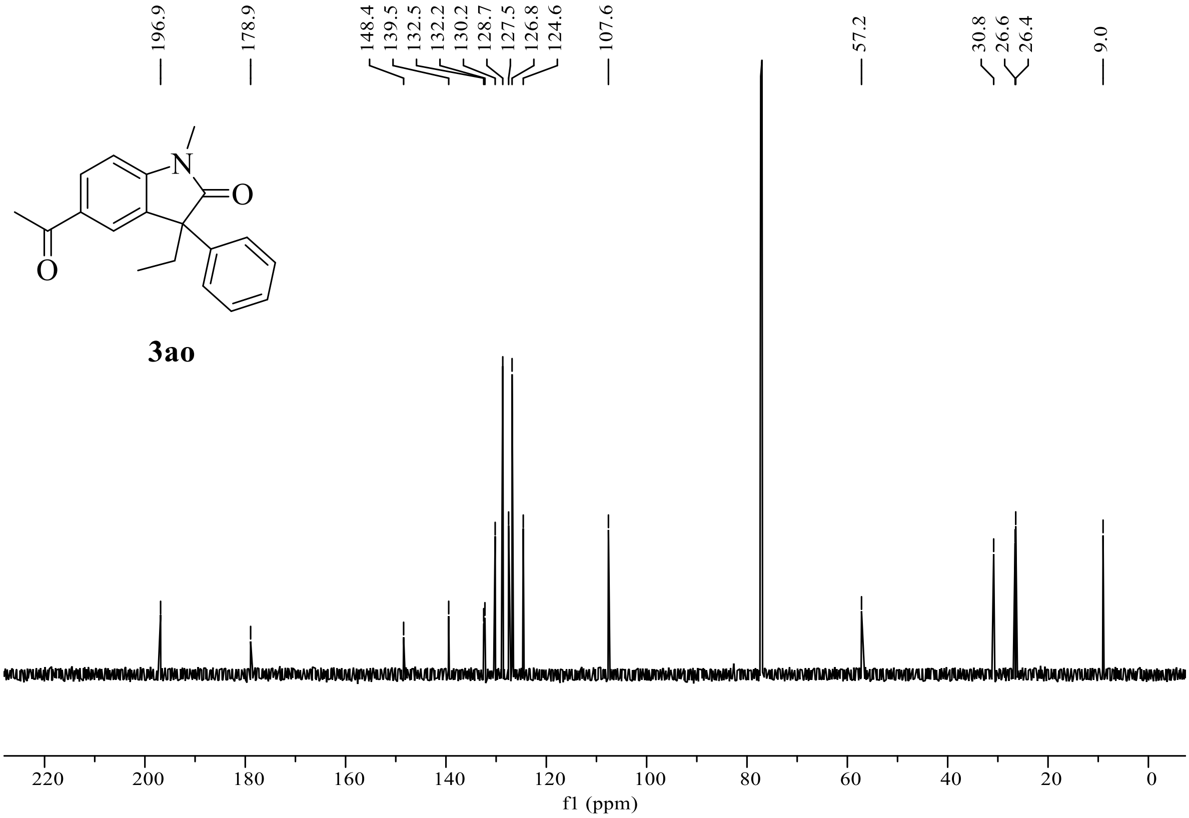

^{1}H NMR (300 MHz, $CDCl_3$):

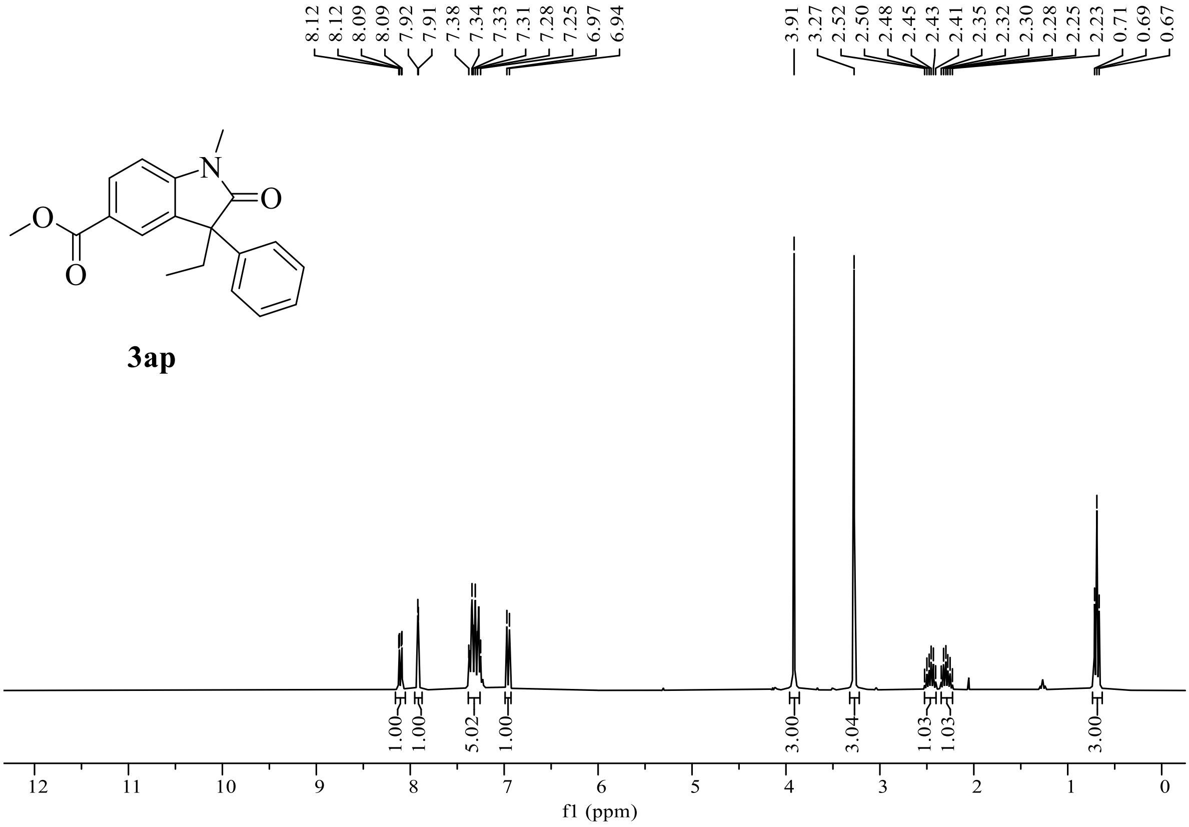

3ap

^{13}C NMR (75 MHz, $CDCl_3$):

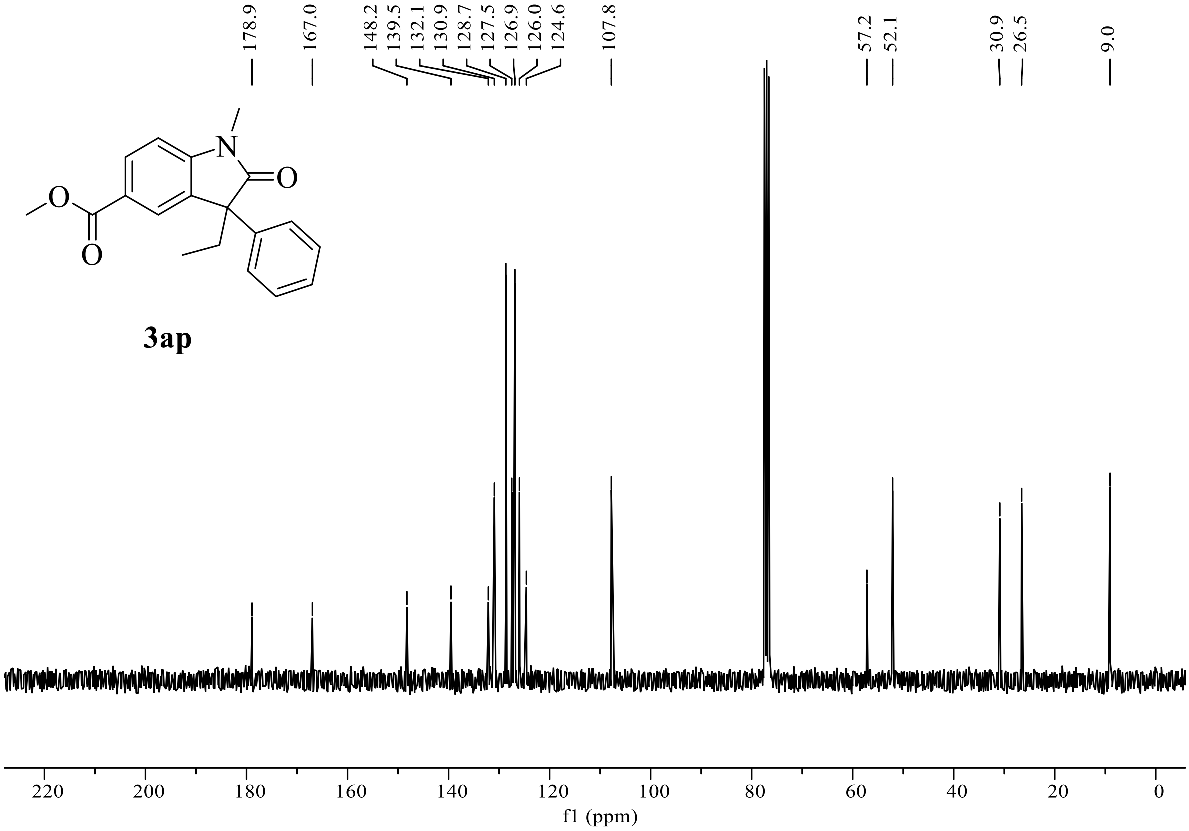

3ap

^{1}H NMR (300 MHz, $CDCl_3$):

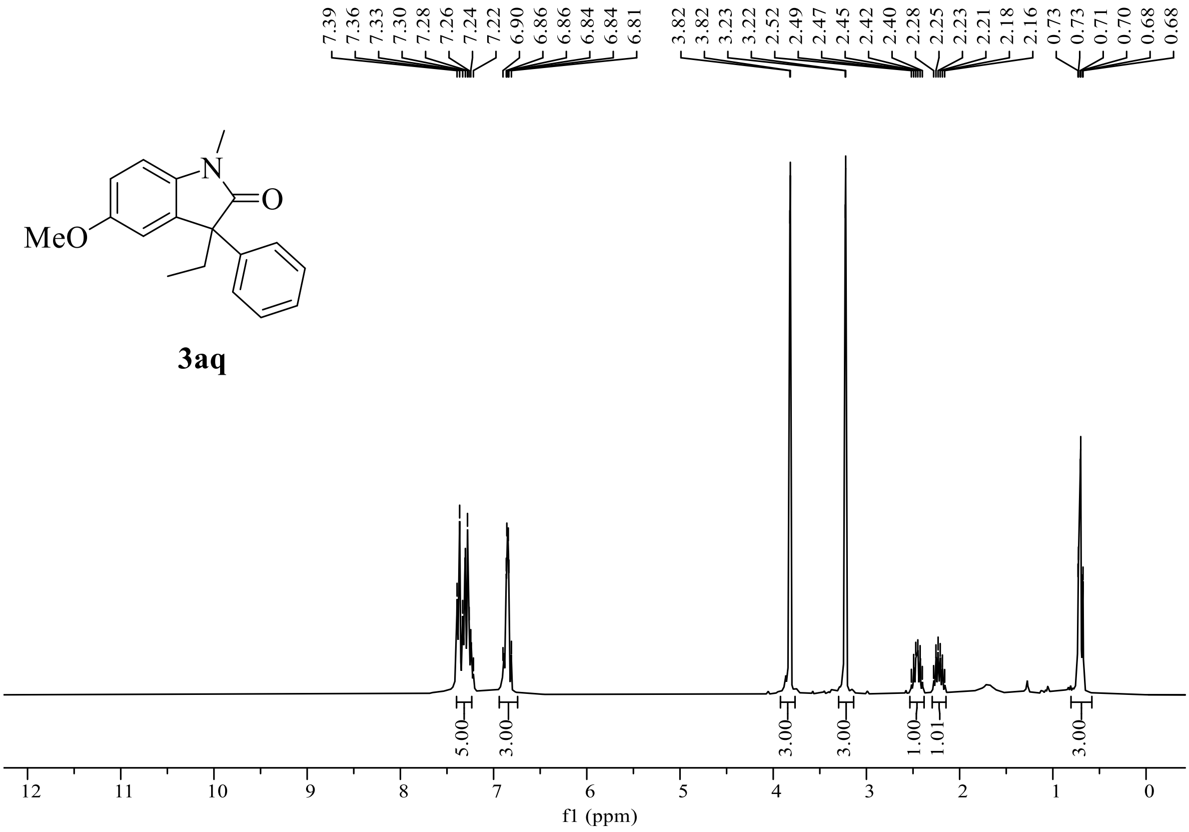

^{13}C NMR (75 MHz, $CDCl_3$):

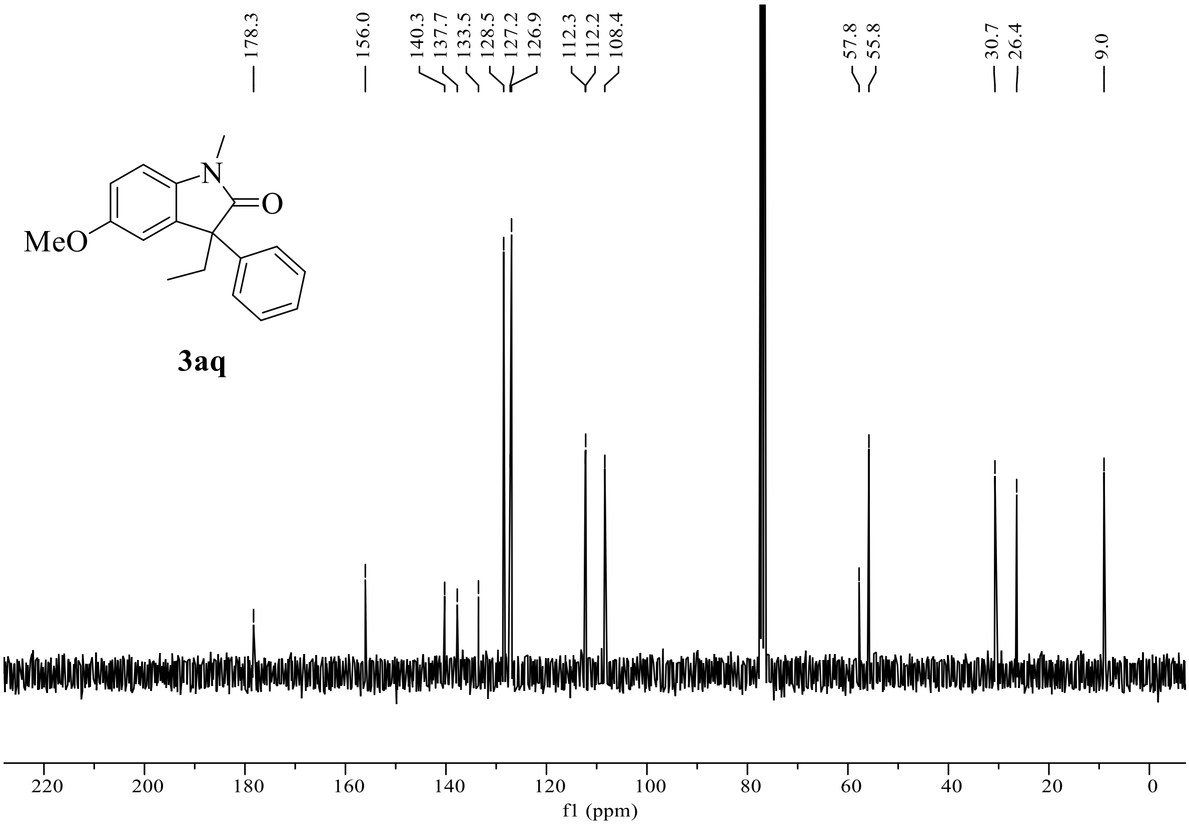

^{1}H NMR (300 MHz, $CDCl_3$):

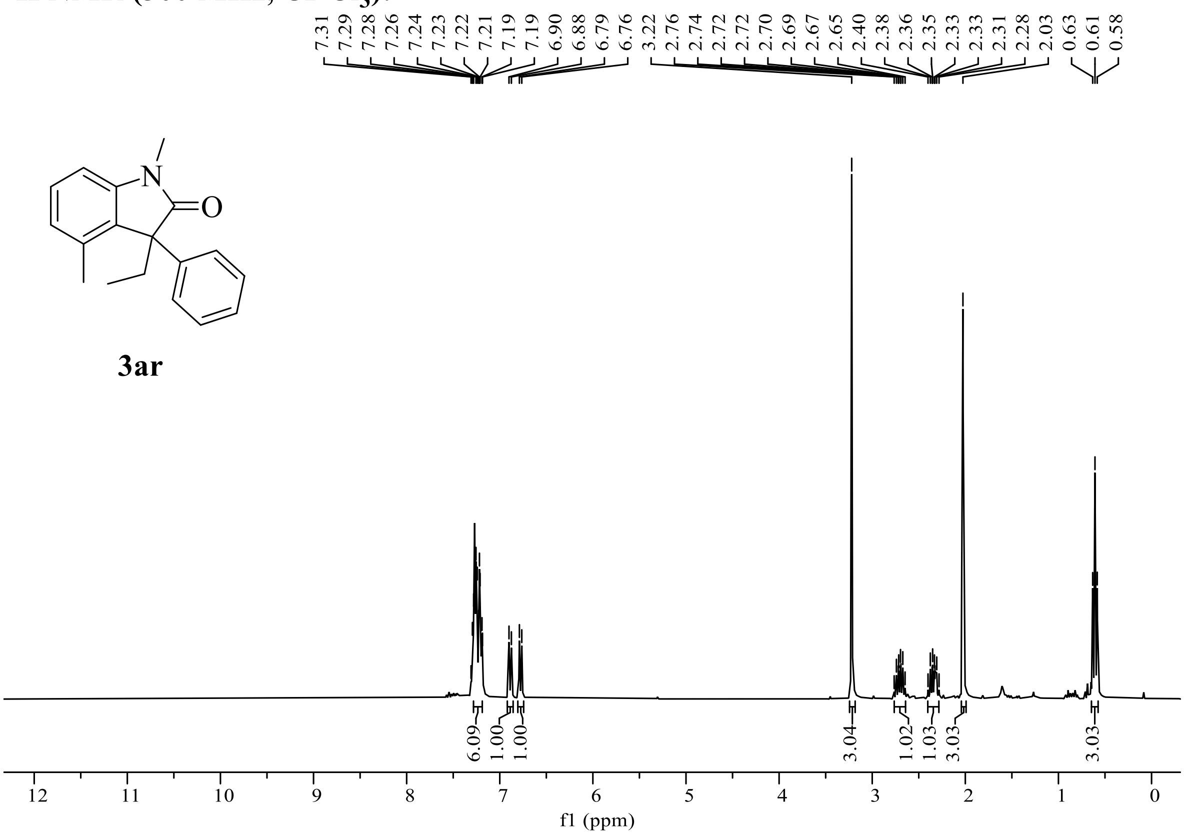

^{13}C NMR (151 MHz, $CDCl_3$):

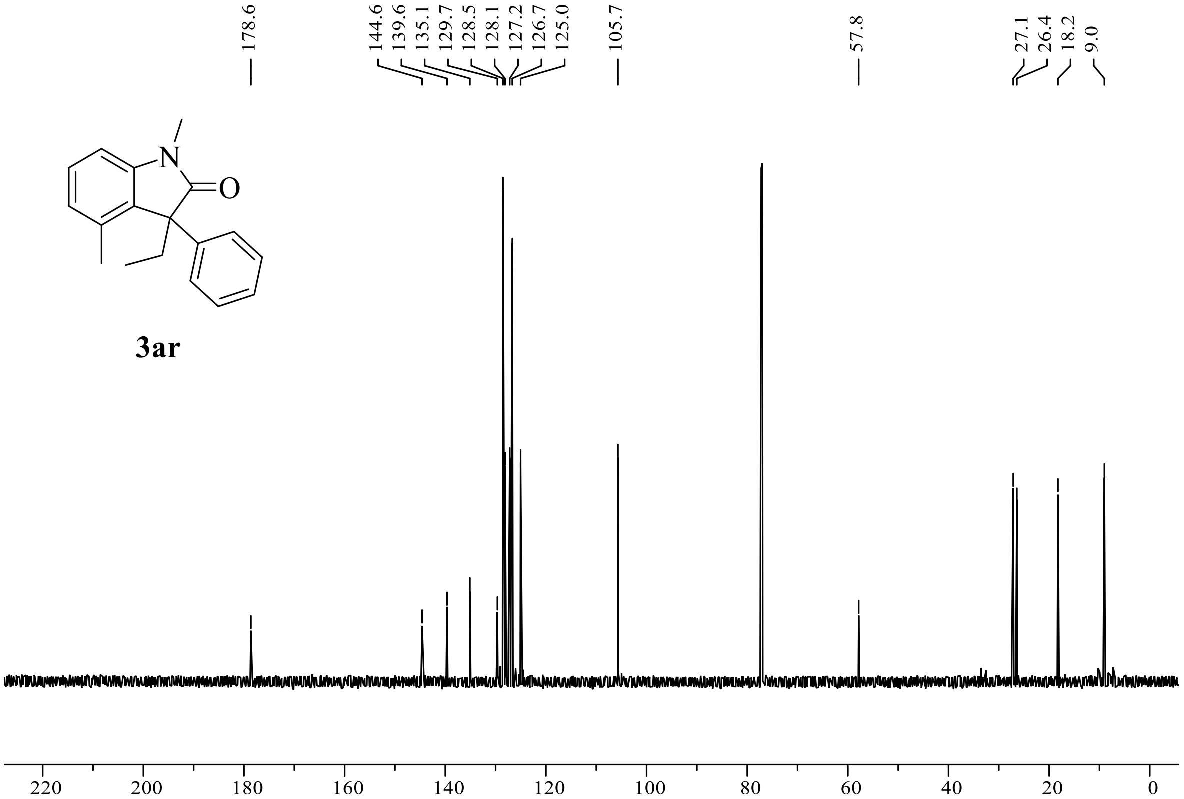

2D-COSY NMR (600 MHz, $CDCl_3$)

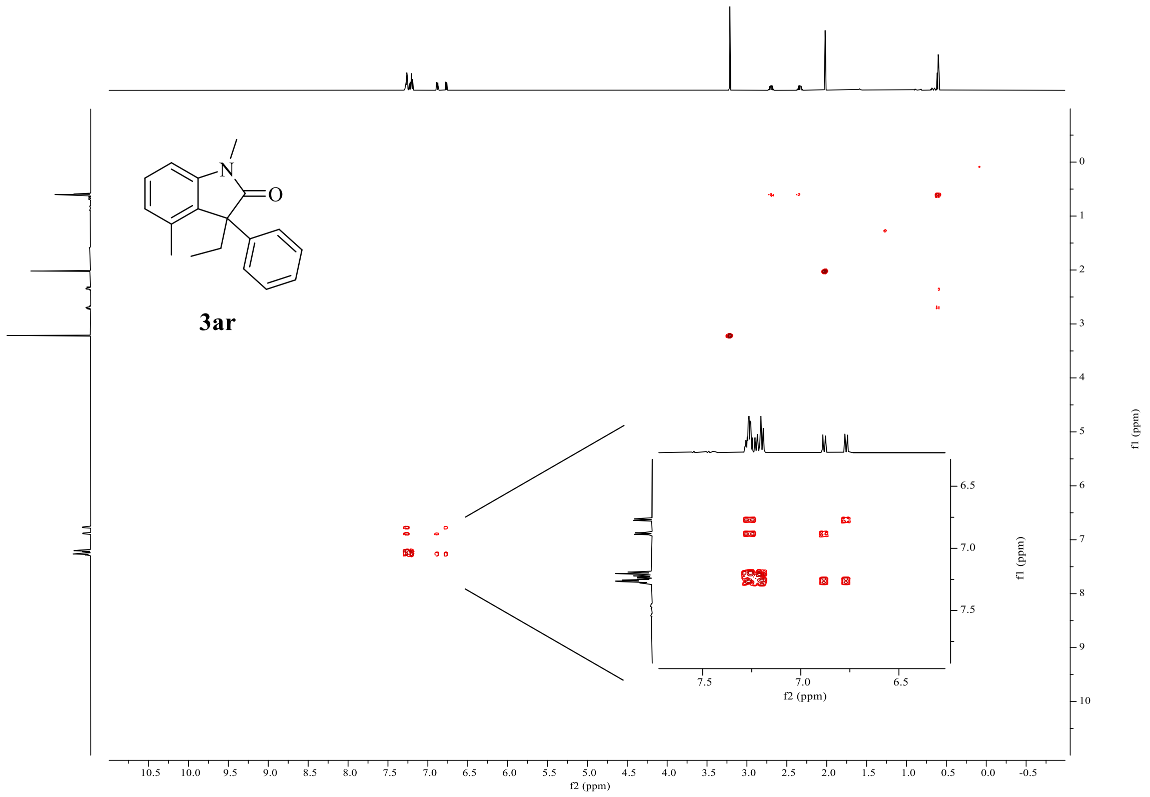

^{1}H NMR (300 MHz, $CDCl_3$):

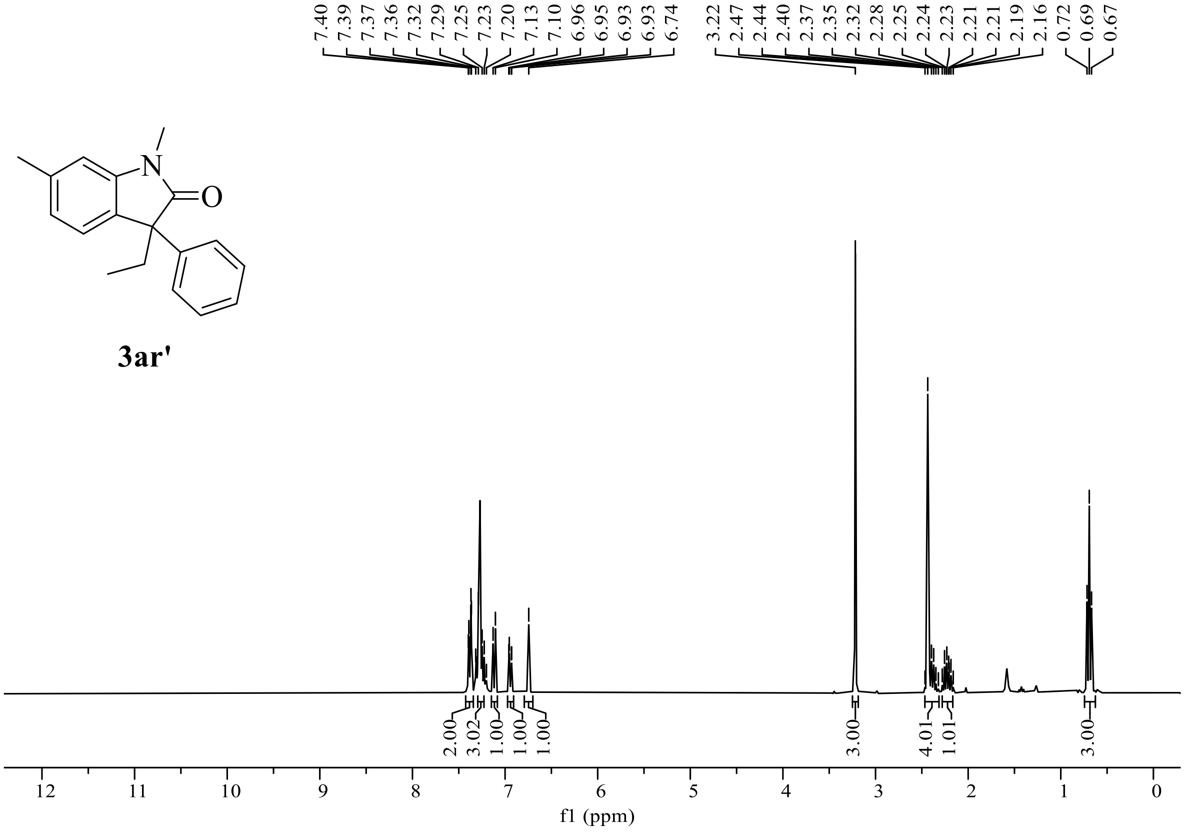

^{13}C NMR (151 MHz, $CDCl_3$):

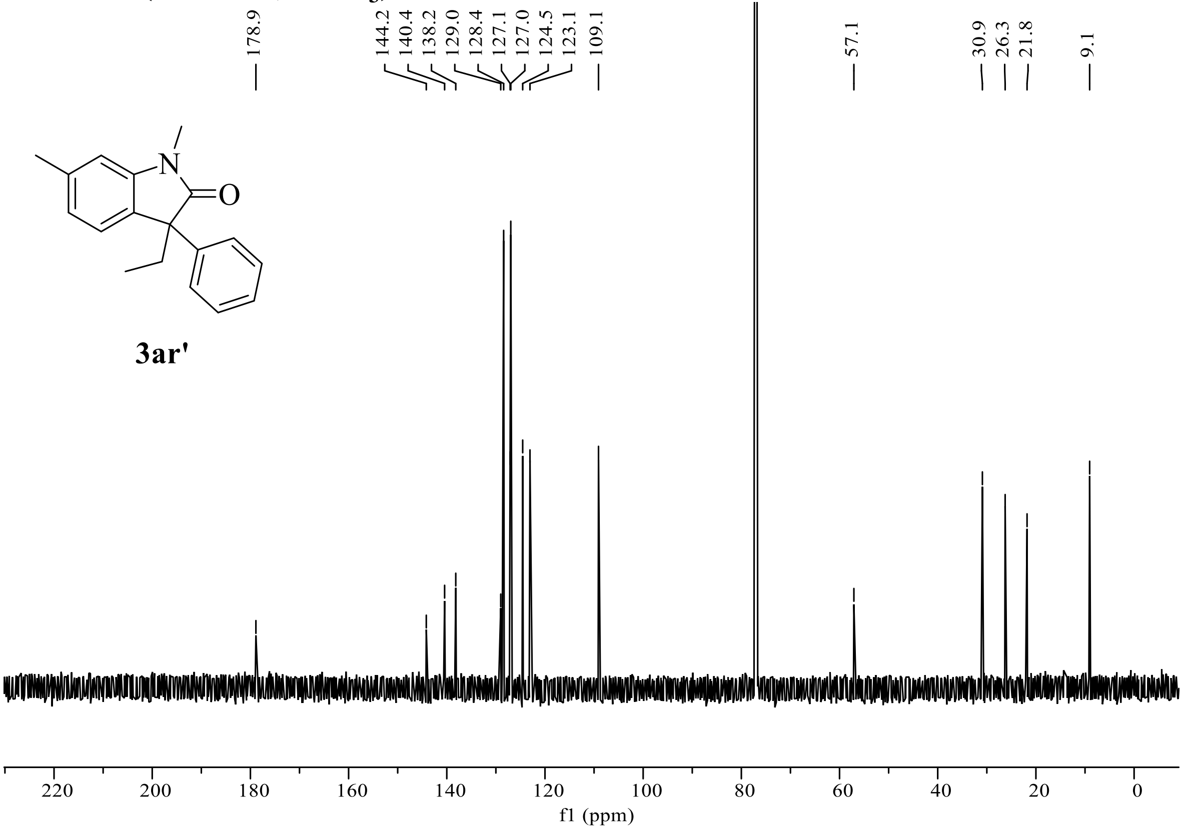

2D-COSY NMR (600 MHz, $CDCl_3$)

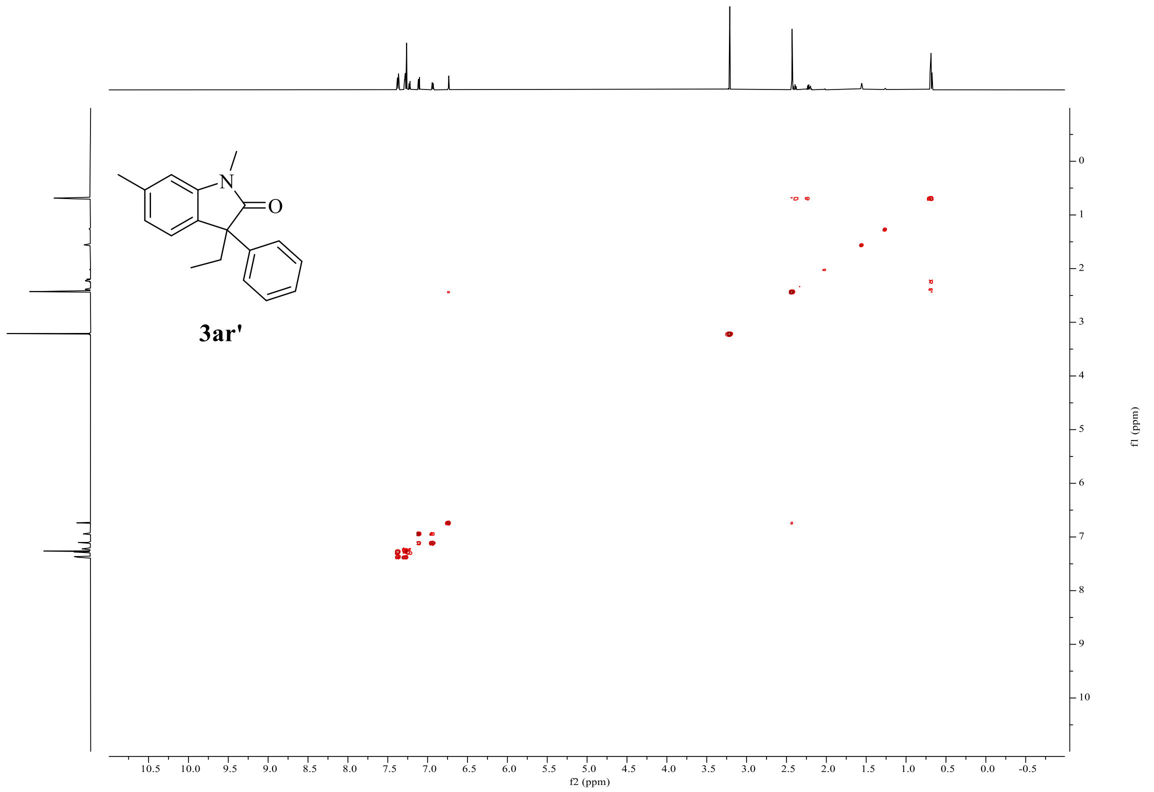

^{1}H NMR (300 MHz, $CDCl_3$):

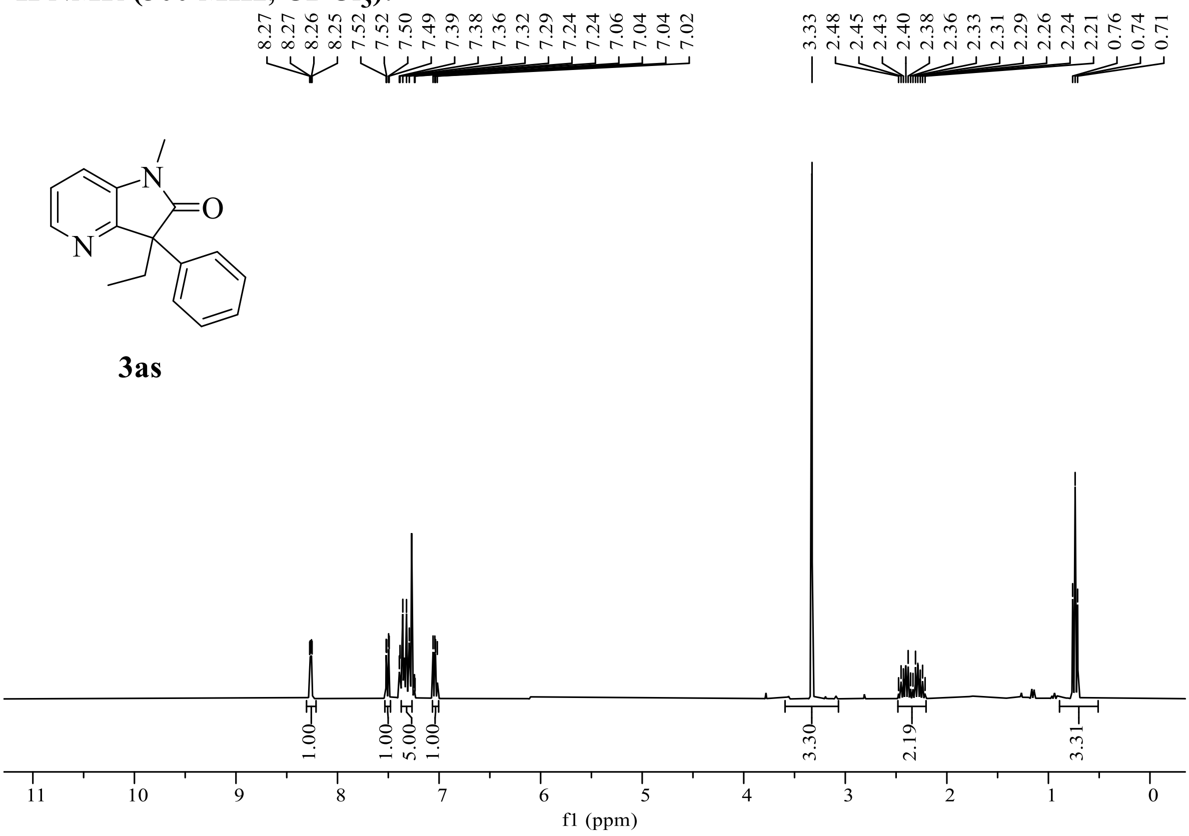

^{13}C NMR (75 MHz, $CDCl_3$):

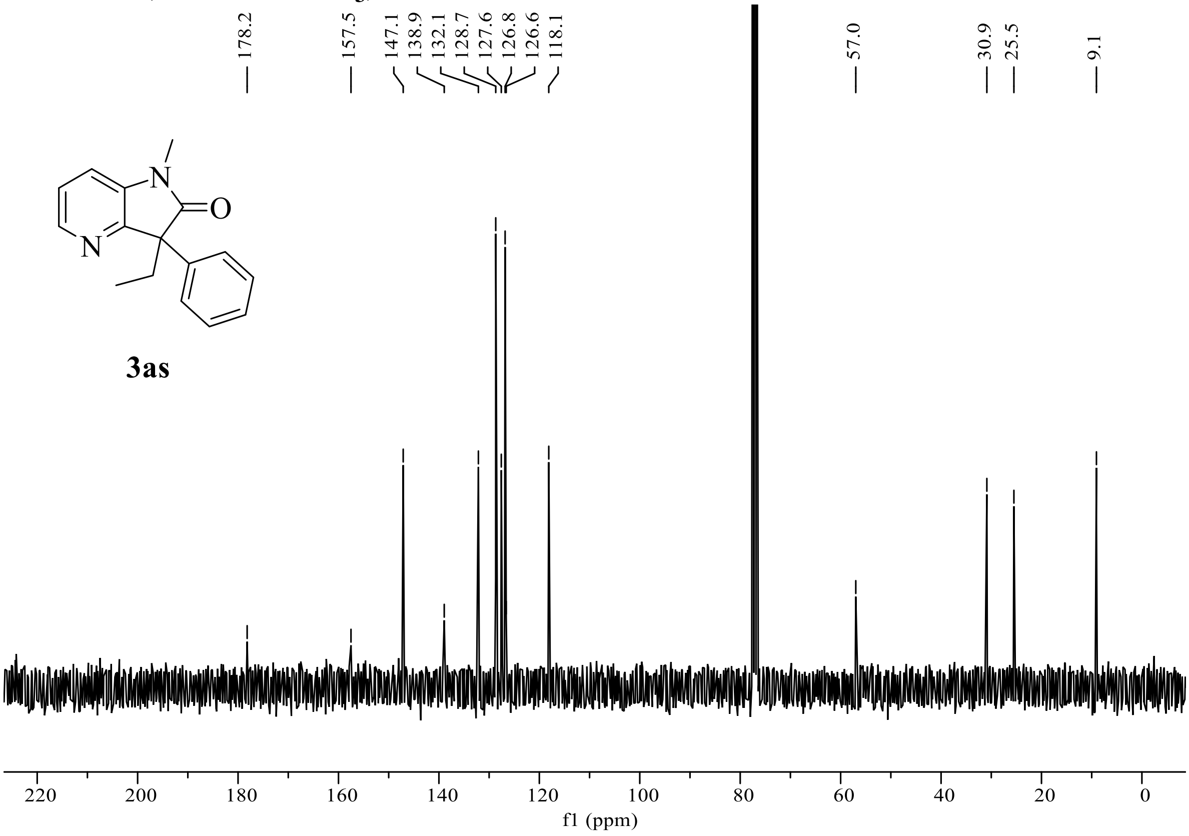

^{1}H NMR (300 MHz, $CDCl_3$):

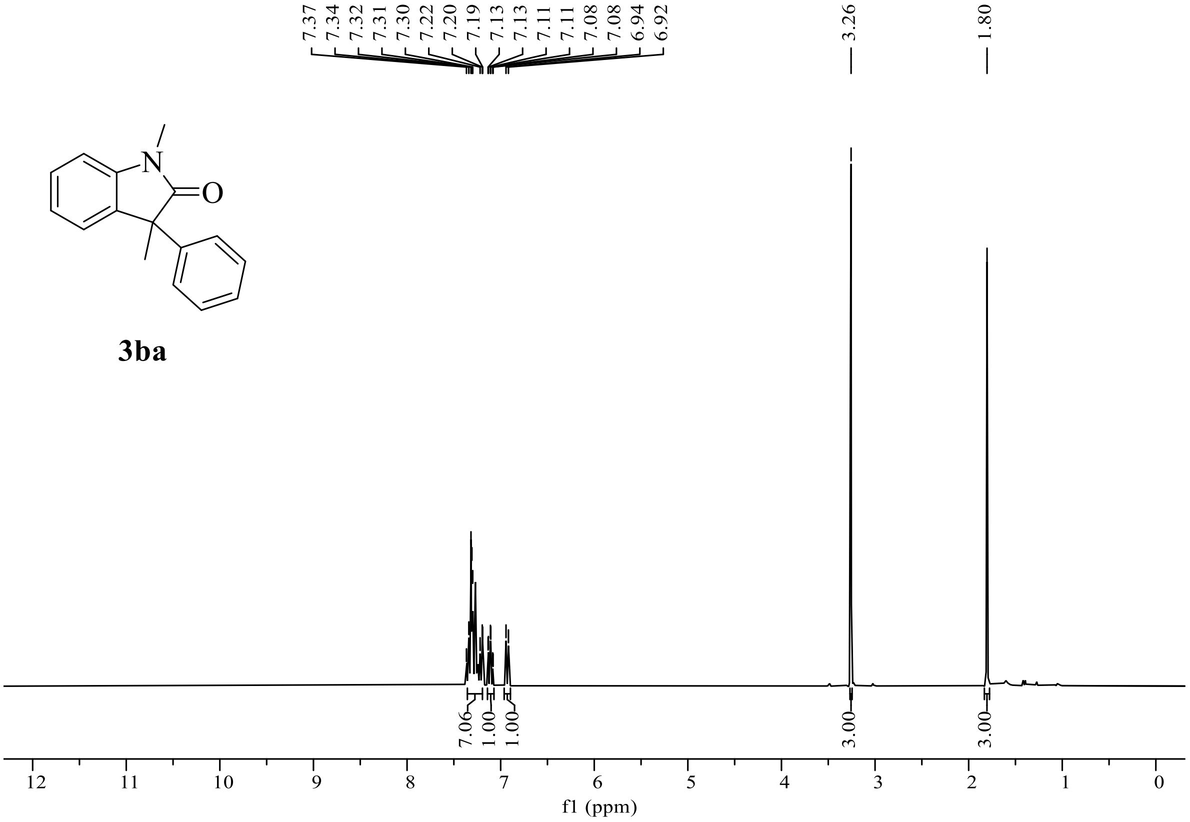

^{13}C NMR (75 MHz, $CDCl_3$):

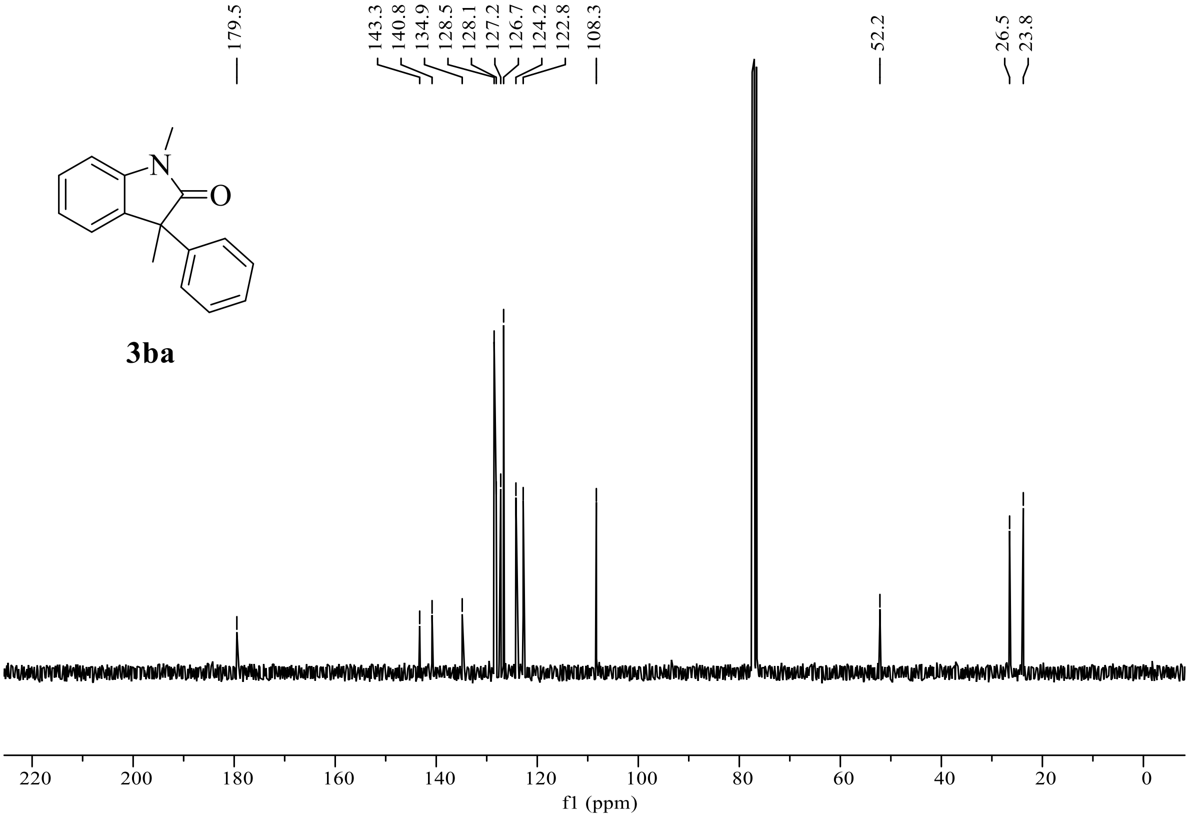

^{1}H NMR (300 MHz, $CDCl_3$):

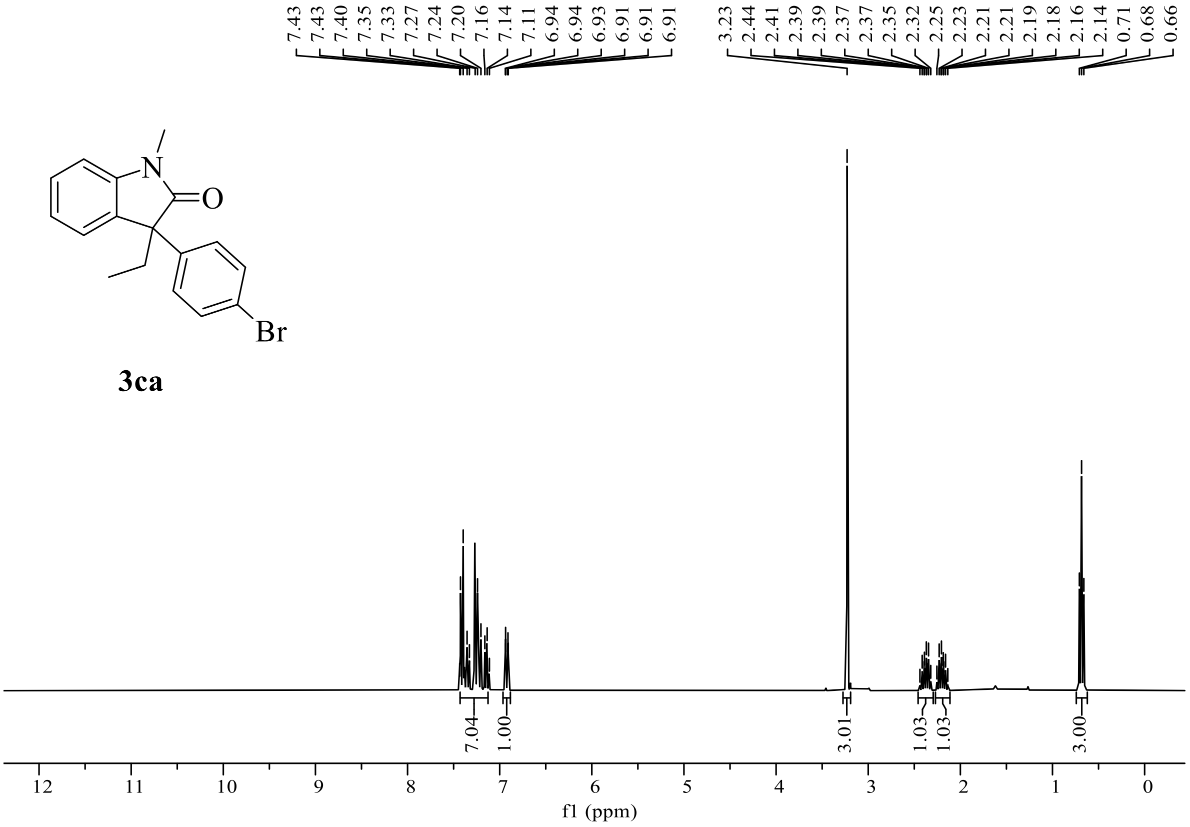

^{13}C NMR (75 MHz, $CDCl_3$):

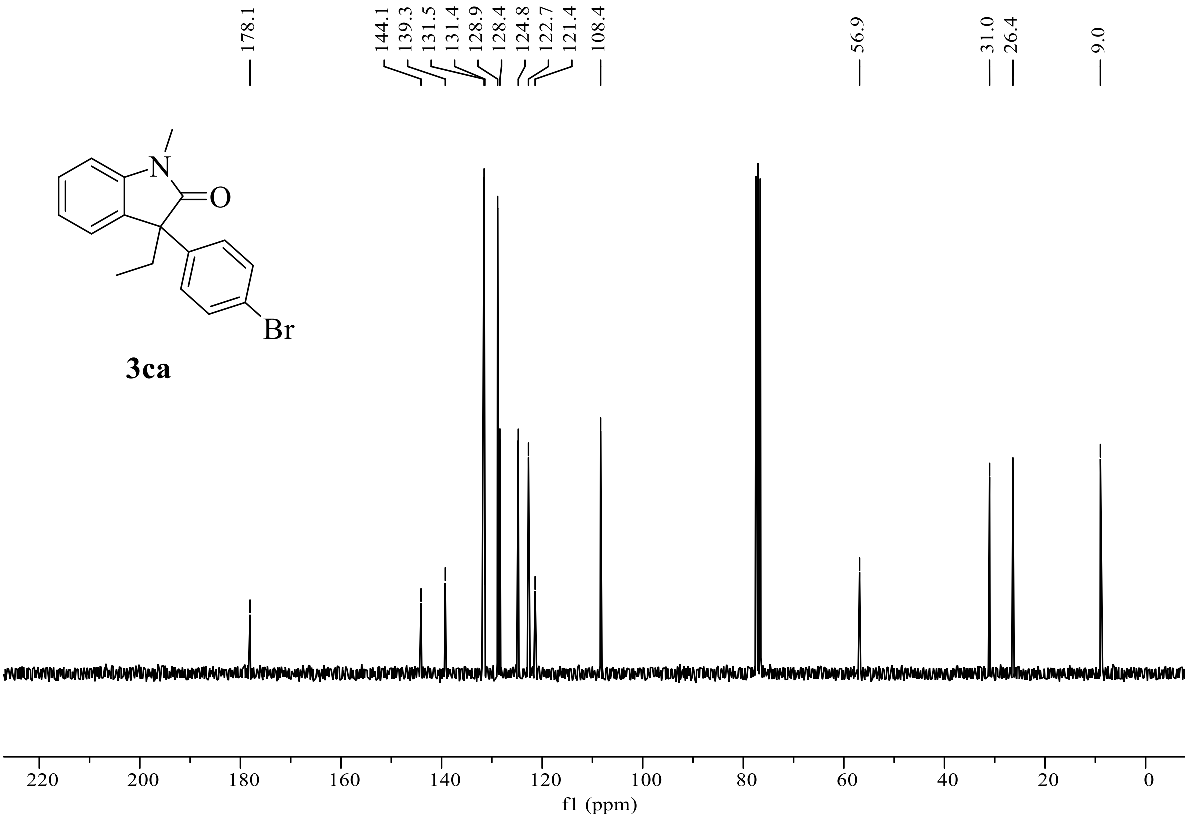

^{1}H NMR (300 MHz, $CDCl_3$):

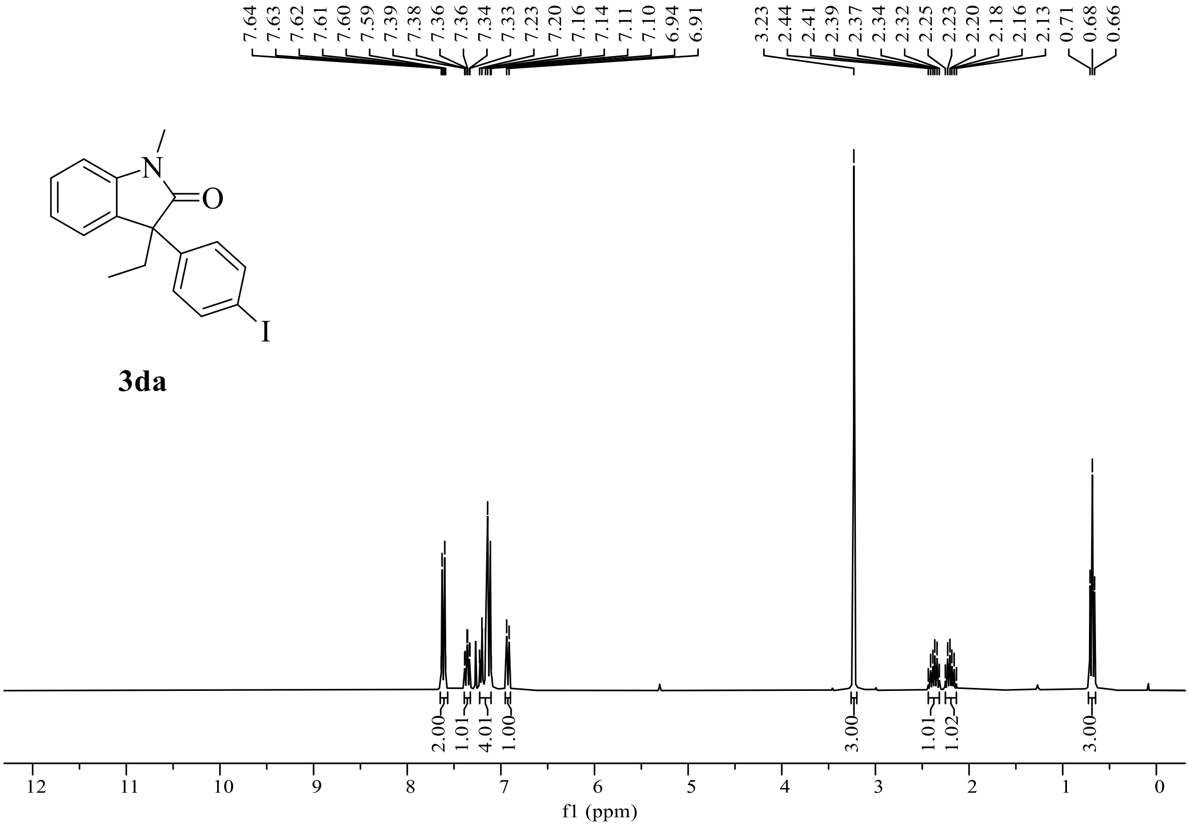

^{13}C NMR (75 MHz, $CDCl_3$):

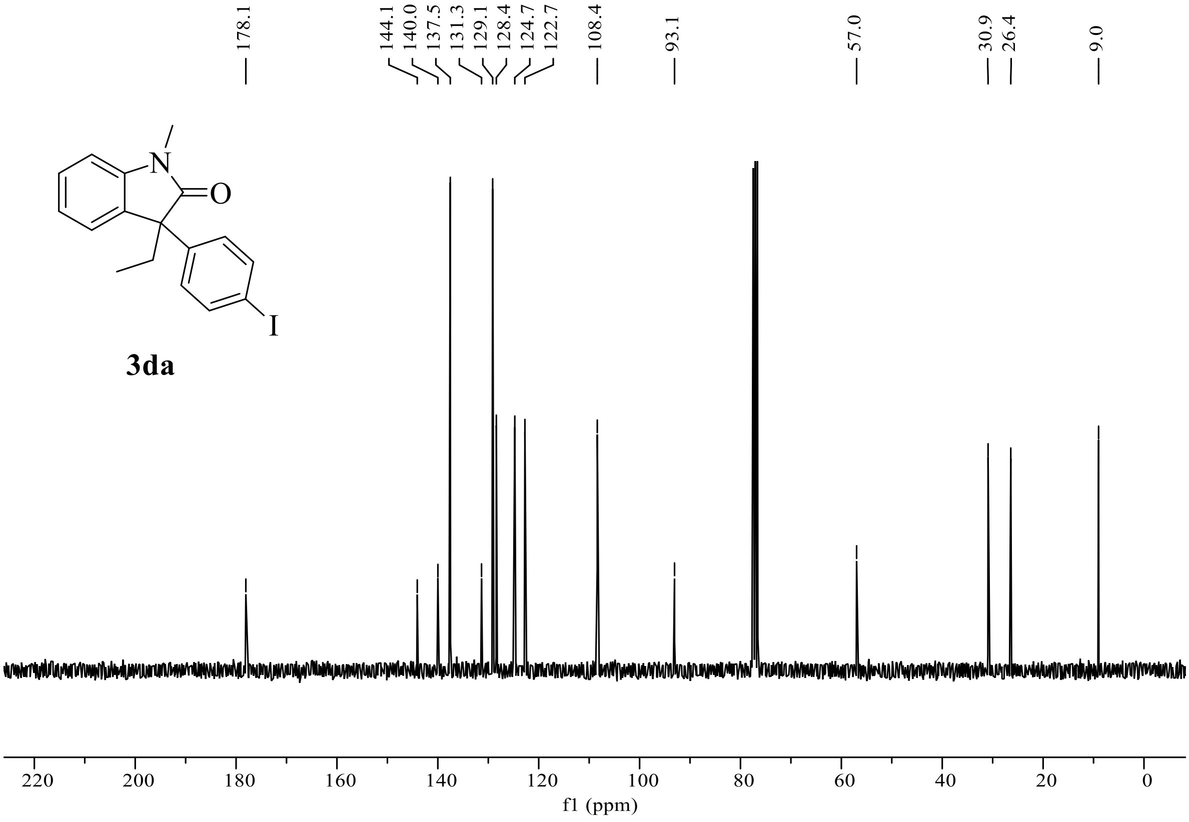

^{1}H NMR (300 MHz, $CDCl_3$):

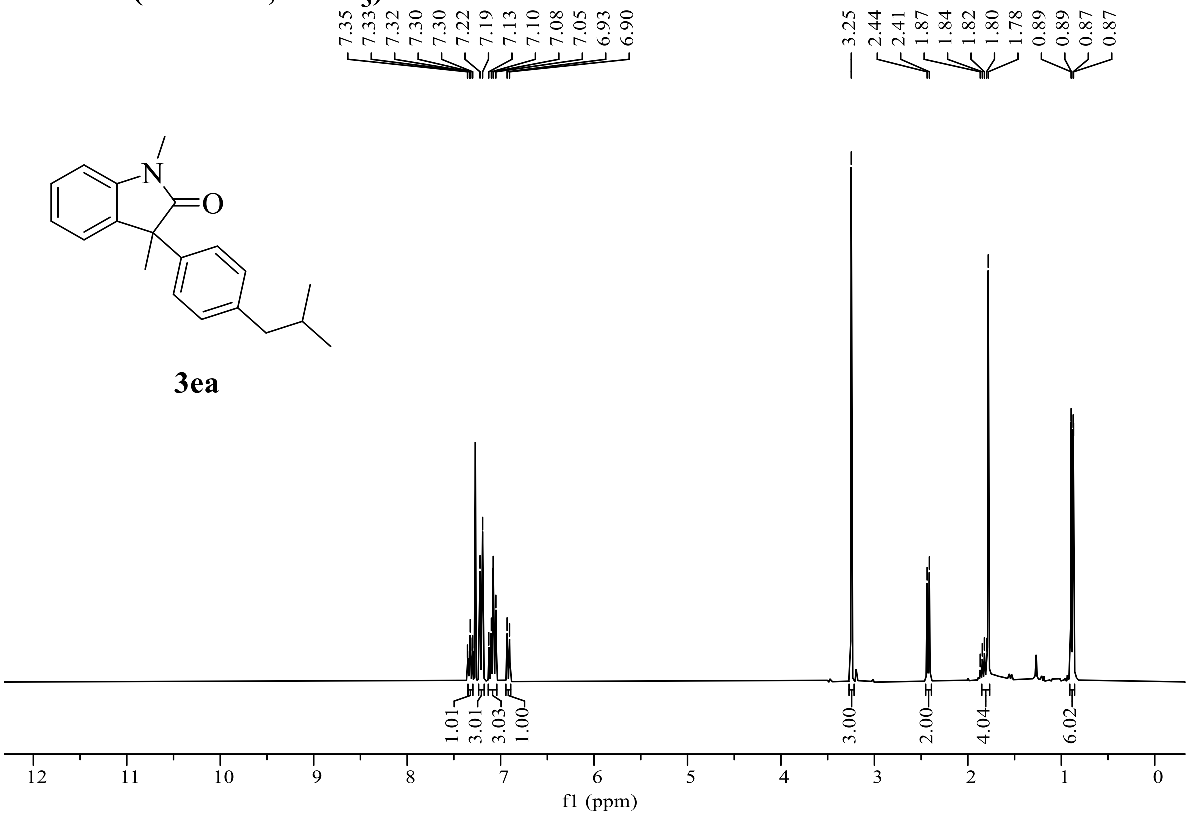

^{13}C NMR (75 MHz, $CDCl_3$):

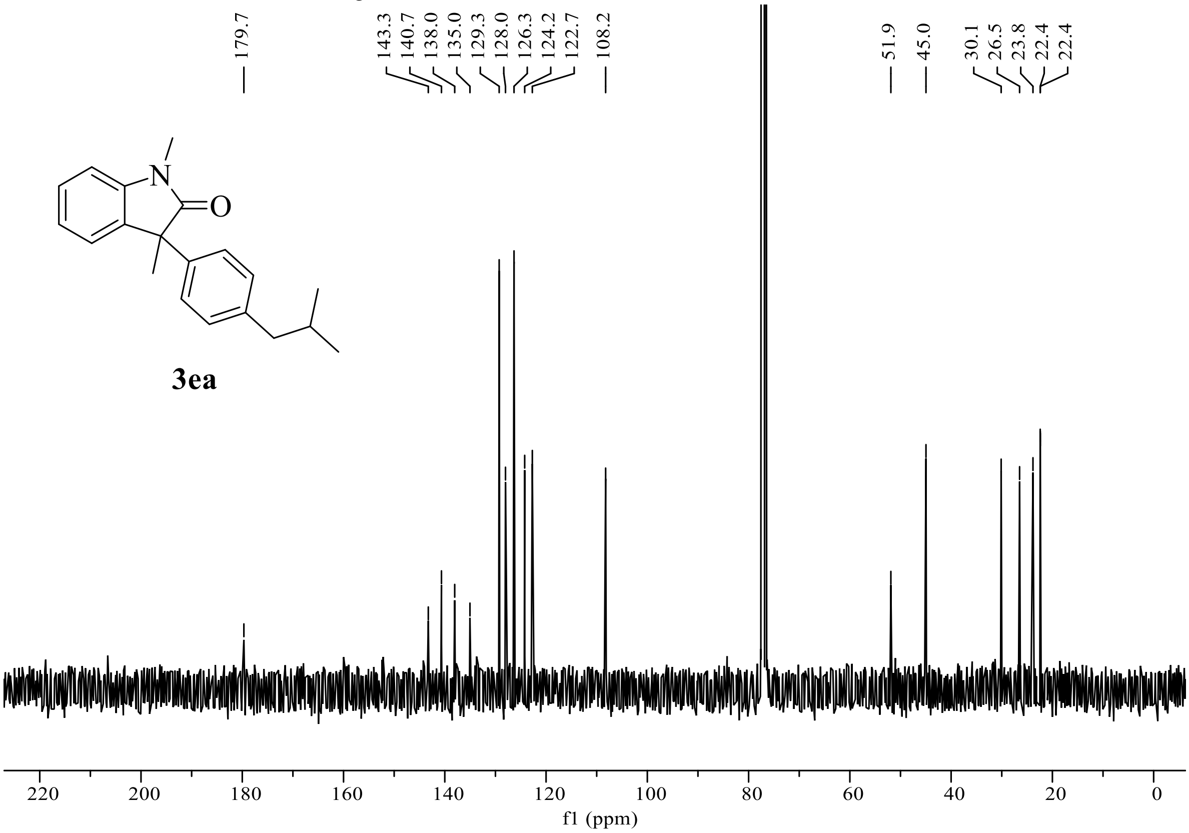

^{1}H NMR (300 MHz, $CDCl_3$):

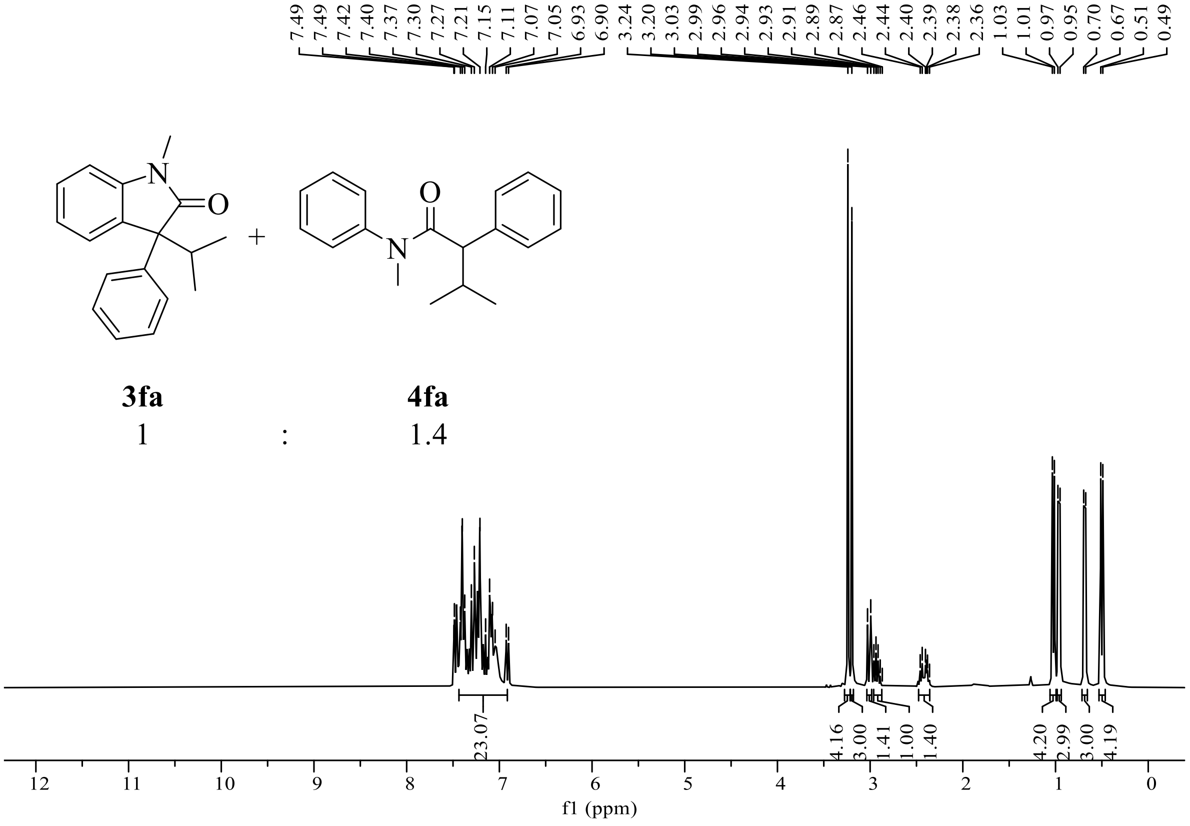

^{13}C NMR (75 MHz, $CDCl_3$):

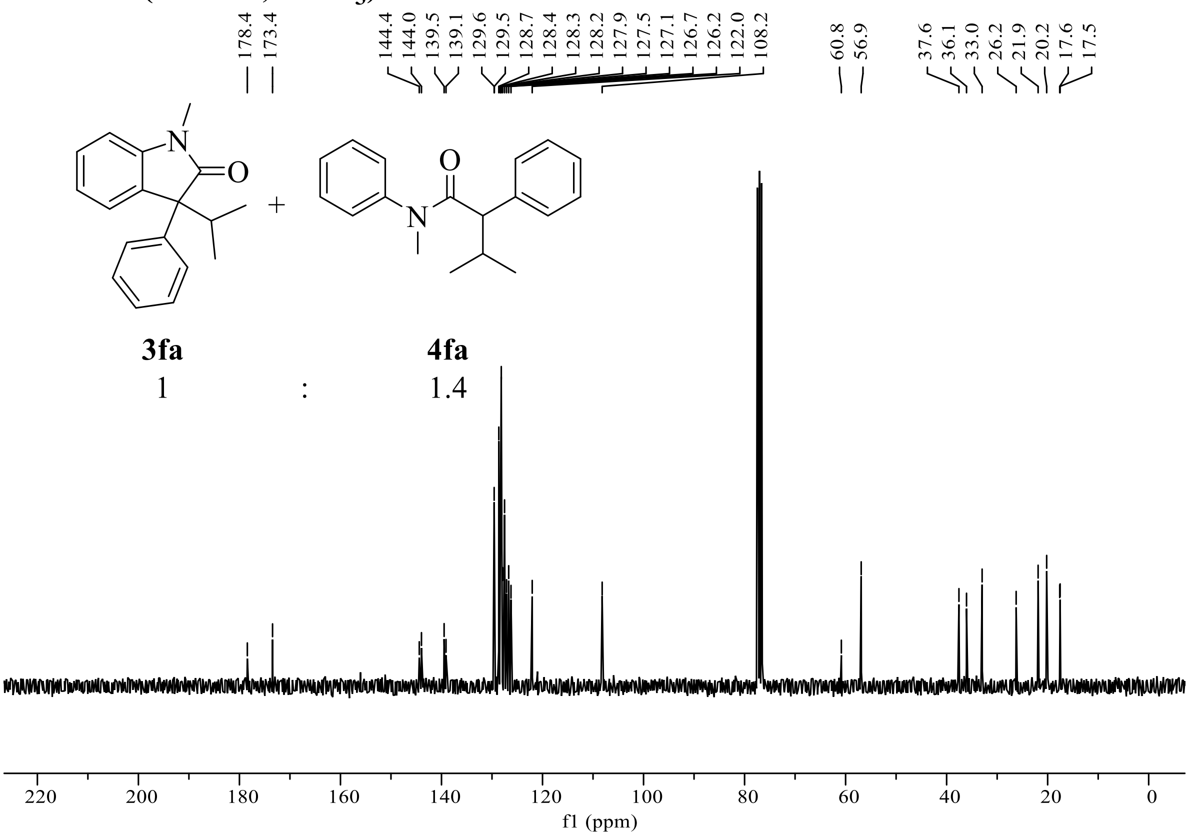

^{1}H NMR (600 MHz, CDCl$_3$):

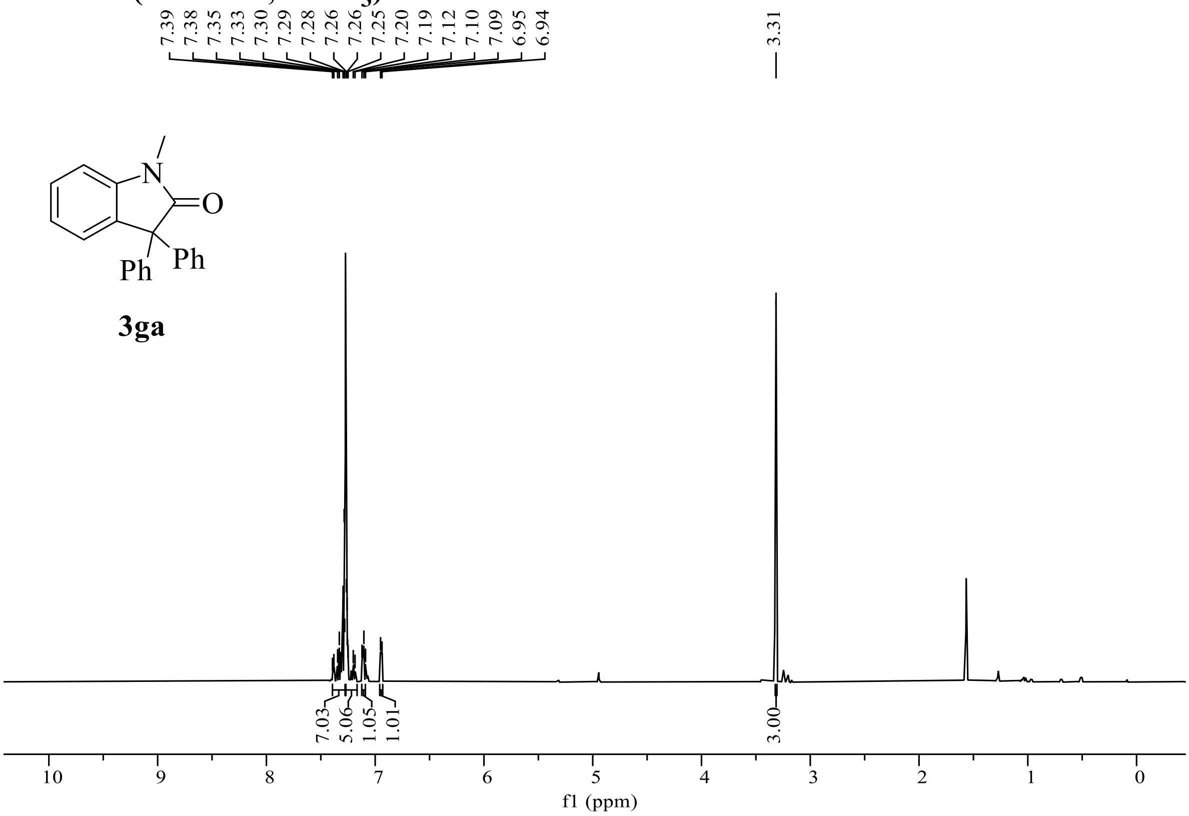

^{13}C NMR (151 MHz, CDCl$_3$):

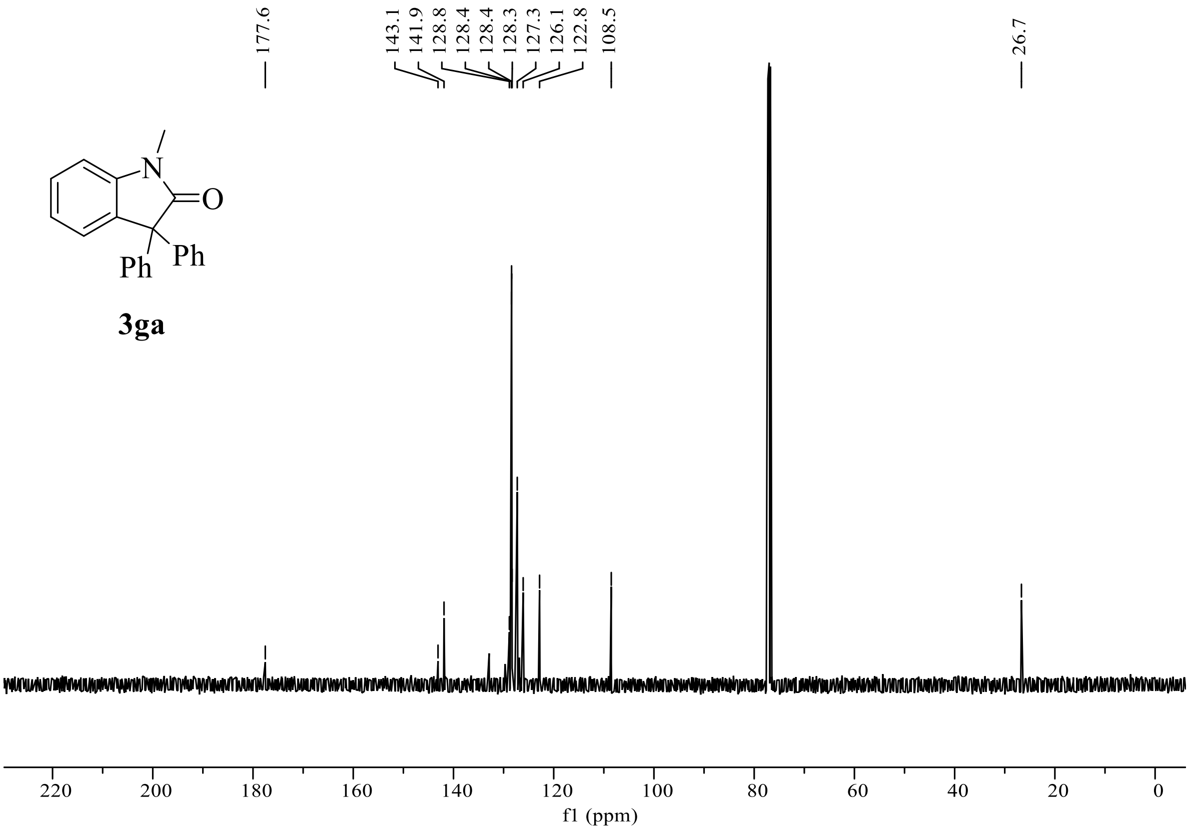

^{1}H NMR (300 MHz, $CDCl_3$):

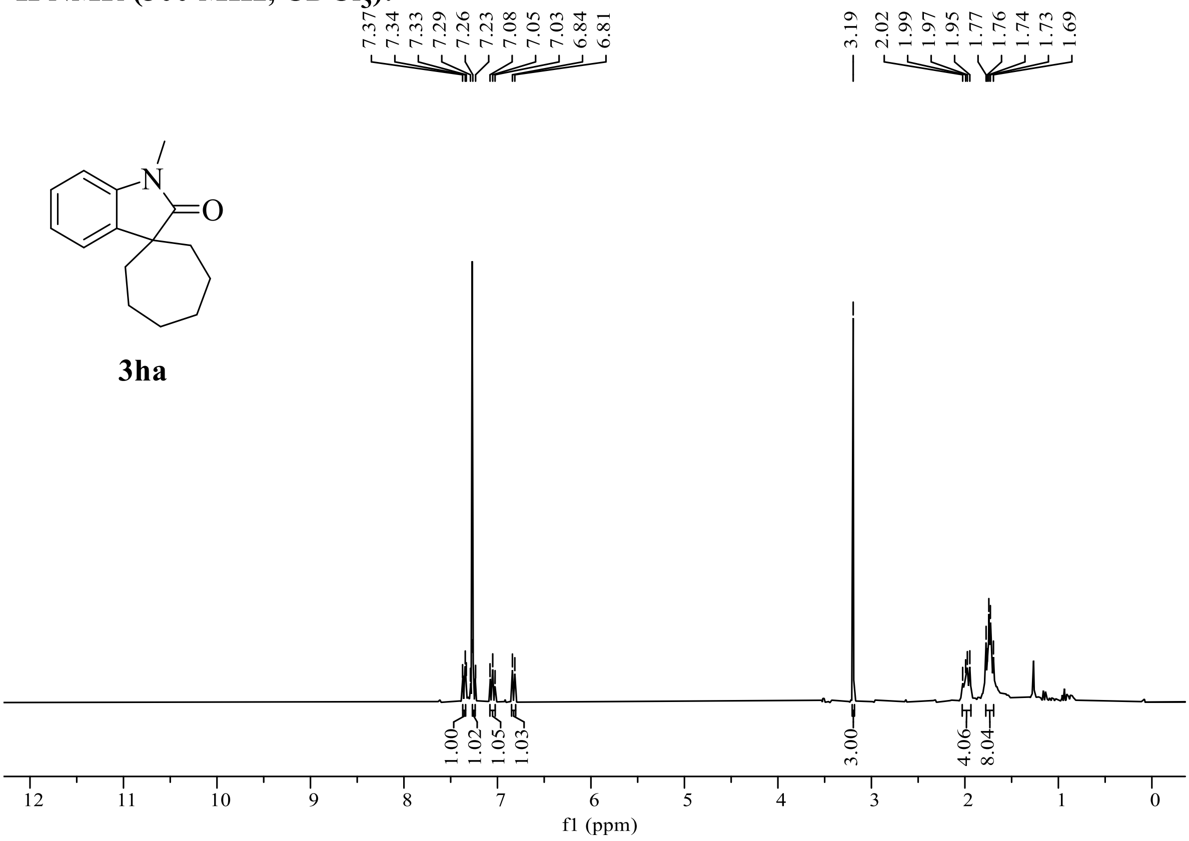

^{13}C NMR (75 MHz, $CDCl_3$):

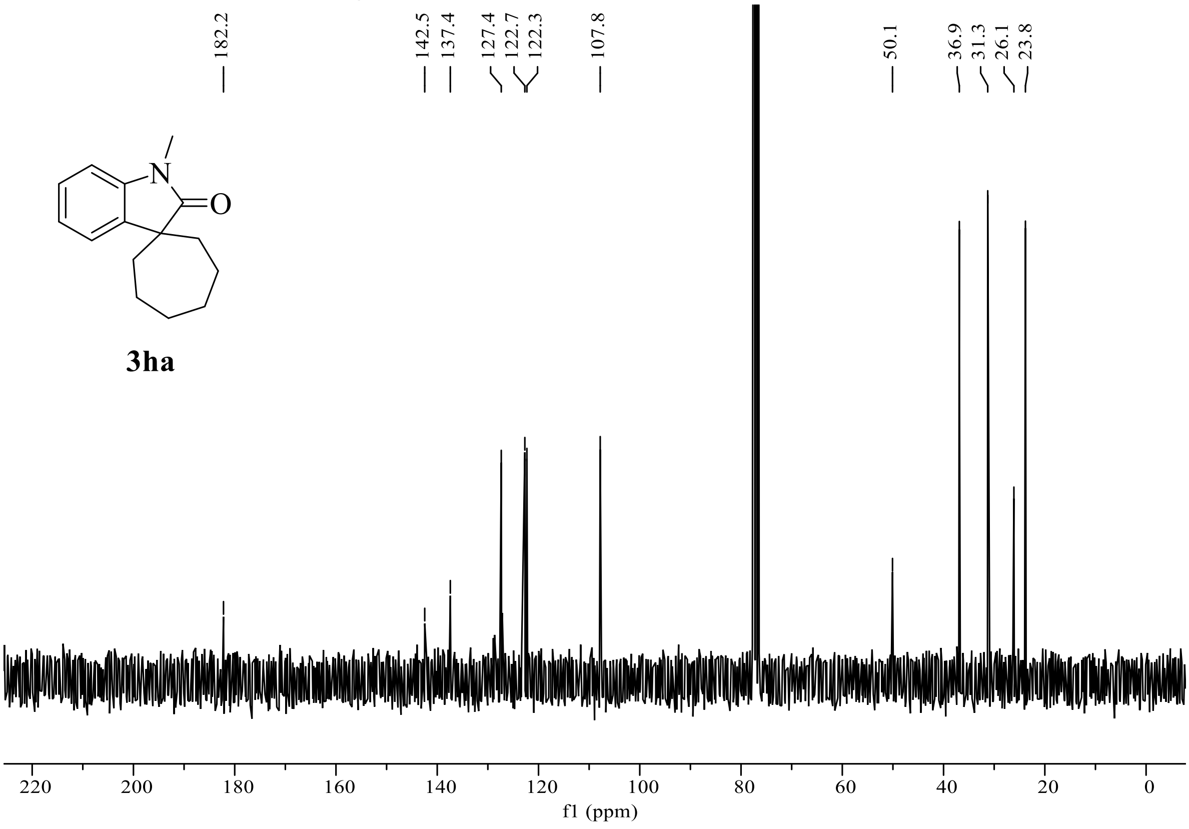

^{1}H NMR (300 MHz, $CDCl_3$):

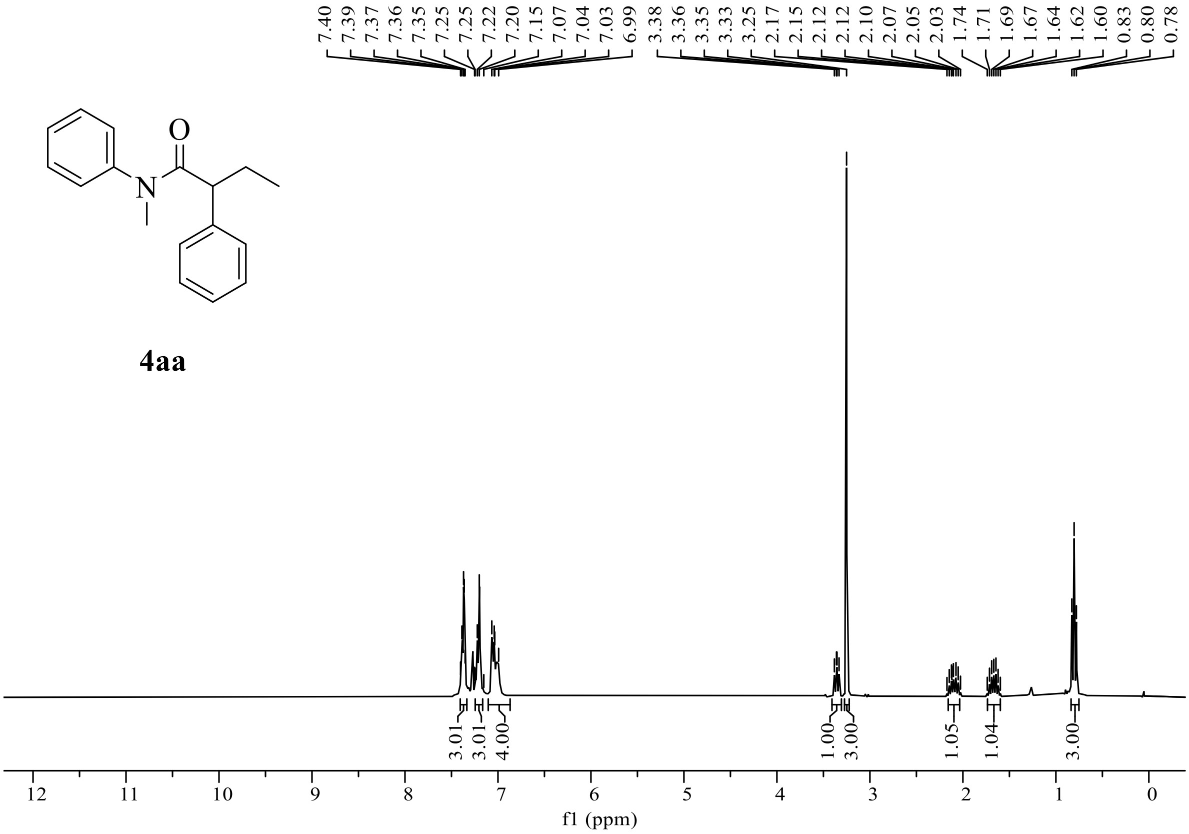

^{13}C NMR (75 MHz, $CDCl_3$):

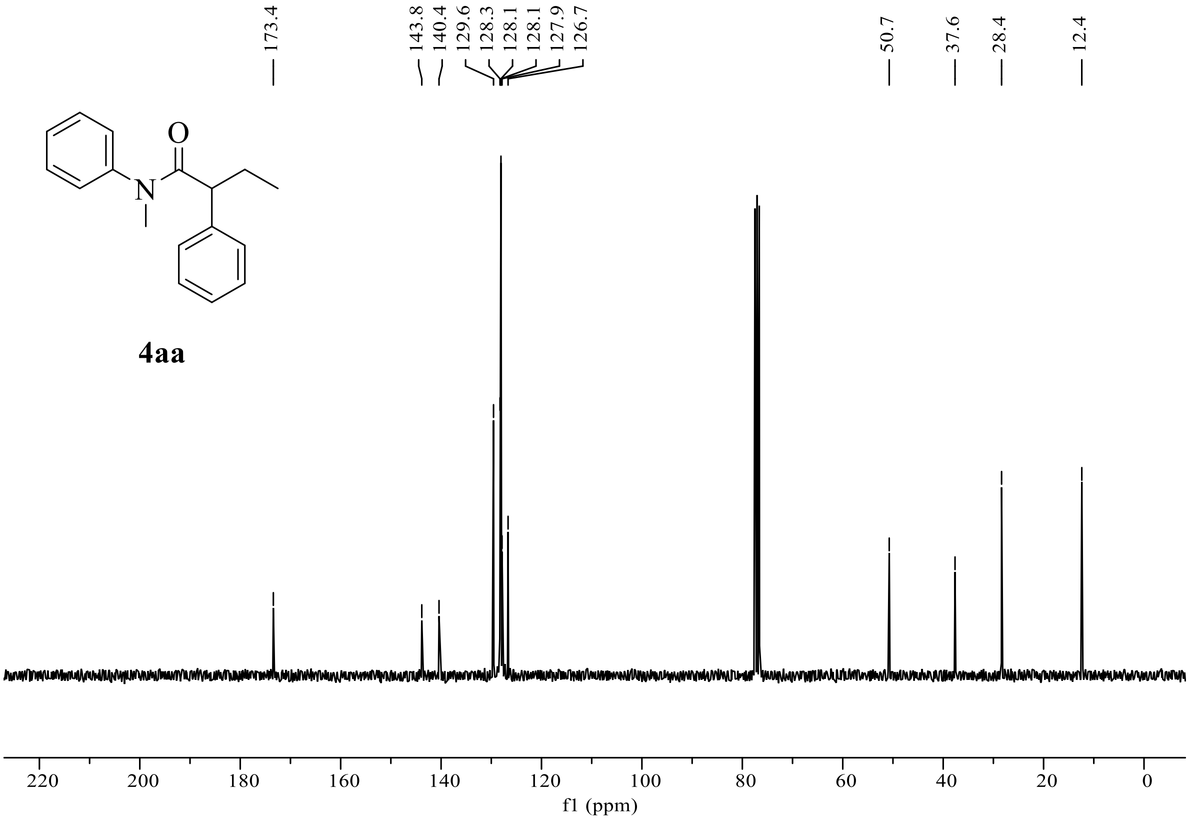

^{1}H NMR (300 MHz, $CDCl_3$):

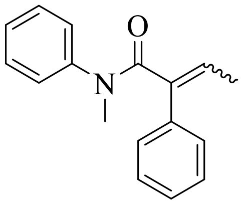

5aa

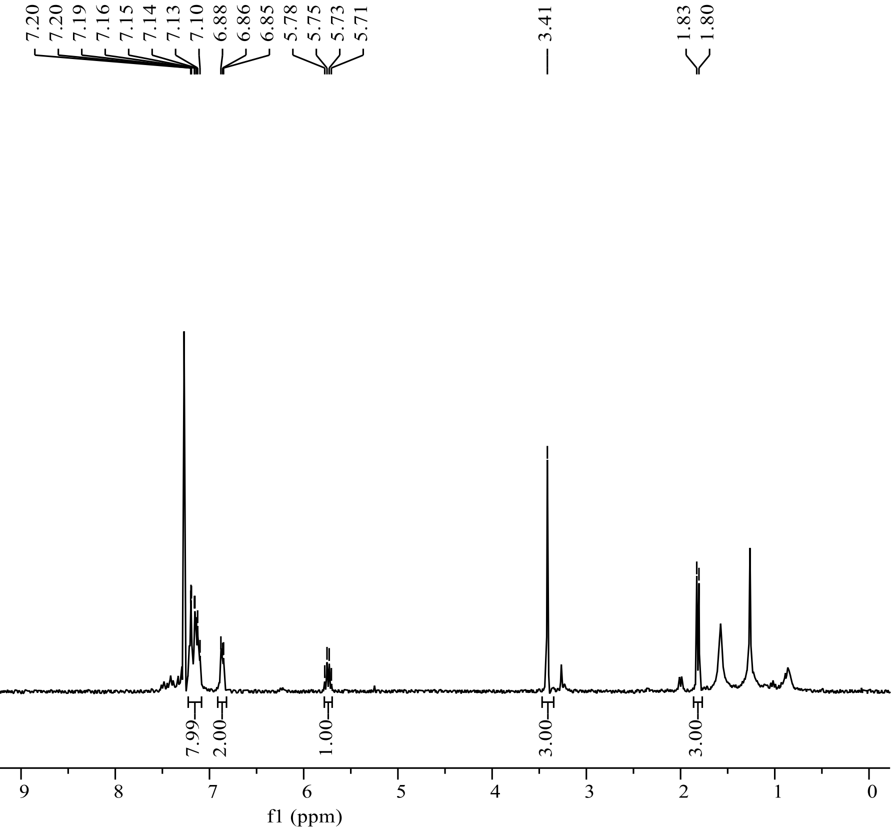

^{13}C NMR (126 MHz, $CDCl_3$):

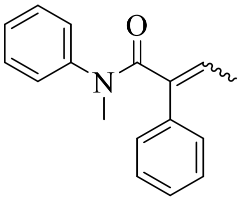

5aa

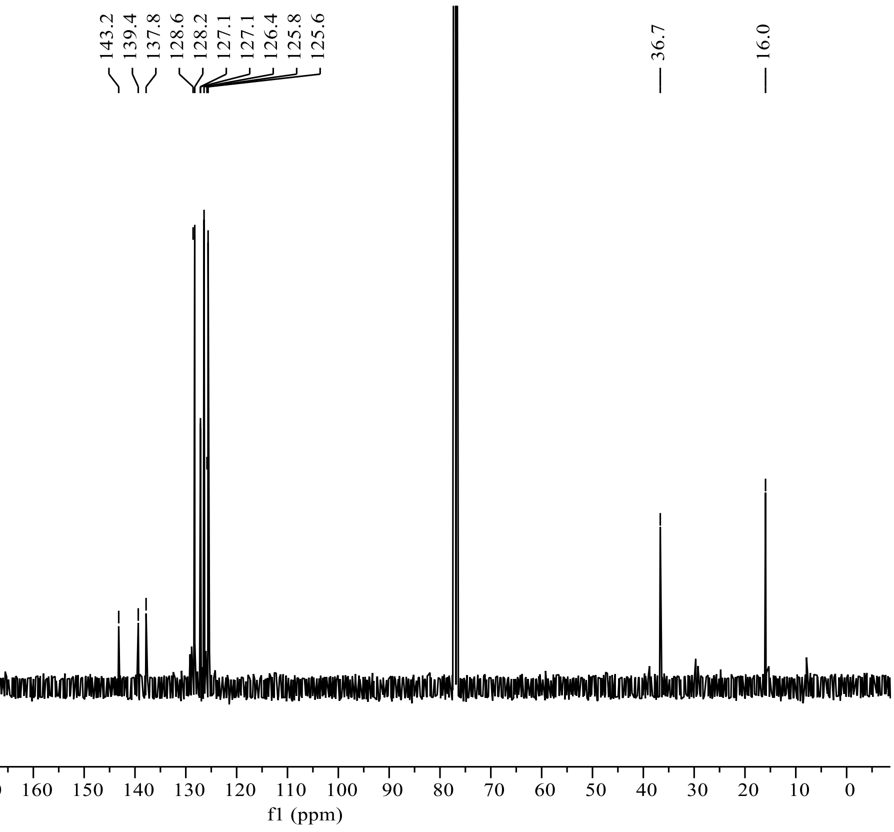

^{1}H NMR (300 MHz, $CDCl_3$):

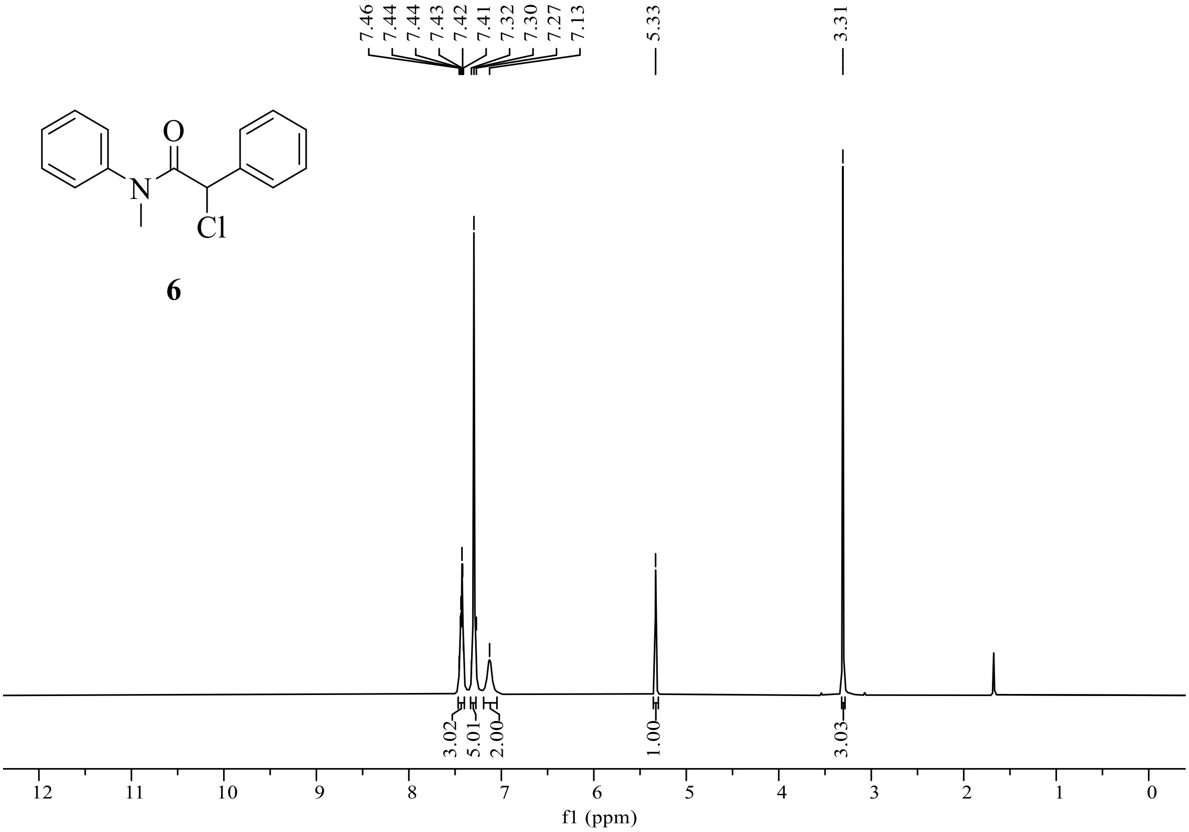

6

^{13}C NMR (75 MHz, $CDCl_3$):

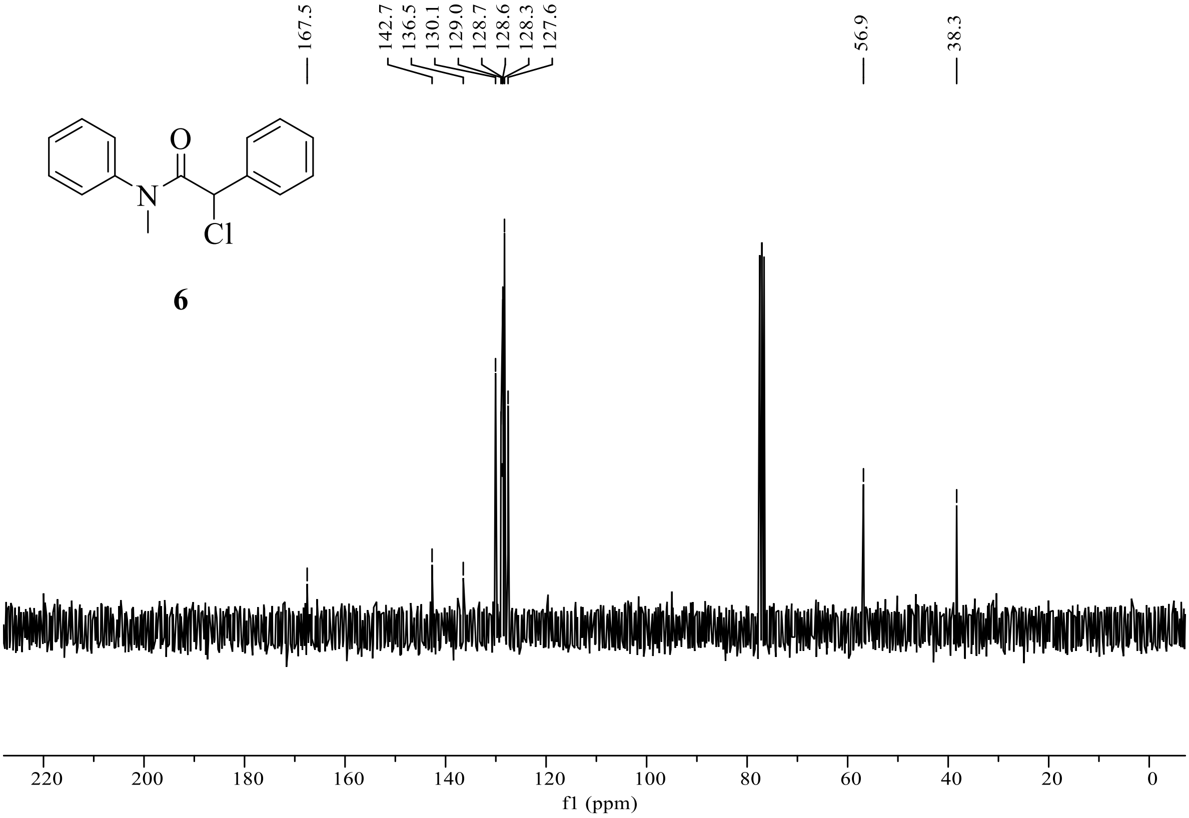

6

^{1}H NMR (300 MHz, $CDCl_3$):

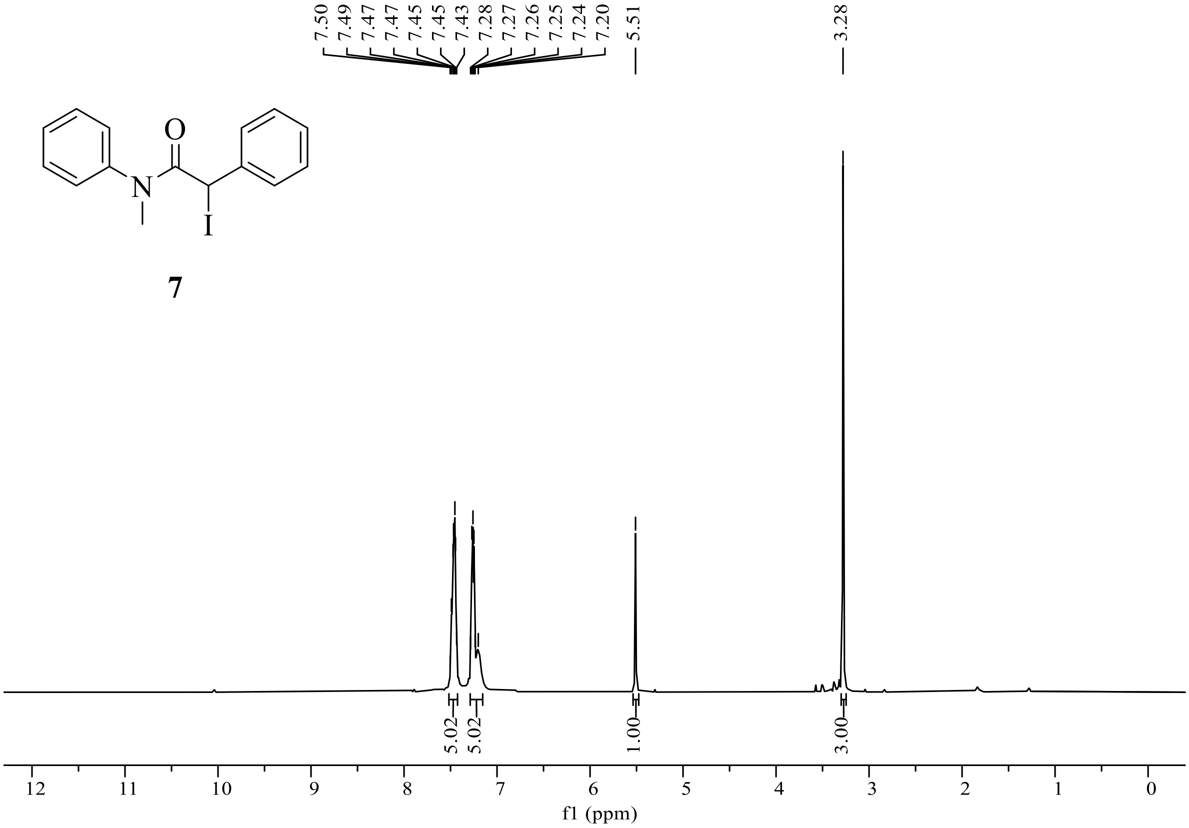

^{13}C NMR (75 MHz, $CDCl_3$):

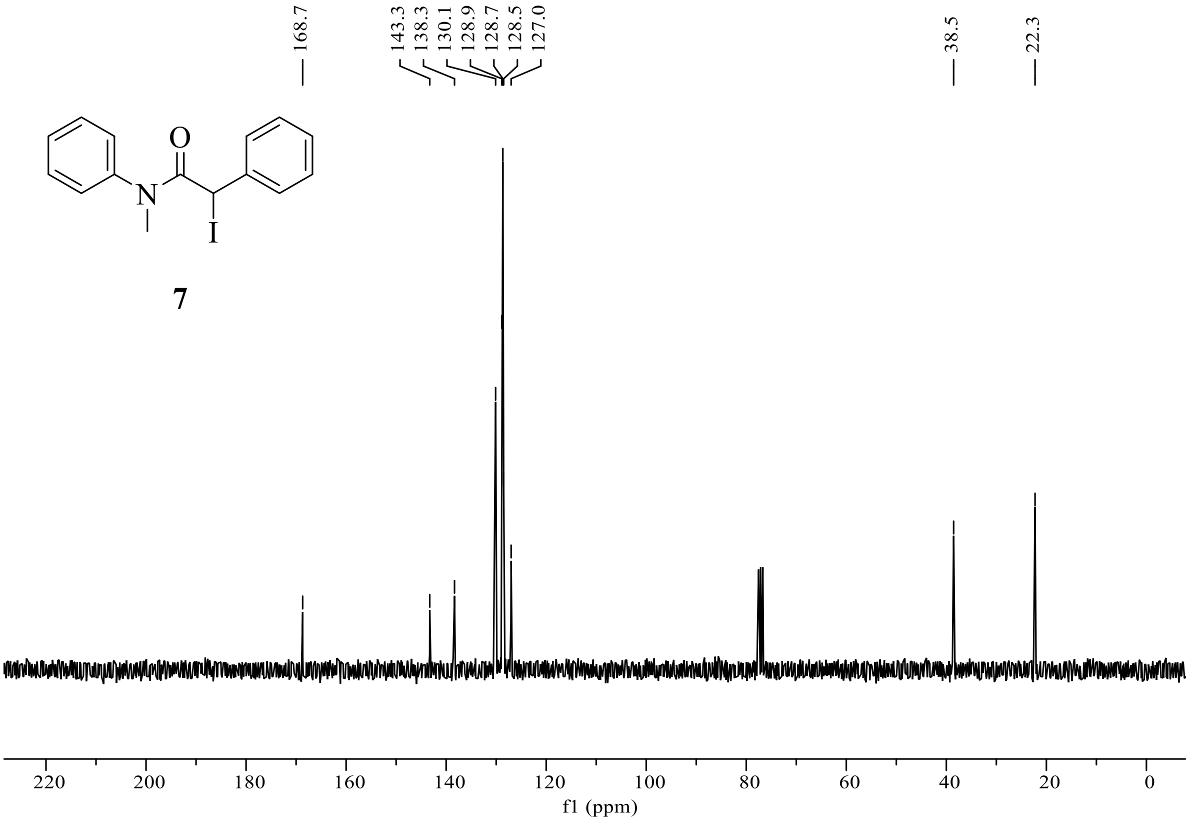

5. ESI(+)-MS spectra of intermediate C

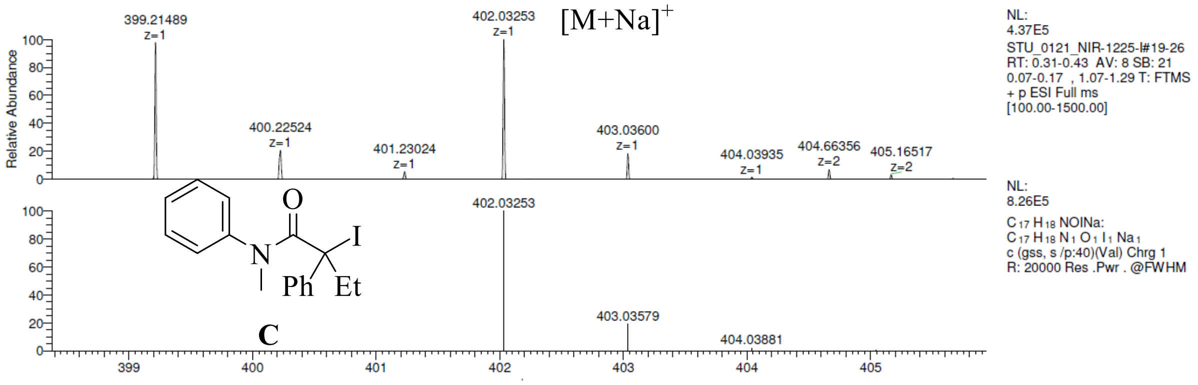

6. References

1 D. Ma, Q. Cai and H. Zhang, *Org. Lett.*, 2003, **5**, 2453.

2 J. Barluenga, A. M. Bayón and G. Asensio, *J. Chem. Soc., Chem. Commun.*, 1984, **0**, 1334.

3 L. M. Baigrie, H. R. Seiklay and T. T. Tidwell, *J. Am. Chem. Soc.*, 1985, **107**, 5391.

4 M. Mewald and M. Oestreich, *Chem. Eur. J.*, 2012, **18**, 14079.

5 J. Tagashira, D. Imao, T. Yamamoto, T. Ohta, I. Furukawa and Y. Ito, *Tetrahedron: Asymmetry*, 2005, **16**, 2307.

6 J. Neumann, S. Elangovan, A. Spannenberg, K. Junge and M. Beller, *Chem. Eur. J.*, 2017, **23**, 5410.

7 E. Falk, V. C. M. Gasser and B. Morandi, *Org. Lett.*, 2021, **23**, 1422.

8 R. Liang, S. Li, R. Wang, L. Lu and F. Li, *Org. Lett.*, 2017, **19**, 5790.

9 A. Kumar, T. Ramani and B. Sreedhar, *Synlett*, 2013, **24**, 938.

10 J. Douglas, J. E. Taylor, G. Churchill, A. M. Z. Slawin and A. D. Smith, *J. Org. Chem.*, 2013, **78**, 3925.

11 J. J. Douglas, G. Churchill, A. M. Z. Slawin, D. J. Fox and A. D. Smith, *Chem. Eur. J.*, 2015, **21**, 16354.

12 H.-D. Xia, Z.-L. Li, Q.-S. Gu, X.-Y. Dong, J.-H. Fang, X.-Y. Du, L.-L. Wang and X.-Y. Liu, *Angew. Chem. Int. Ed.*, 2020, **59**, 16926.

13 A. Roy and M. Oestreich, *Chem. Eur. J.*, 2021, **27**, 8273.

14 A. Viceriat, I. Marchand, S. Carret and J.-F. Poisson, *Org. Lett.*, 2021, **23**, 2449.

15 A. Beyer, J. Buendia and C. Bolm, *Org. Lett.*, 2012, **14**, 3948.

16 N. Hirose, S. Sohda, S. Kuriyama and S. Toyoshima, *Chem. Pharm. Bull.*, 1972, **20**, 1669.

17 S. Würtz, C. Lohre, R. Fröhlich, K. Bergander and F. Glorius, *J. Am. Chem. Soc.*, 2009, **131**, 8344.

18 L. Ackermann, R. Vicente and N. Hofmann, *Org. Lett.*, 2009, **11**, 4274.

19 C. Dey, E. Larionov and E. P. Kündig, *Org. Biom.*, 2013, **11**, 6734.

20 S. Lee and J. F. Hartwig, *J. Org. Chem.*, 2001, **66**, 3402.

21 Y.-X. Jia, D. Katayev, G. Bernardinelli, T. M. Seidel and E. P. Kündig, *Chem. Eur. J.*, 2010, **16**, 6300.

22 R. Samineni, C. R. C. Bandi, P. Srihari and G. Mehta, *Org. Lett.*, 2016, **18**, 6184.

23 M. K. G. Mekhael, S. Bienz, A. Linden and H. Heimgartner, *Helv. Chim.*, 2004, **87**, 2385.

24 Y.-X. Jia and E. P. Kündig, *Angew. Chem. Int. Ed.*, 2009, **48**, 1636.

25 J.-Q. Chen, Y.-L. Wei, G.-Q. Xu, Y.-M. Liang and P.-F. Xu, *Chem. Commun.*, 2016, **52**, 6455.

26 G. Himbert and M. Regitz, *Liebigs Ann. Chem.*, 1973, **9**, 1505.
